# Correction: Genome Wide DNA Methylation Profiles Provide Clues to the Origin and Pathogenesis of Germ Cell Tumors

**DOI:** 10.1371/journal.pone.0132981

**Published:** 2015-07-14

**Authors:** The PLOS ONE Staff

## Notice of Republication

This article was republished on June 24, 2015, to remove the captions for S1 File and S2 File, which did not correspond to files downloadable from the article page. Links to the GEO submission mentioned in the captions have now been incorporated into the article text and tables instead. Please download this article again to view the corrected version. The originally published, uncorrected article and the republished, corrected article are provided here for reference.

## Supporting Information

S1 File Originally published, uncorrected article. (PDF)

S2 File Republished, corrected article. (PDF)
