# Supplementary material for: Genome Wide DNA Methylation Profiles Provide Clues to the Origin and Pathogenesis of Germ Cell Tumors
Source: PLoS One. 2015 Apr 10;10(4):e0122146. doi: 10.1371/journal.pone.0122146 (PMC4479500; doi:10.1371/journal.pone.0122146)
Supplement: S1 Fig — A detailed flowchart and description of the analysis protocol is presented, followed by a visual illustration of the selection of differentially methylated features. The motivation for the threshold when filtering low variability probes is presented next. Finally, the properties of the Hidden Markov Model (HMM) are presented together with a detailed description of is construction. HMM state 15 is presented as a proof of concept, discriminating between male and female samples based on X-chomosomal localization of the large majority of the probes in this class. (PDF) [file pone.0122146.s001.pdf]

methylation in germ cell tumors

supplementary methods – Figure S1

analysis protocol

variance pre-selection

HMM (general)

ANALYSIS PROTOCOL

OVERVIEW

Analysis: Global methylation, differential features & states

Pre-processed data (β and M values)

Visualize & test differences in global methylation profile

- Filter low $\sigma_{M,probes}$
- Per probe MWU
- Verify robustness (stratified bootstrapping)
- Select high ΔM
- subset of probes significantly / relevantly associated with one phenotype

- k state Hidden Markov Model
- Calculate descriptive statistics & median M per segment
  - Filter low $\sigma_{M,segments}$
  - Per segment MWU
  - Verify robustness
  - Select high ΔM
  - subsets of segments significantly associated with one phenotype
  - LASSO on median M per state per sample
  - state(s) significantly associated with one phenotype

"identify probes specific to a histological subtype which also showed patterns consistent with surrounding probes and other, non-adjacent segments on the genome. "

e.g. Identify overlap between probe, segment and state based analysis

description of analysis protocol

First, the global methylation pattern of all feattures and functionally distinct subsets of features we assessed (red block) for all probes and specific functional genomic categories (Figure 1C).

To reduce the number of features and focus on detecting regions of interest rather than only selecting individual differentiating probes (CpG sites) a HMM was trained (see next slides). Depending on the context, feature can mean probe or HMM segment in the text below.

Untimately, a three stage analysis protocol was applied to the data to select probes specific to one subtype which also showed consistent behavior with surrounding probes and non-adjacent regions on the genome that showed similar methylation patterns. Subtypes were compared in a pairwise fashion.

1. Probe-based approach: differences in the distribution of the methylation status between subtypes was tested using a Mann Whitney U test. The probes deemed significantly different between a pair of histological subtypes were validated in 100 bootstrap samples. Probes significant in >=95% of the bootstrap samples as well were deemed significant and robust. A final selection was done based on the median M value within either tumor subtype. If these differed at least ±0.9, feature was called significant, robust and relevant and selected for further study. 0.9 lies in the middle of the cut-off range (0.4-1.4) recommended by Du et al. 2010. A less stringent setting might result in a higher detection rate but considerably reduces the true positive rate (Du et al, 2010, BMC Bioinformatics). The sign of the difference in median M value per subtype was used to assign the relative methylation status (hyper/hypo) in each of the subtypes.
2. Segment-based: the segments resulting from a k state Hidden Markov Model (HMM) were subjected to the analysis described under (1) in order to select groups of probes that showed a consistent pattern within a genomic region rather than for a specific probe. The HMM is discussed extensively in the next slides.
3. State-based: a LASSO regression model was fitted to the median methylation values of all probes in each state in each sample. States remaining after shrinkage were deemed significantly different between the subtypes under investigation. The sign of the difference in median M value per subtype was used to assign the relative methylation status (hyper/hypo) in each of the subtypes.

By indentifying the overlap between 1, 2 & 3 (blue block in protocol), probes specific to one subtype were selected which also showed consistent behavior with surrounding probes and non-adjacent regions on the genome that showed similar methylation patterns. These subset specific probe lists were studied further (see next slide).

Abbreviations: σ: standard deviation of the M values per feature as observed over all samples. MWU: Mann Whitney U test. ΔM: the difference between the median M values of a specific probe in the two sample groups under consideration. k: the number of states in the Hidden Markov Model. LASSO: Least Absolute Shrinkage and Selection Operator. LASSO is a shrinkage and selection method for linear regression (bound on the sum of the absolute values of the coefficients)

GCT type A vs B: differentially methylated probes (DMP)

DMPs are indicated as either
relatively hypermethylated in A (DMP[**A**-B]) or
relatively hypermethylated in B (DMP[A-**B**])

DMP[**A**-B]

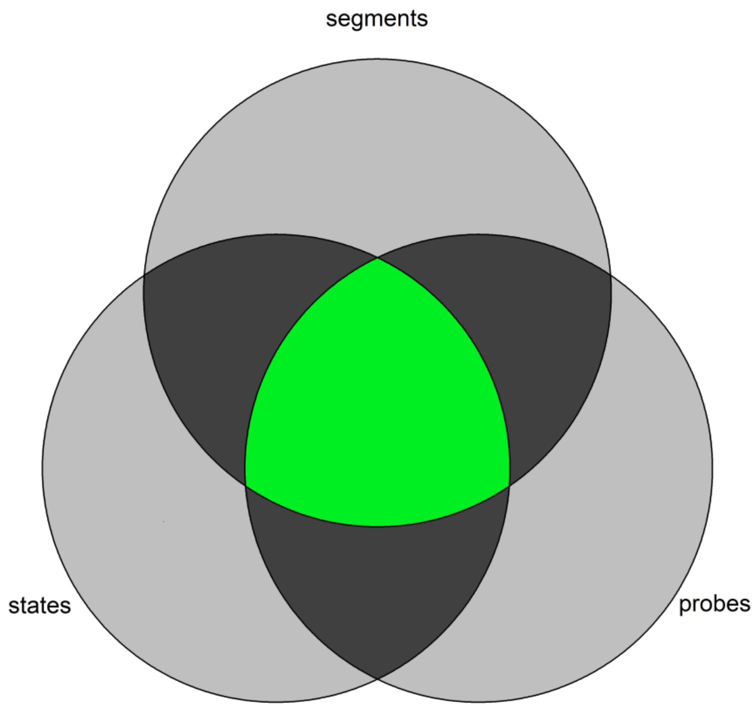

DMP[A-**B**]

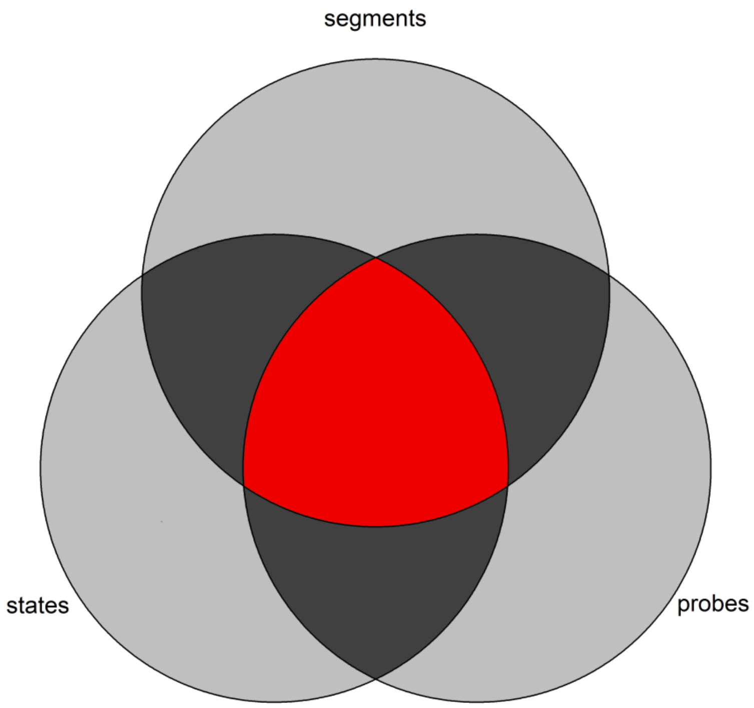

A real life example: (A) SE/DG vs (B) SS

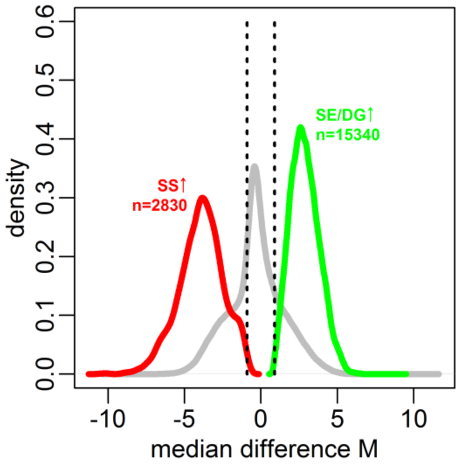

DMP[**SE/DG**-SS]

DMP[SE/DG-**SS**]

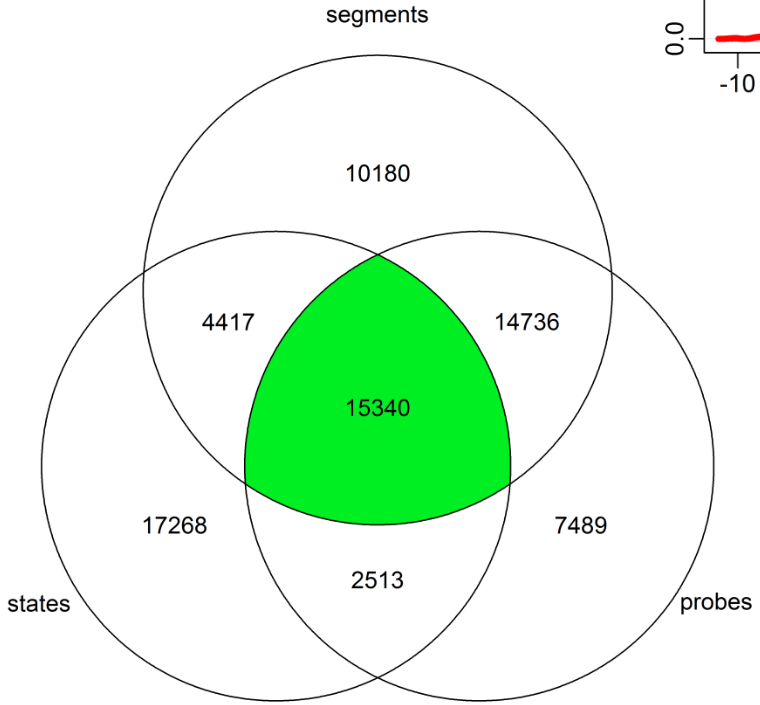

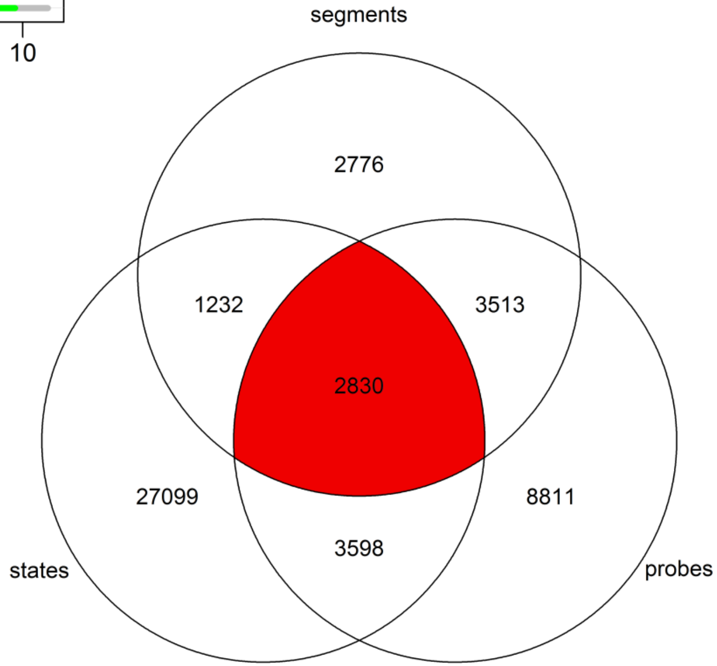

ANALYSIS PROTOCOL

DIFFERENTIAL FEATURES

LOW VARIABILITY PROBES

Probes with a low standard deviation were excluded from the pairwise comparison of features between histological subgroups. Based on the bimodal distribution of the standard deviation of M for both the probes and the segments, the following cut-offs were chosen: σMprobes<0.8, n=77,154/437,881 (17,62%) & σMsegments<0.6, n=13,229/133,730 (9,89%).

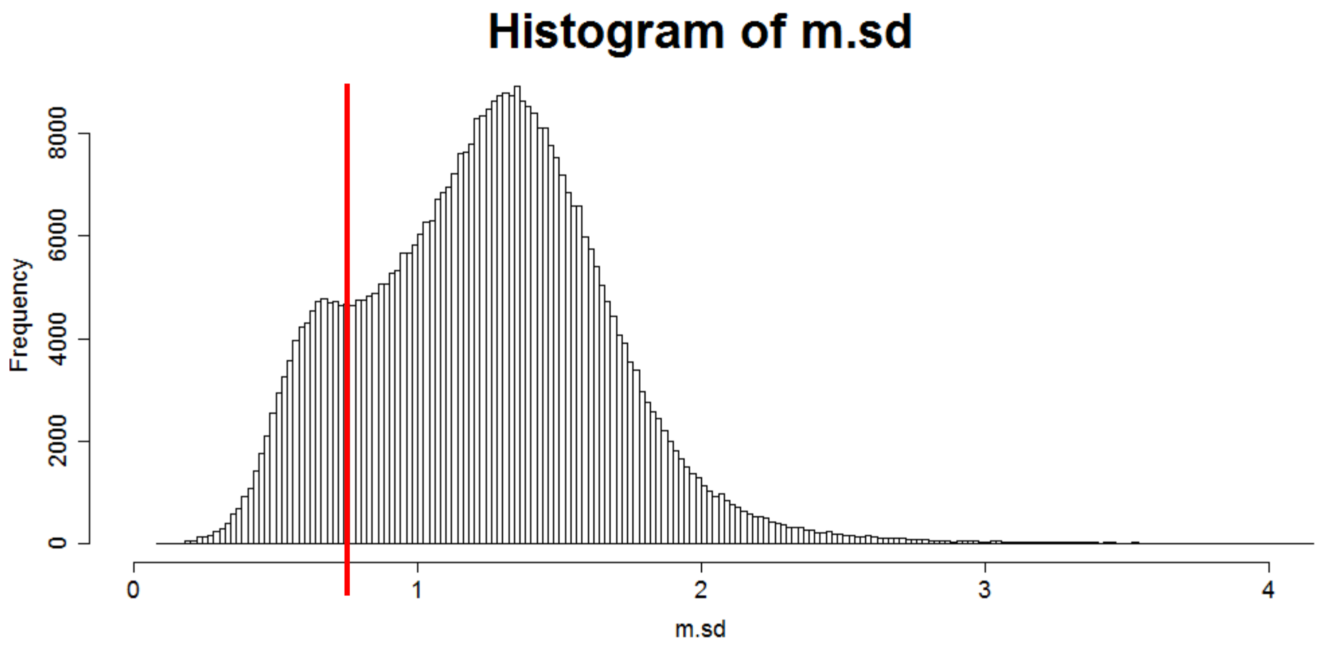

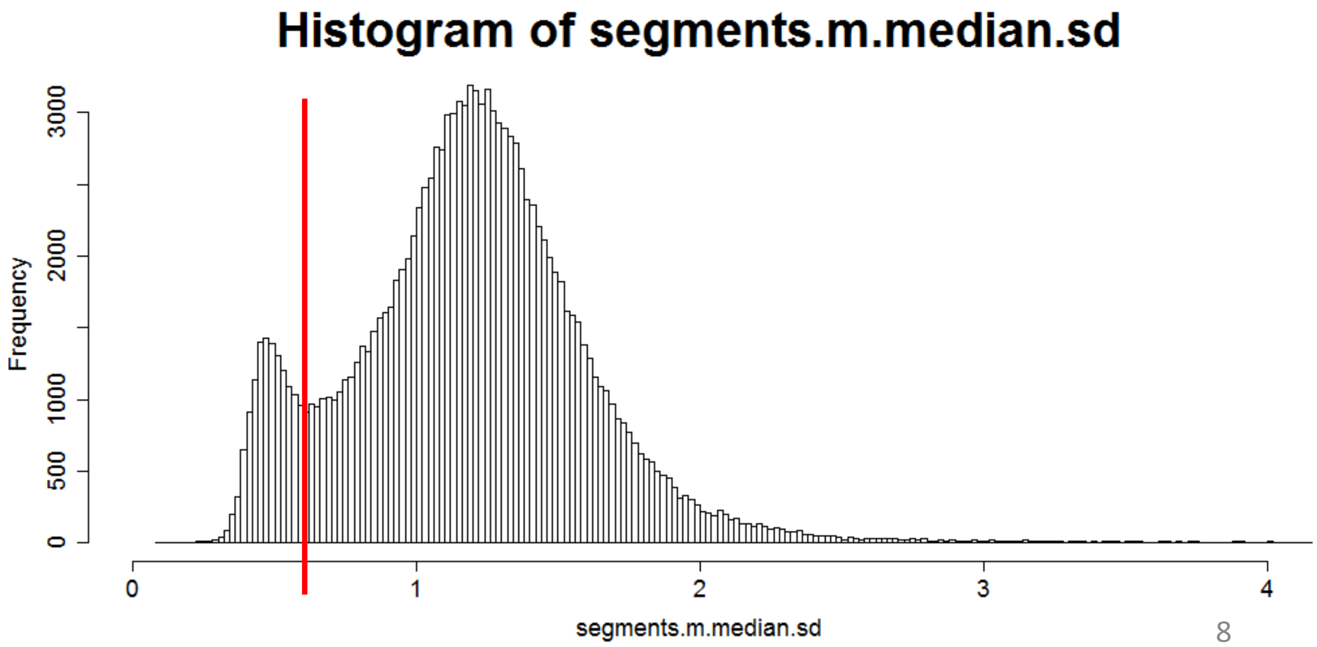

ANALYSIS PROTOCOL

HIDDEN MARKOV MODEL

Example Hidden Markov Model (HMM)

Detailed plot of an arbitrarry genomic region. For each probe, the methylation % (0-100%) is visualized in a green-white-grey-red gradient (median of sample groups where applicable). Probes are depicted as black bars and connected to the relevant part of the heatmap by grey connectors. Patterns are detected using a HMM: adjacent probes with a similar profile are grouped in one segment (grey blocks) and assigned to a state (connected blocks). The HMM is fitted using the MOGHMM toolbox for Matlab, http://prlab.tudelft.nl/david-tax/othersoftware.html . The HMM is fitted on methylation data that is normalized to have unit variance, and each state of the HMM models a single Gaussian distribution. To avoid stability problems during the inversion of the covariance matrix, a regularization is applied that adds a small value of 0.01 to the diagonal of the covariance matrix. To optimize the HMM parameters, the standard Baum–Welch algorithm is used. The optimization is stopped after 250 iterations or when the log-likelihood decreases with a fraction of 10E-5 or less. State numbers are arbitrary. GC% was retrieved from UCSC and is depicted as a histogram (y-axis: 0-100%). Annotated transcripts (if any) will be visualized below the GC%.

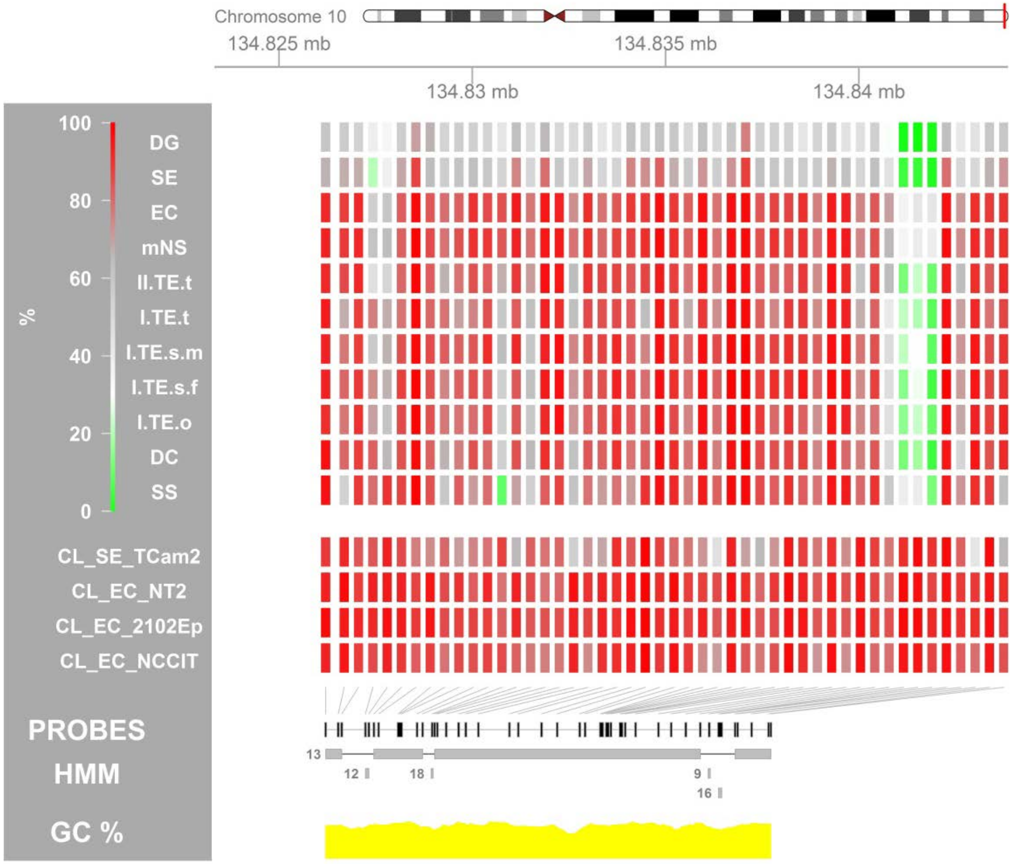

determination of number of states

- Δlog-likelihood (LL) saturates around k=20, indicating that more states do not contribute to the quality of the model.

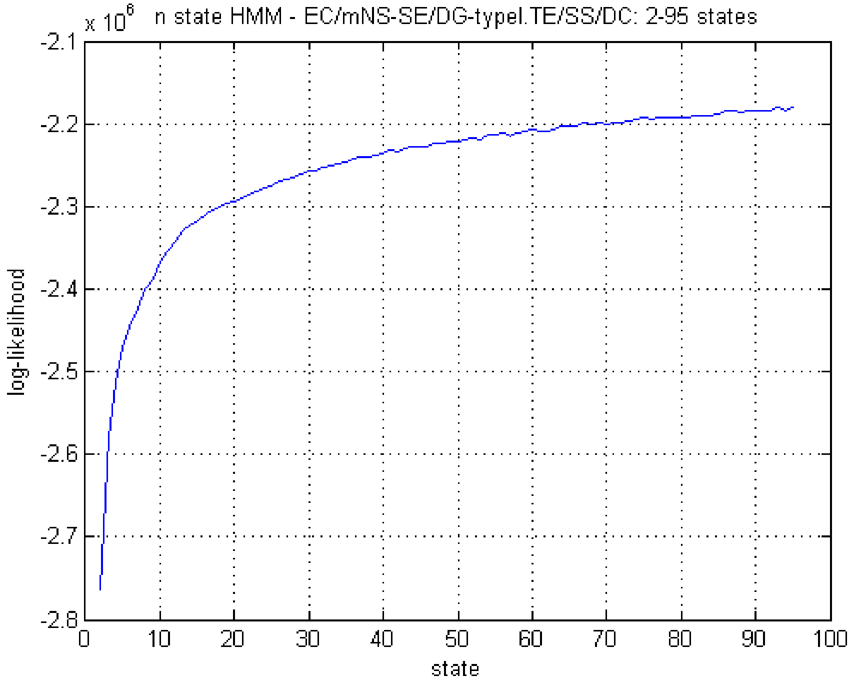

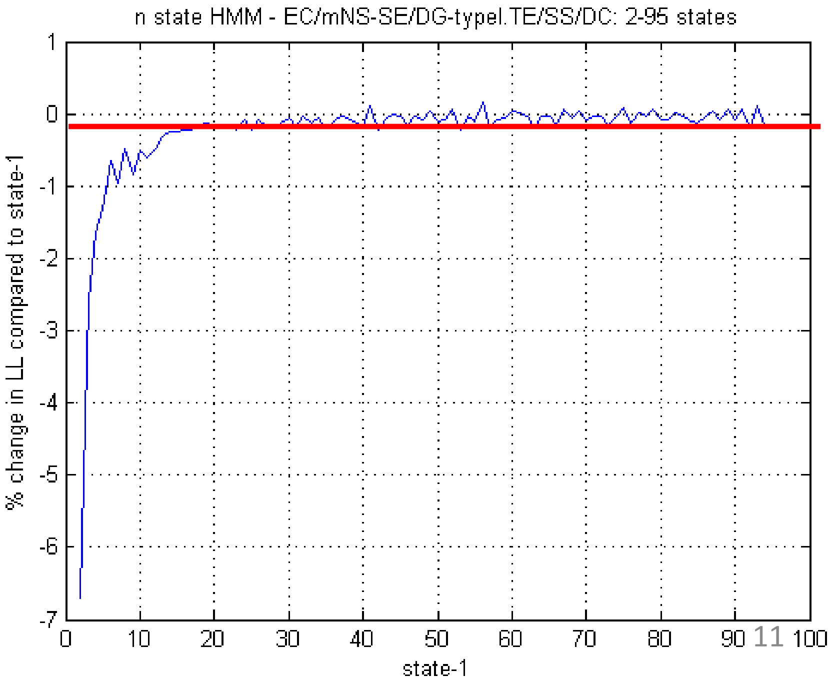

number of probes per state

- Most states incorporate more or less than the expected number of probes, indicating non-random assignment of probes to specific states. Obvious outliers are states 3, 4, 9, 13 and 20 with > 30 000 probes per state. States 5, 12, 15, 16, 17 each incorporate < 10 000 probes per state. (red line=proxy, , cutoffs are arbitrary)

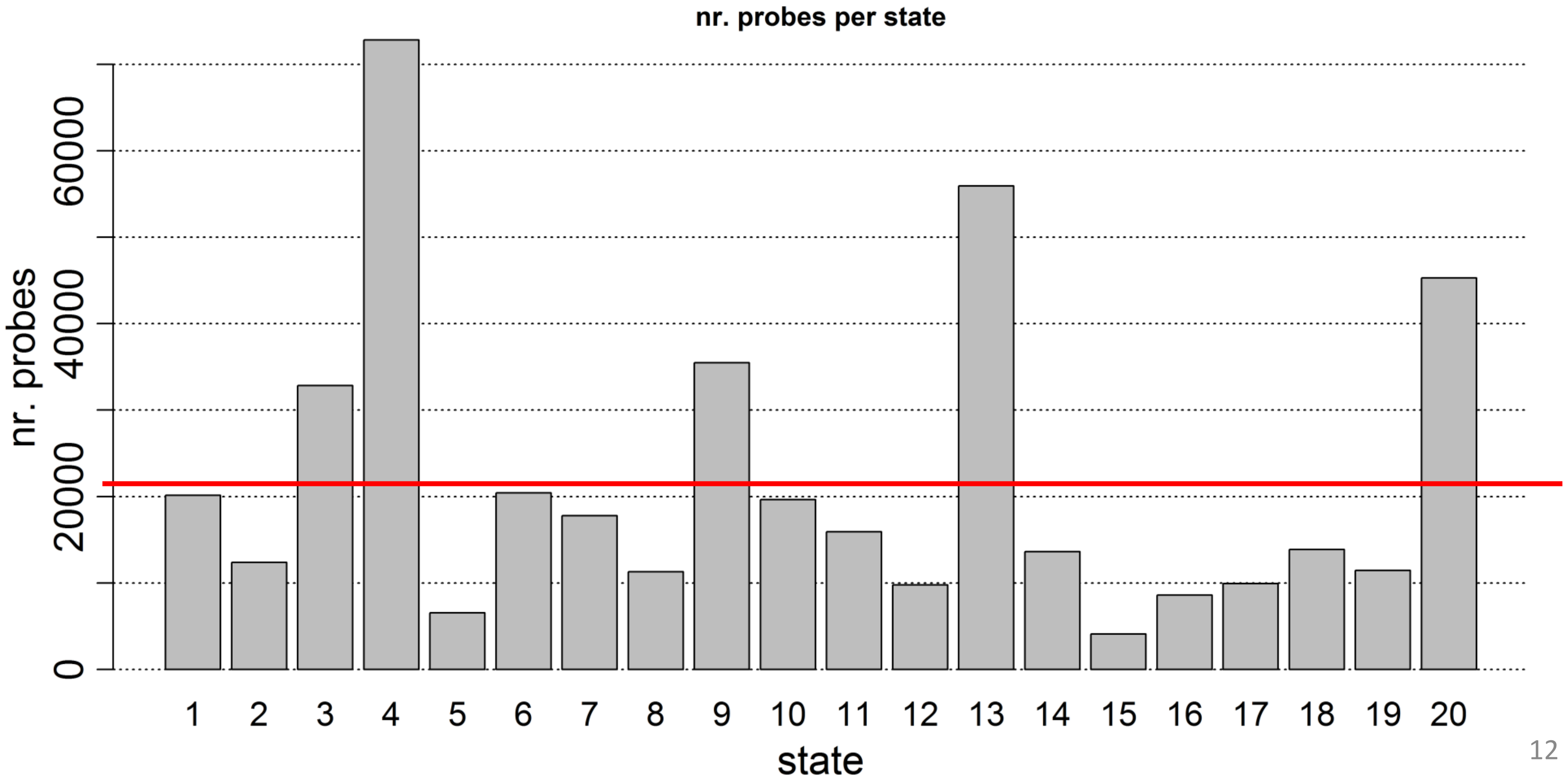

expected: 437881/20=21894.05

number of segments per state

- Most states incorporate far more or less than the expected number of segments, indicating non-random generation of segments. Obvious outliers are states 3, 4, 11 and 13 with > 10 000 probes per state. States 5,7,15,17 each incorporate ca. < 2500 segments per state. (red line=proxy, cutoffs are arbitrary)

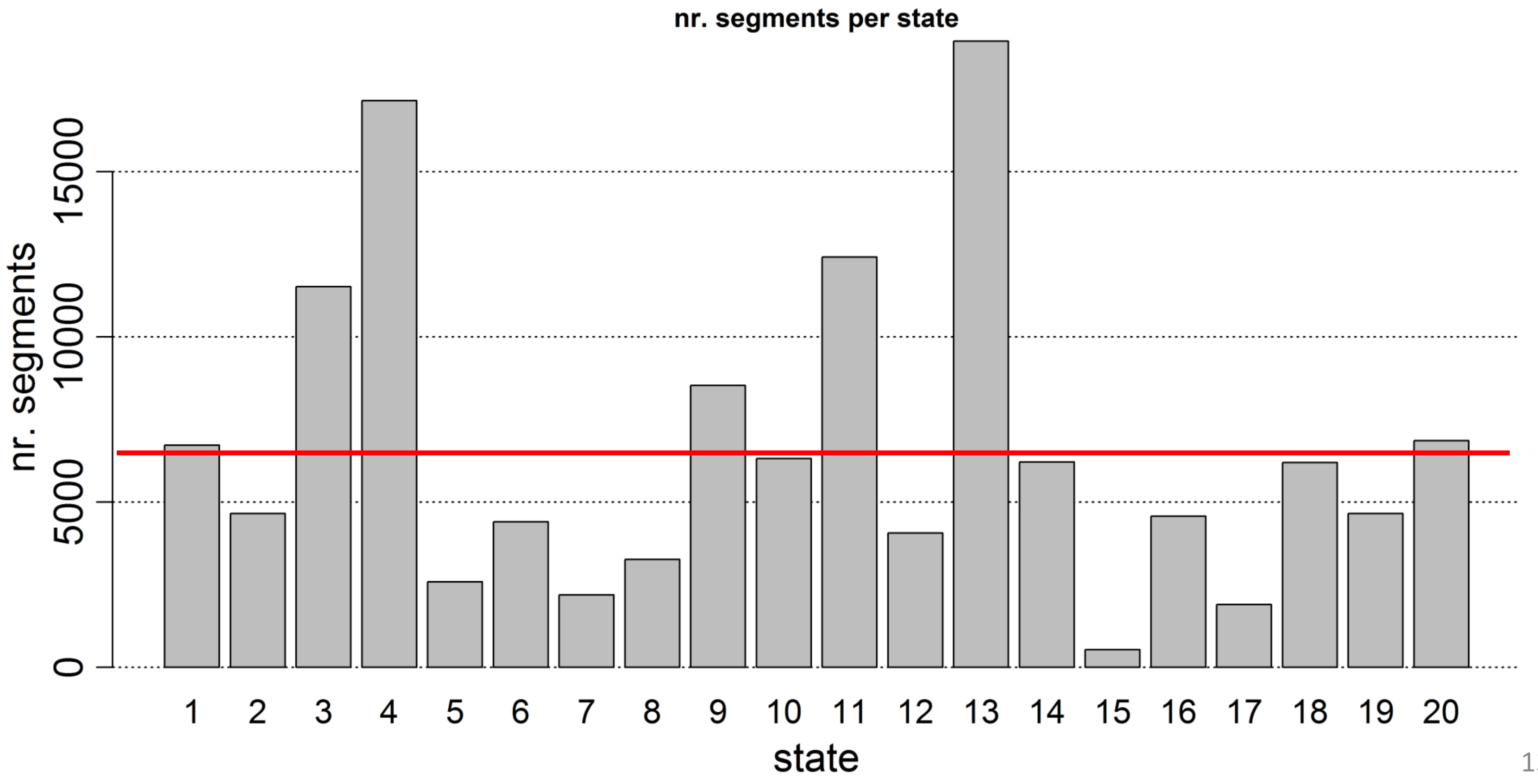

expected: 133,730/20=6686.5

number of probes in segments per state

- The outlier states in the number of segments per state and the outlier states in the number probes per state differ. This possibly indicates a difference in the number per probes per segment.
- Indeed the number of probes per segment varies strongly between states. In general, the median number of probes per segment is <5 but highly heterogeneous in specific states, e.g. 7, 9, 13 and 20 which all include segments with a high (>50) number of probes.

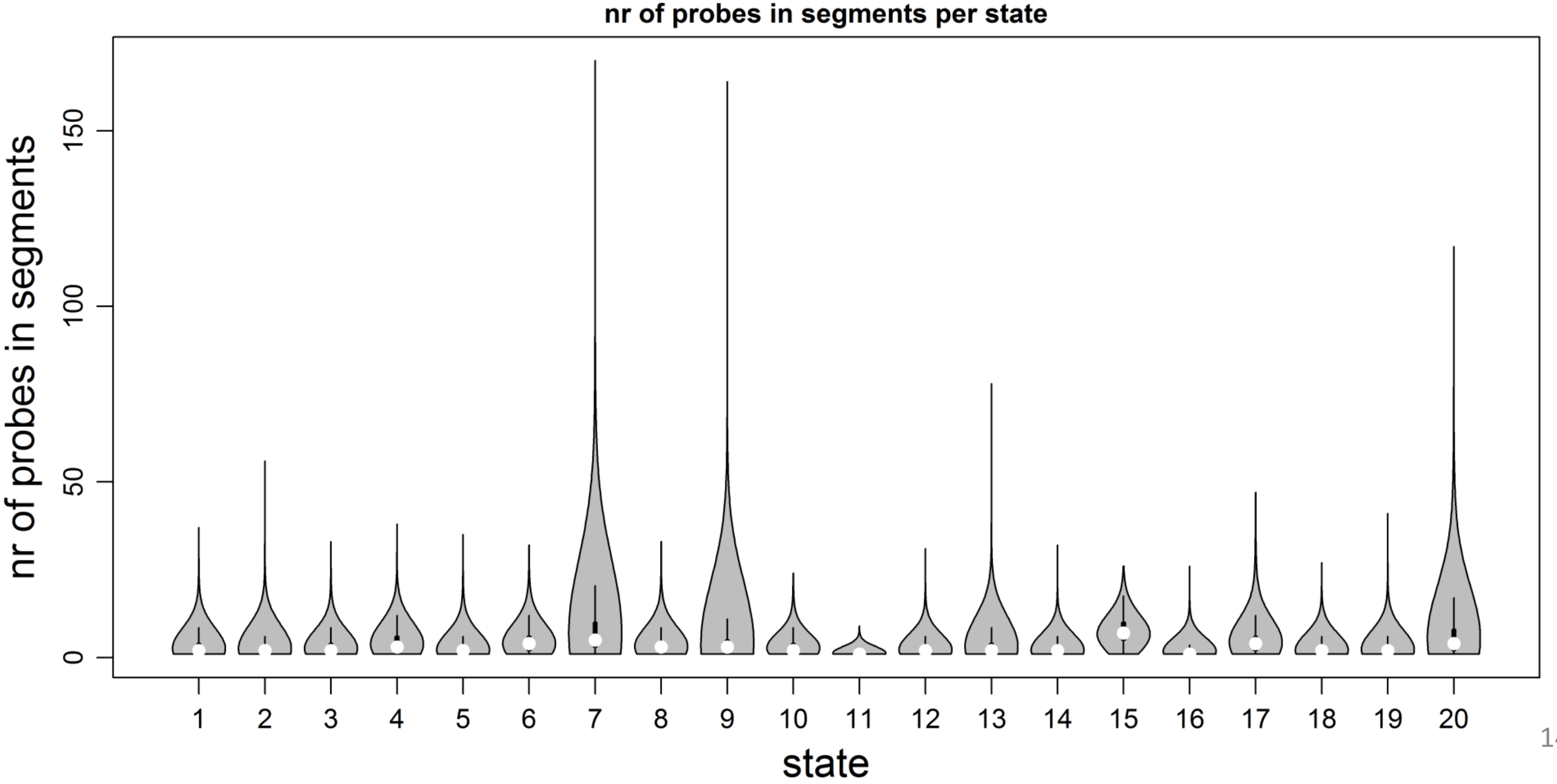

- Please note the log10 scale of the y-axis. The median segment length varies considerably. State 7 shows a concentration of long segments as do 4, 6, 15, 17, 20 to a lesser extend. States 11 and 17 mainly consist of single probe segments. The other states have intermediate median segments lengths but their inter quartile range includes 1bp length (visual assessment on log scale!).

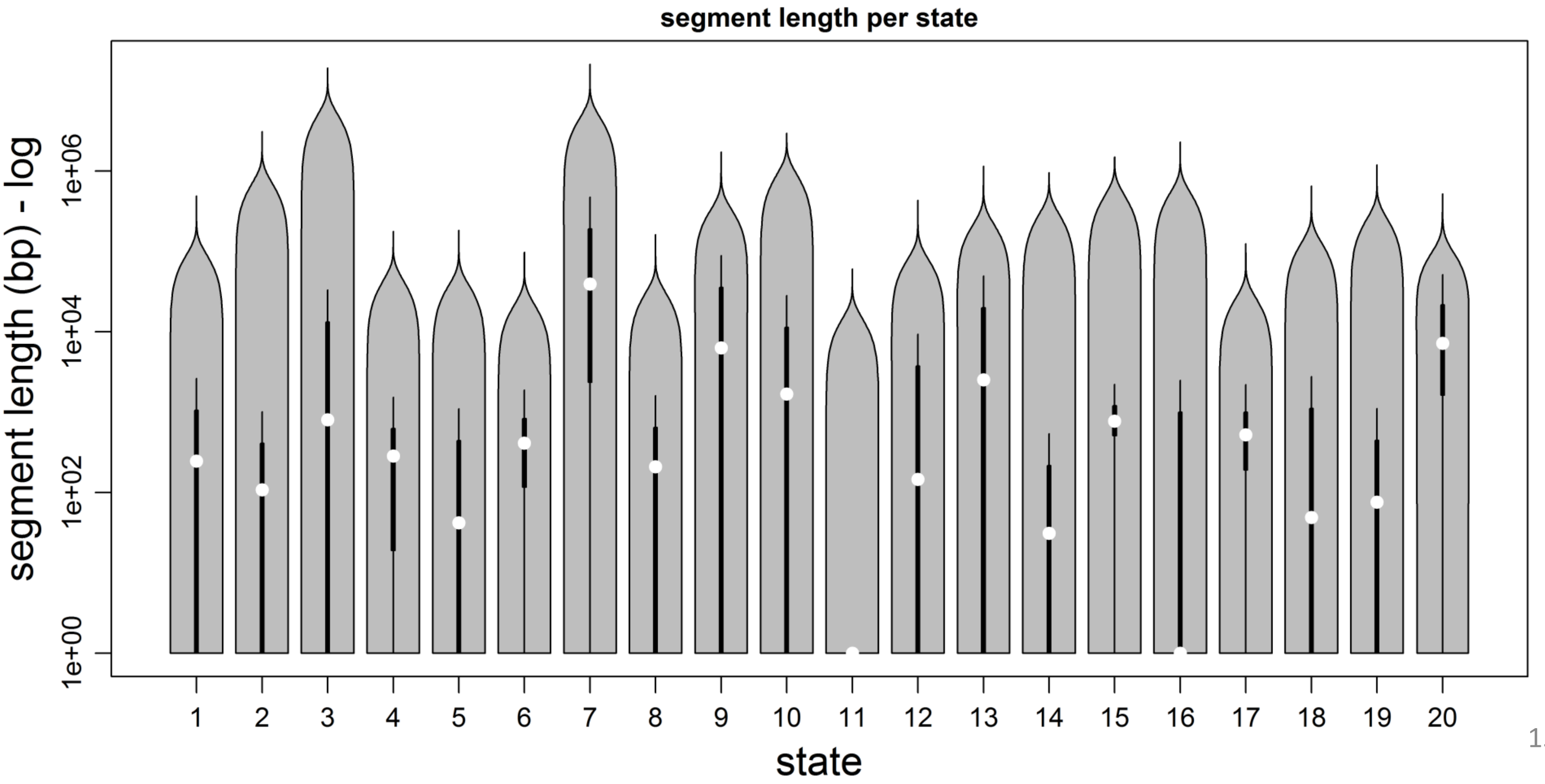

chromosomal distribution

- The large majority of all probes in stat 15 are mapped to X chromosome. This is consistent with the single copy of this chromosome in most of the tumors (male patients) with the exception of the DG, DC and some type I TE. The rest of the states show now obvious overrepresentation of one or more chromosomes (although some reached statistical significance because of the high numbers of probes).

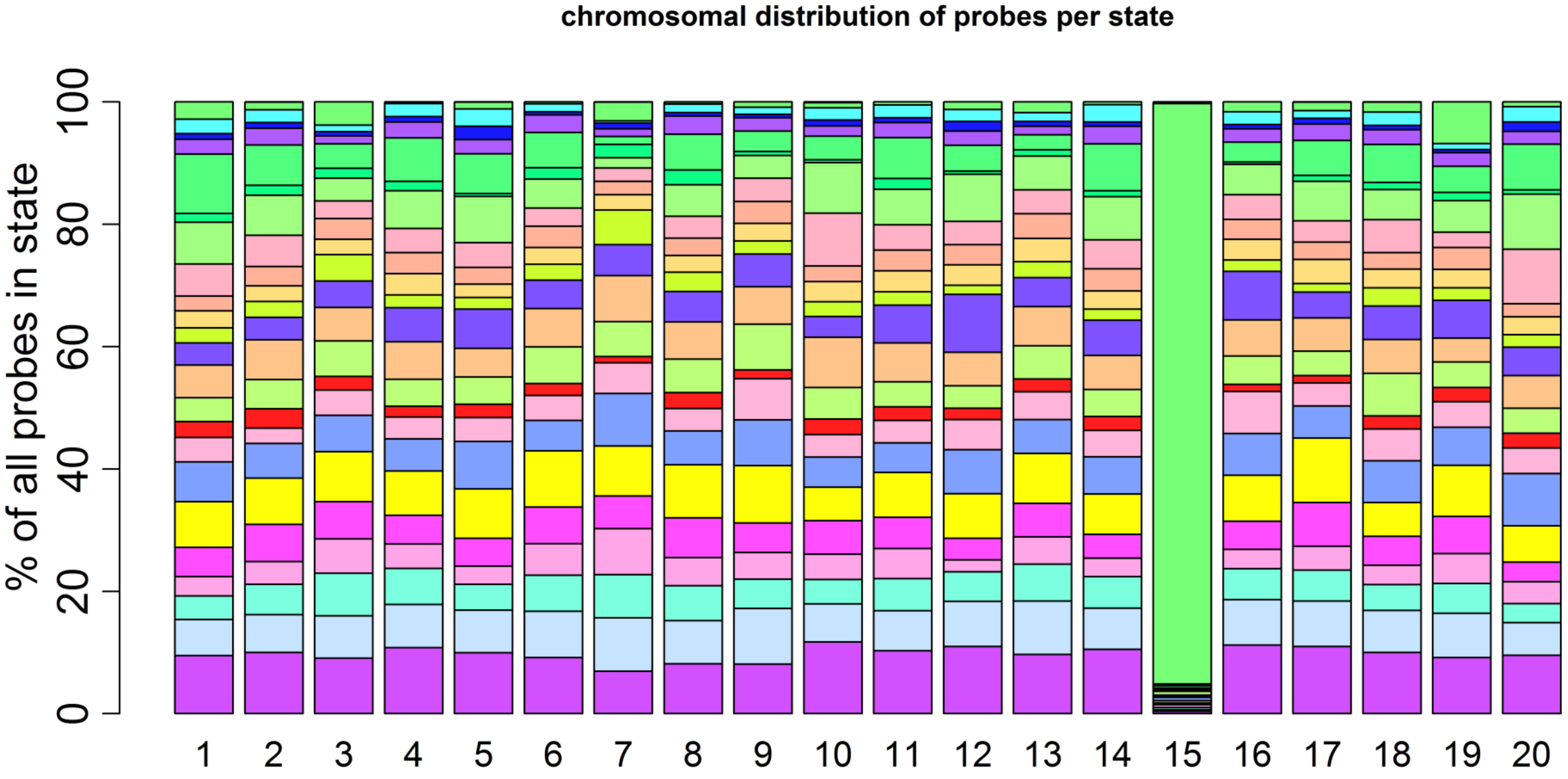

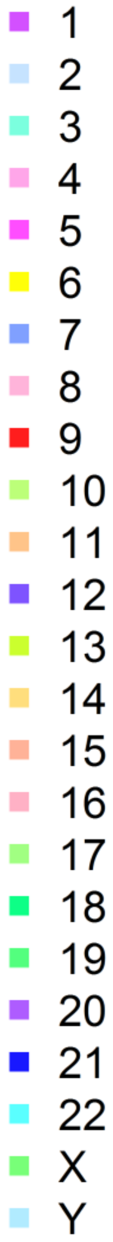

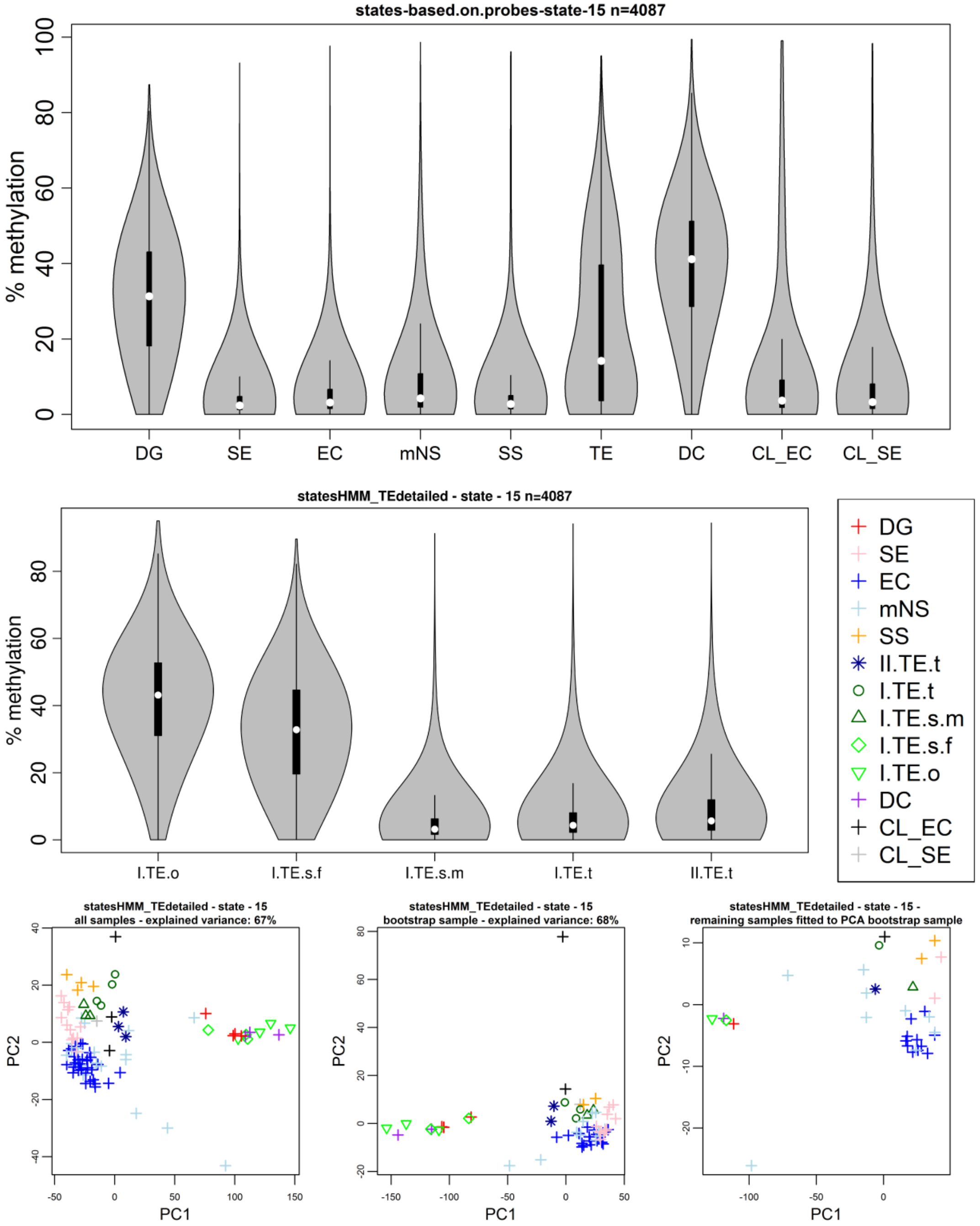

STATE 15

discriminates males from females.

see the legend of Figure 2 for an explanation of the visualizations.
