# Supplementary material for: Genome Wide DNA Methylation Profiles Provide Clues to the Origin and Pathogenesis of Germ Cell Tumors
Source: PLoS One. 2015 Apr 10;10(4):e0122146. doi: 10.1371/journal.pone.0122146 (PMC4479500; doi:10.1371/journal.pone.0122146)

./clusters/cluster10_SE_DGvstype.I.TEhypermethylated_SE_DG_chr17-6898207-6900758

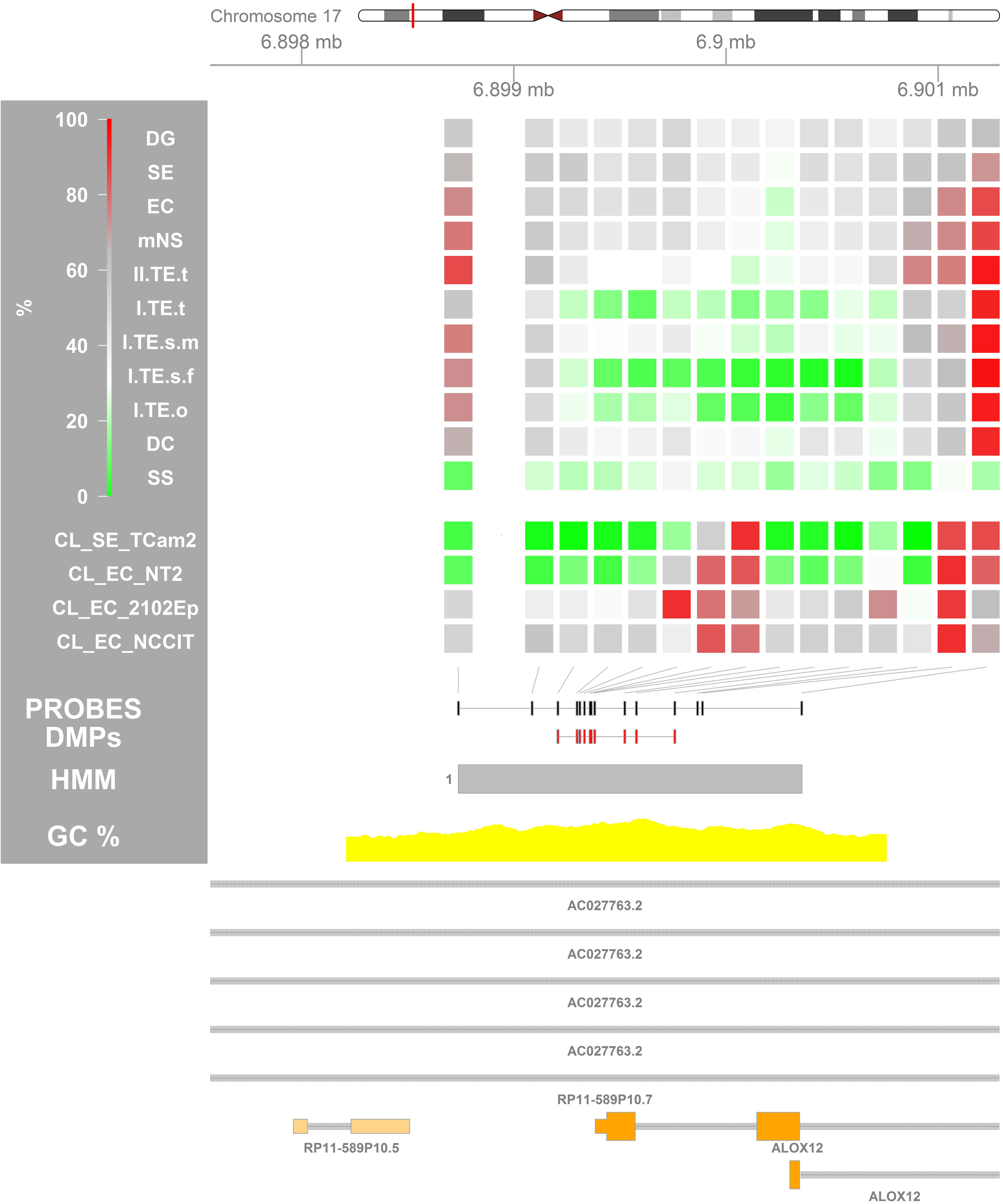

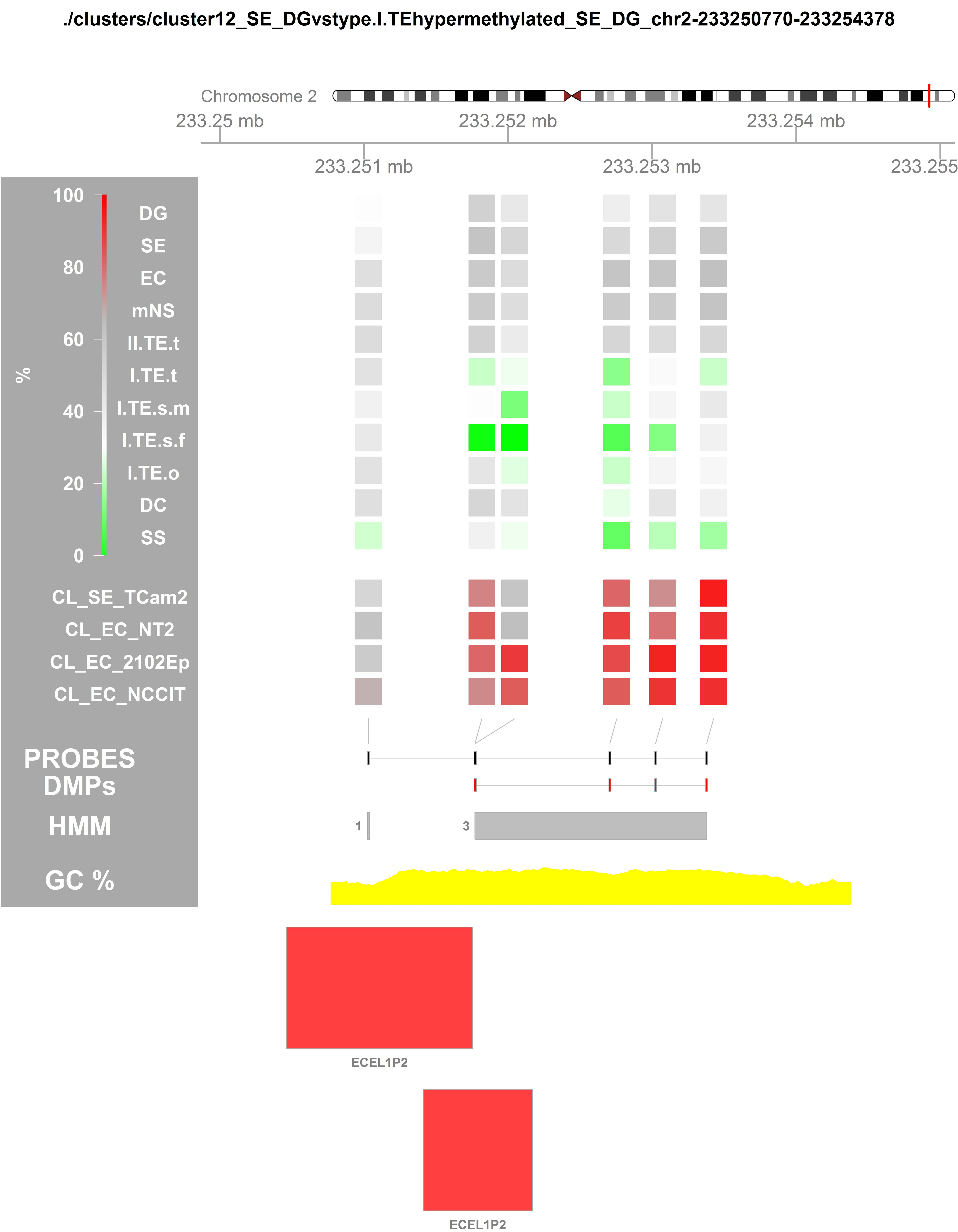
./clusters/cluster12_SE_DGvstype.I.TEhypermethylated_SE_DG_chr2-233250770-233254378
Chromosome 2
233.25 mb
233.252 mb
233.254 mb
233.251 mb
233.253 mb
233.255
100
80
60
40
20
0
%
DG
SE
EC
mNS
II.TE.t
I.TE.t
I.TE.s.m
I.TE.s.f
I.TE.o
DC
SS
CL_SE_TCam2
CL_EC_NT2
CL_EC_2102Ep
CL_EC_NCCIT
PROBES
DMPs
HMM
GC %
1
3
ECEL1P2
ECEL1P2

./clusters/cluster4_SE_DGvstype.I.TEhypermethylated_SE_DG_chr12-125001007-125004558

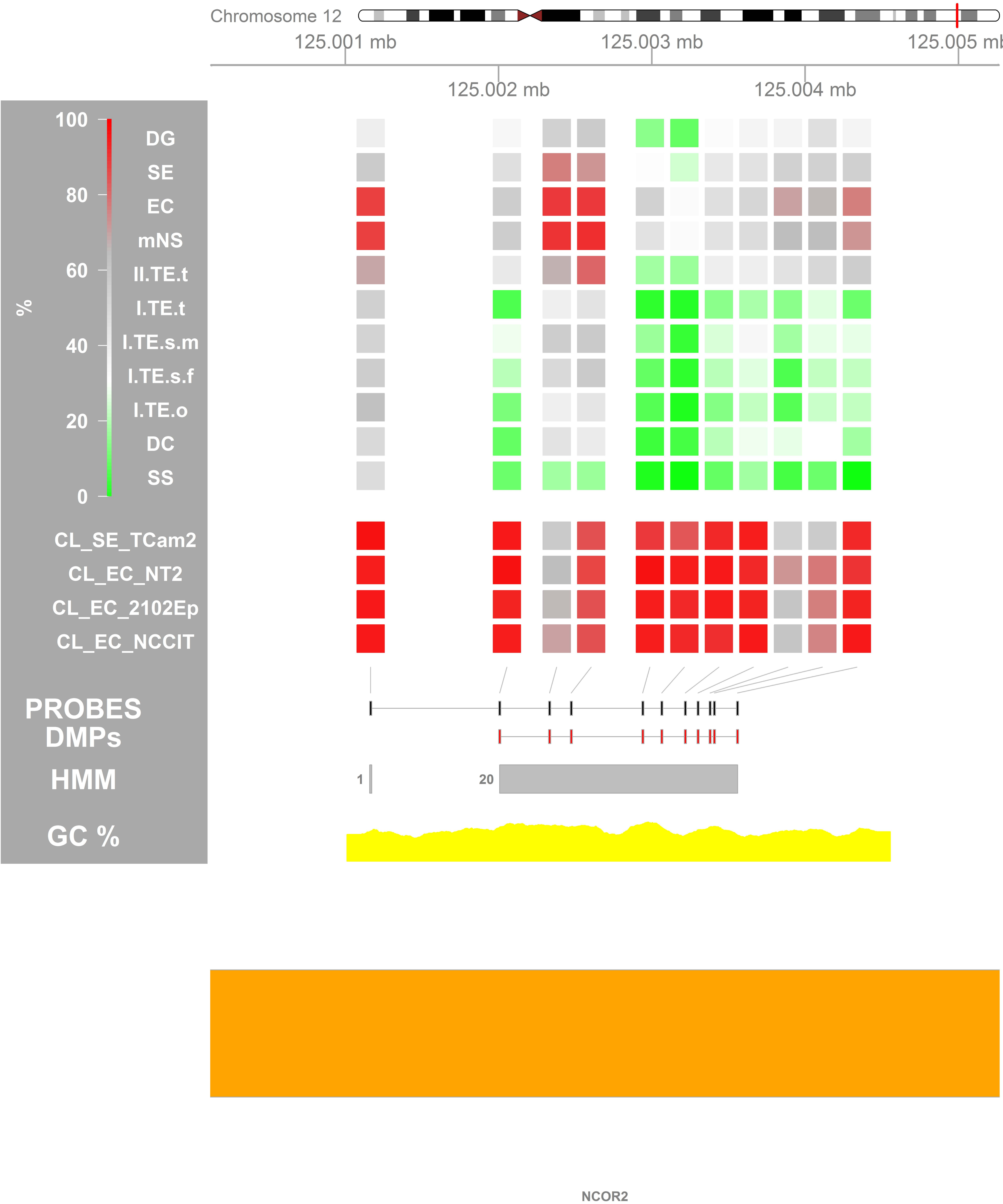

./clusters/cluster2247_EC_mNSvstype.I.TEhypermethylated_EC_mNS_chr22-50584216-50586427

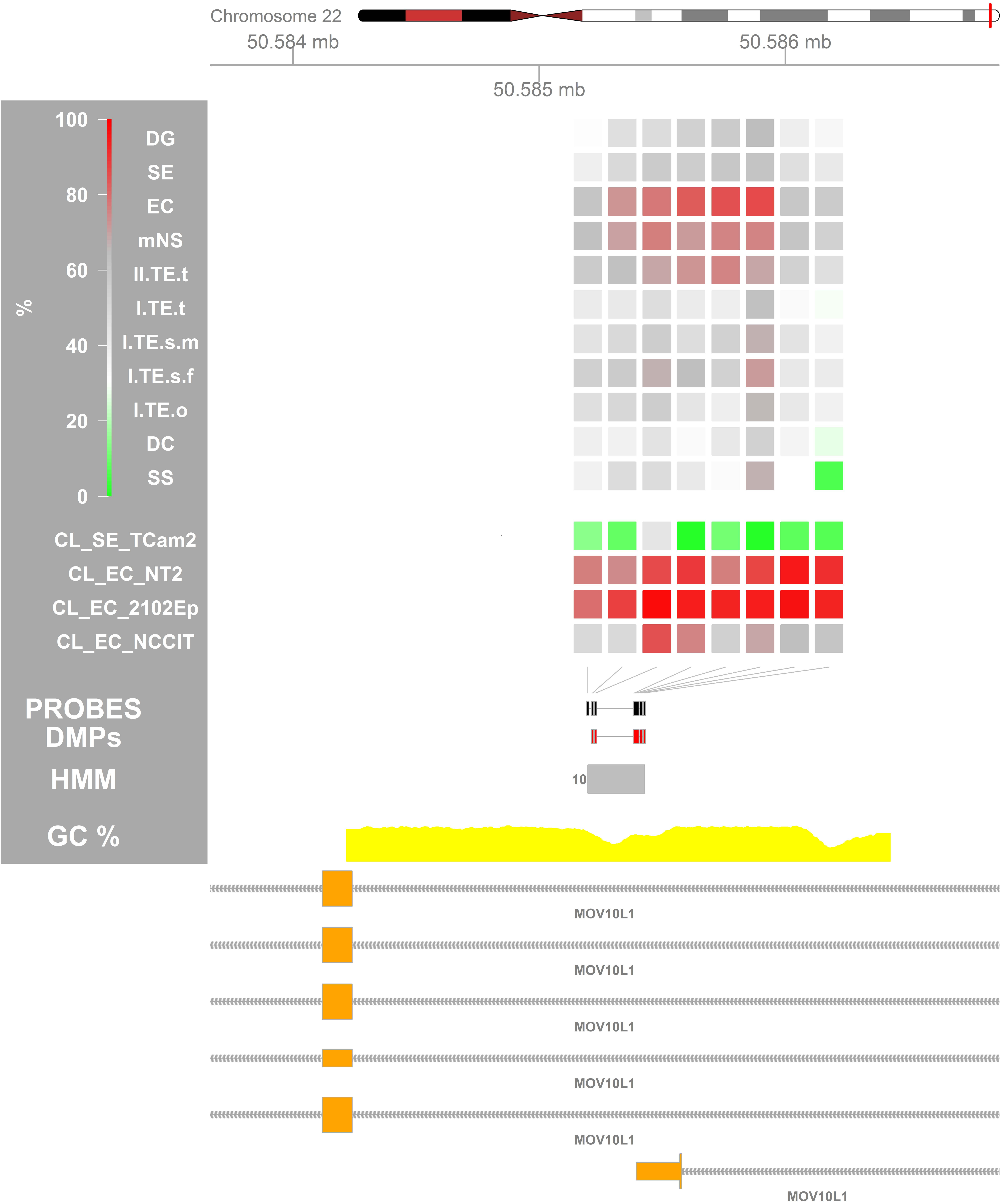

./clusters/cluster272_EC_mNSvstype.I.TEhypermethylated_EC_mNS_chr1-162600334-162603295

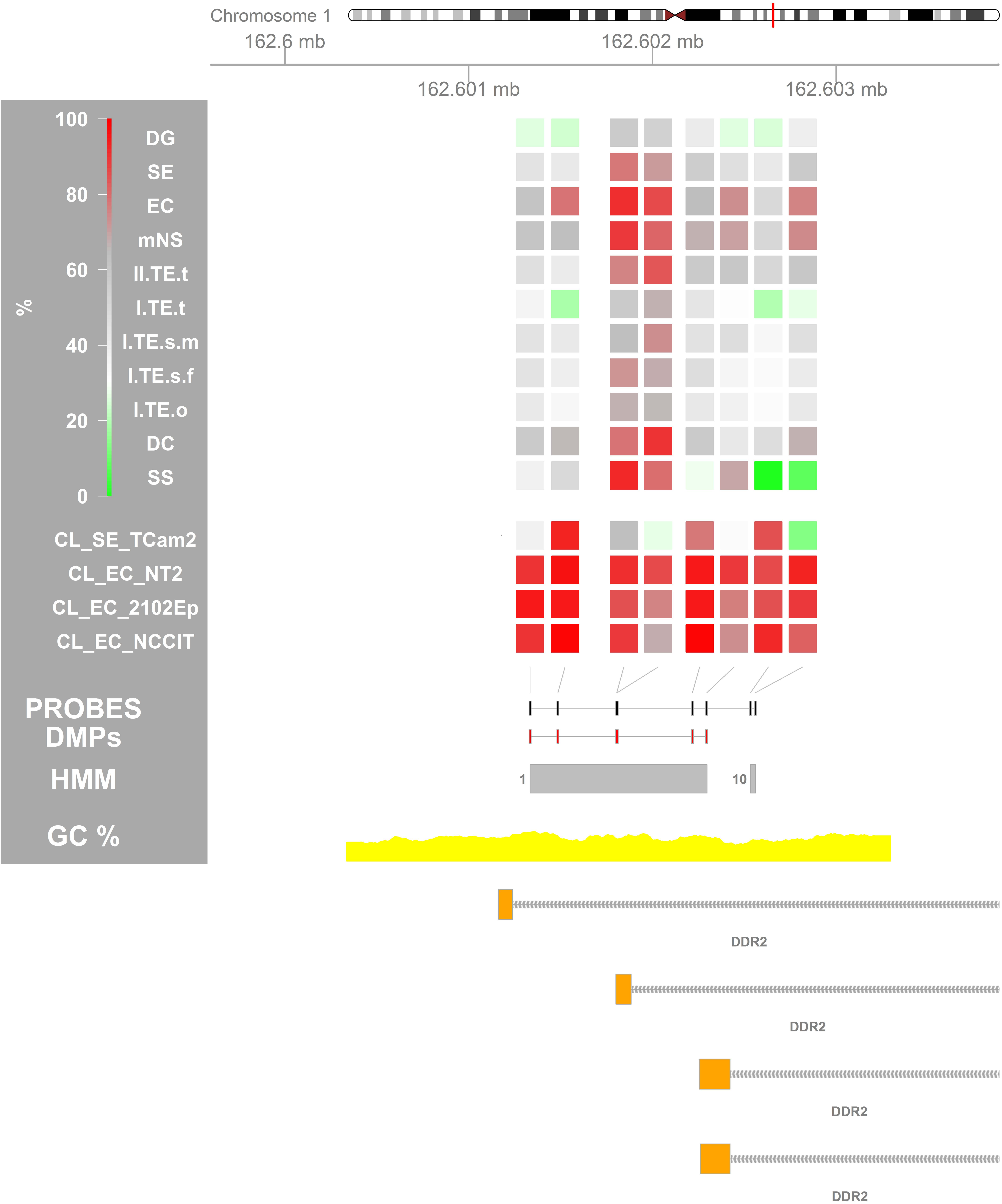

./clusters/cluster30_EC_mNSvstype.I.TEhypermethylated_type.I.TE_chr16-54969523-54974579

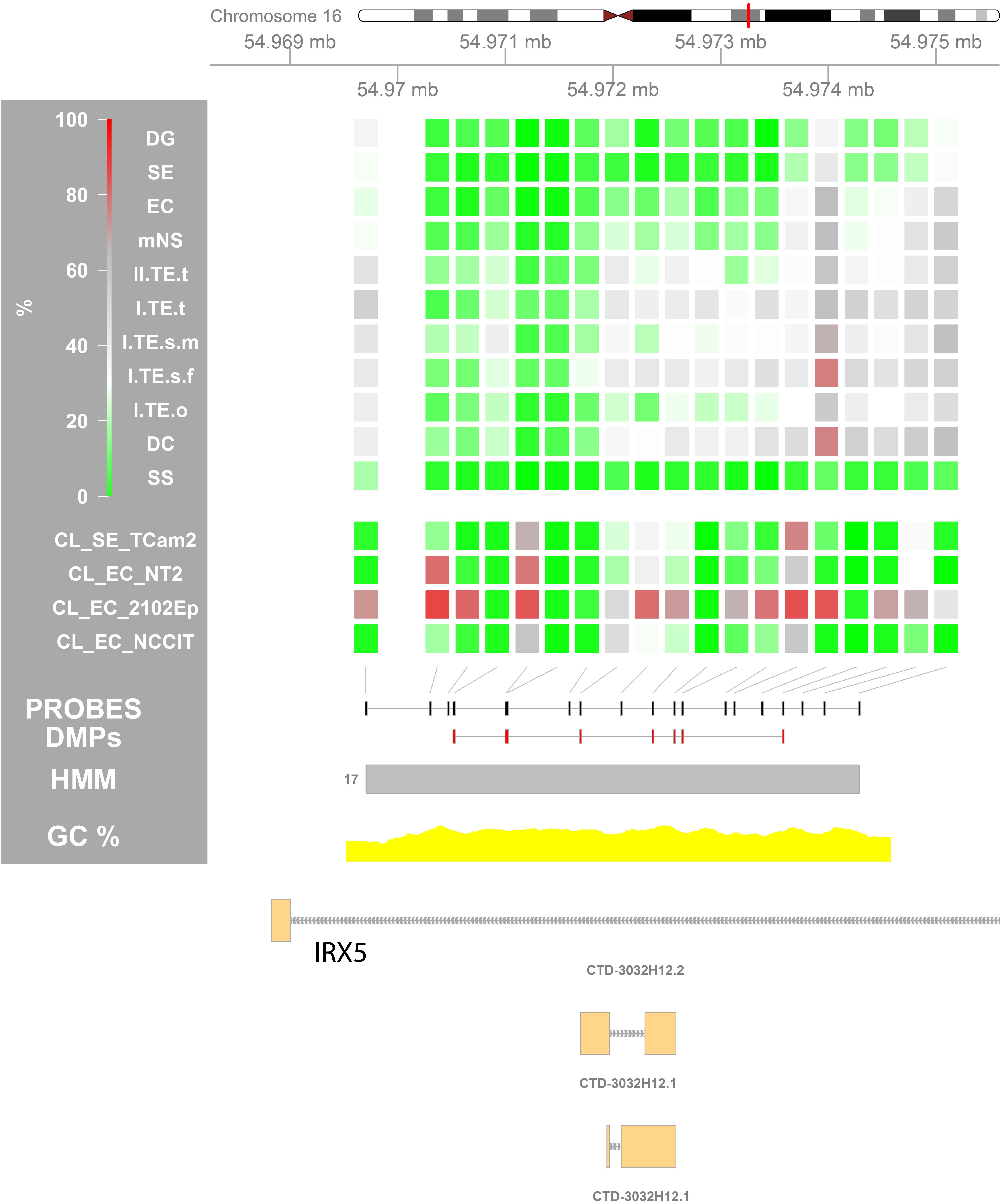

./clusters/cluster3238_EC_mNSvstype.I.TEhypermethylated_EC_mNS_chr9-973533-976997

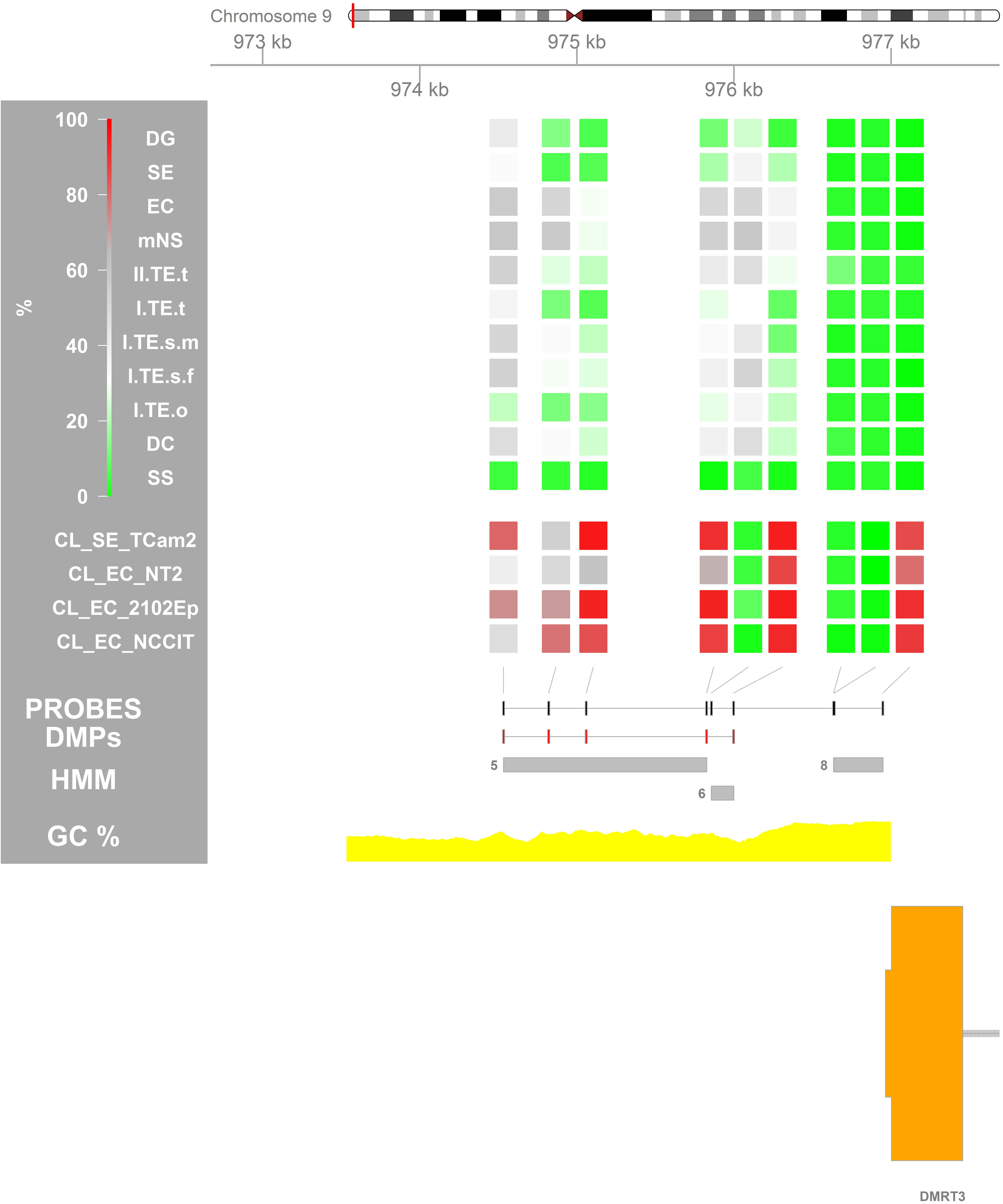

./clusters/cluster51_EC_mNSvstype.I.TEhypermethylated_type.I.TE_chr3-181427280-181429563

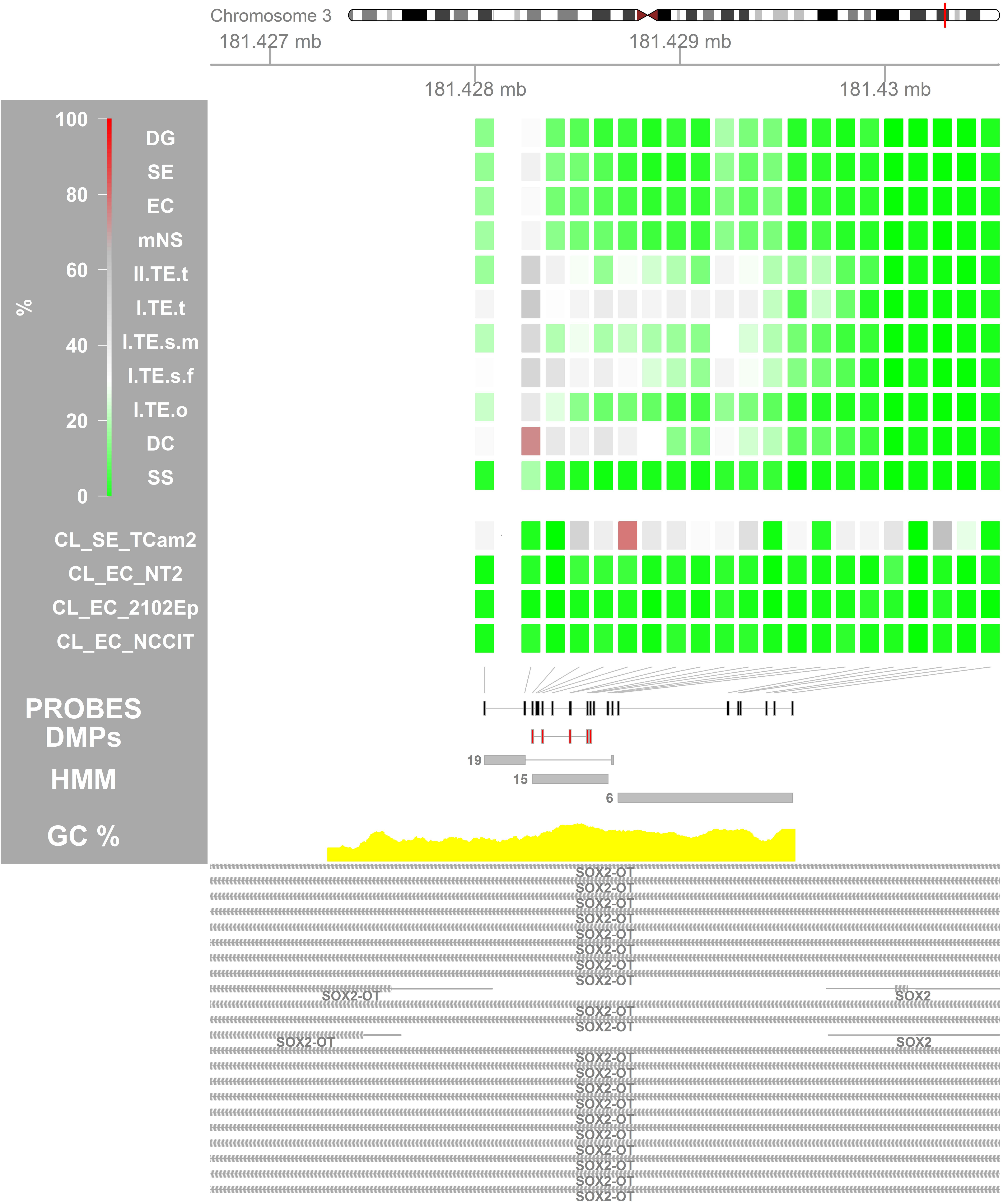

./clusters/cluster53_EC_mNSvstype.I.TEhypermethylated_type.I.TE_chr4-4866102-4869723

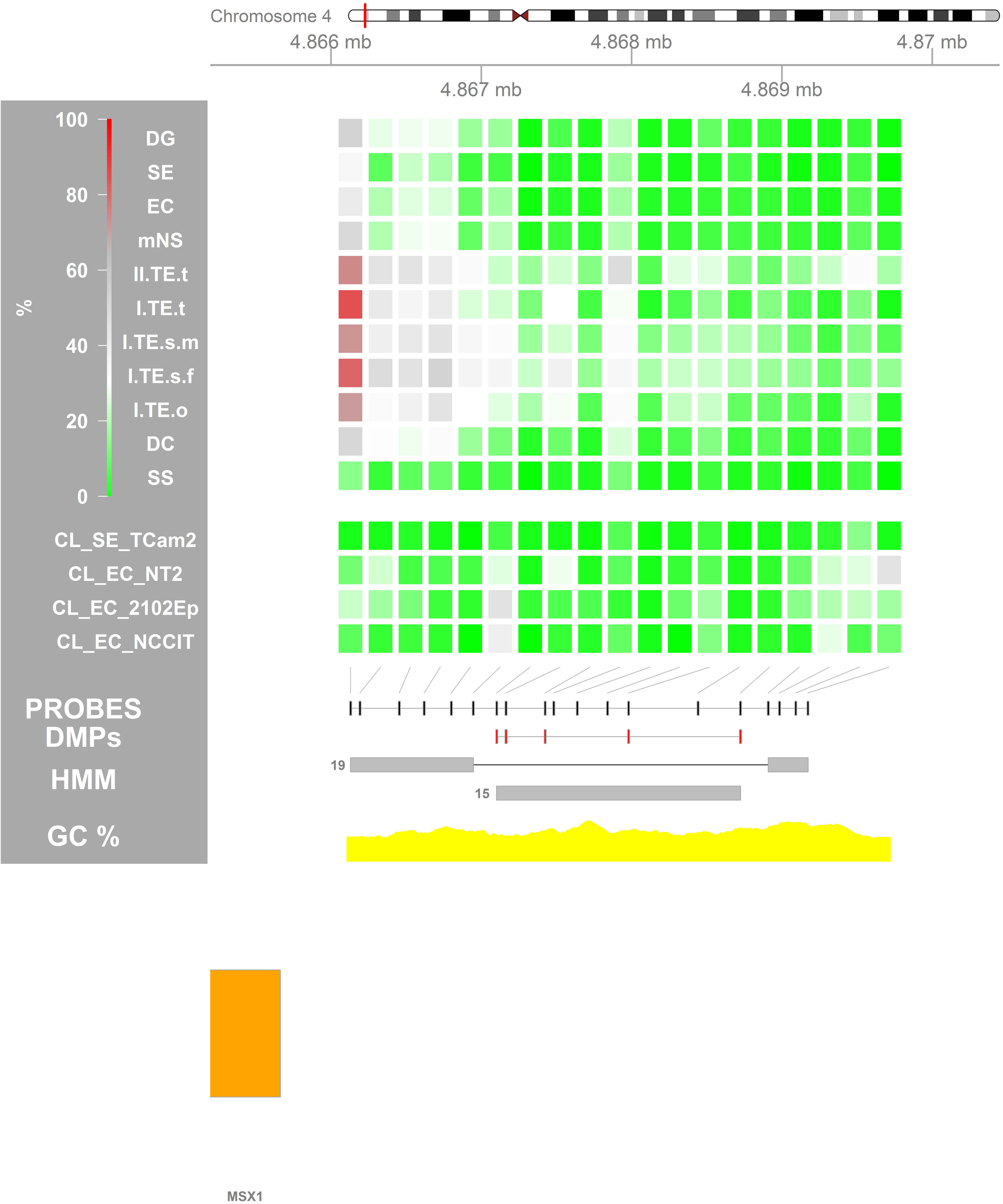

./clusters/cluster605_EC_mNSvstype.I.TEhypermethylated_EC_mNS_chr11-32453718-32456735

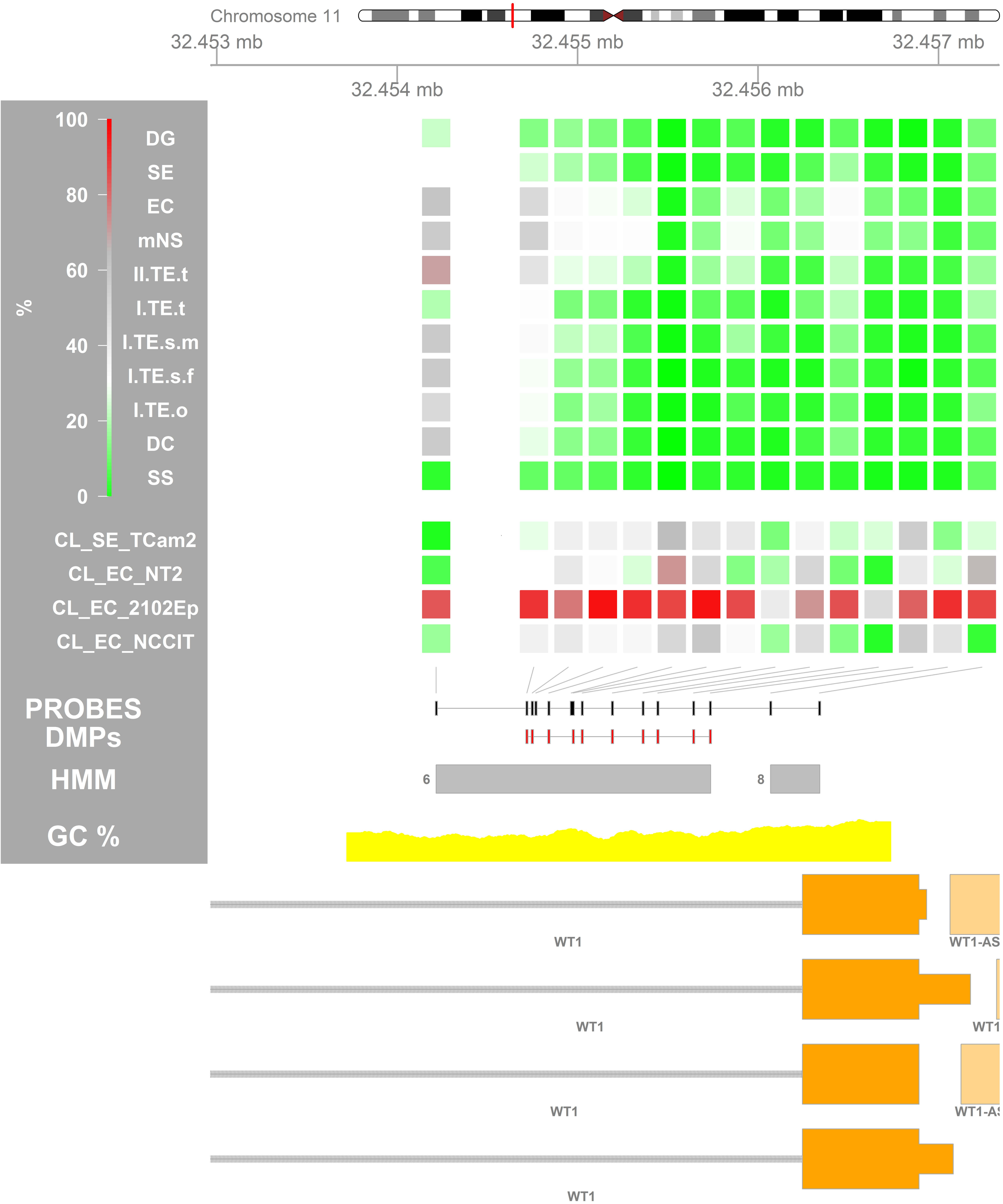

./clusters/cluster1351_SE_DGvsSShypermethylated_SE_DG_chr17-6897738-6901356

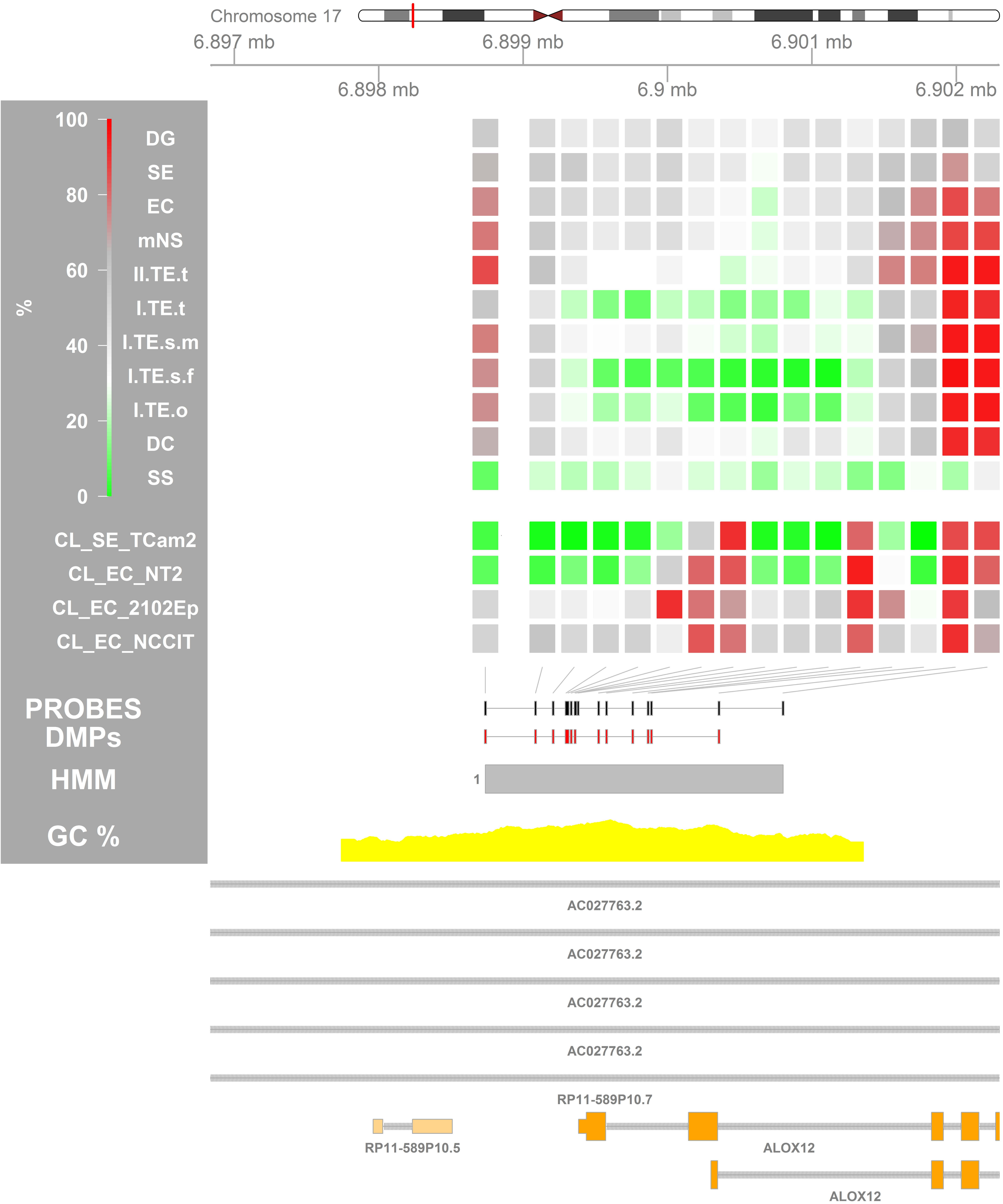

./clusters/cluster2058_SE_DGvsSShypermethylated_SE_DG_chr2-233250770-233254378

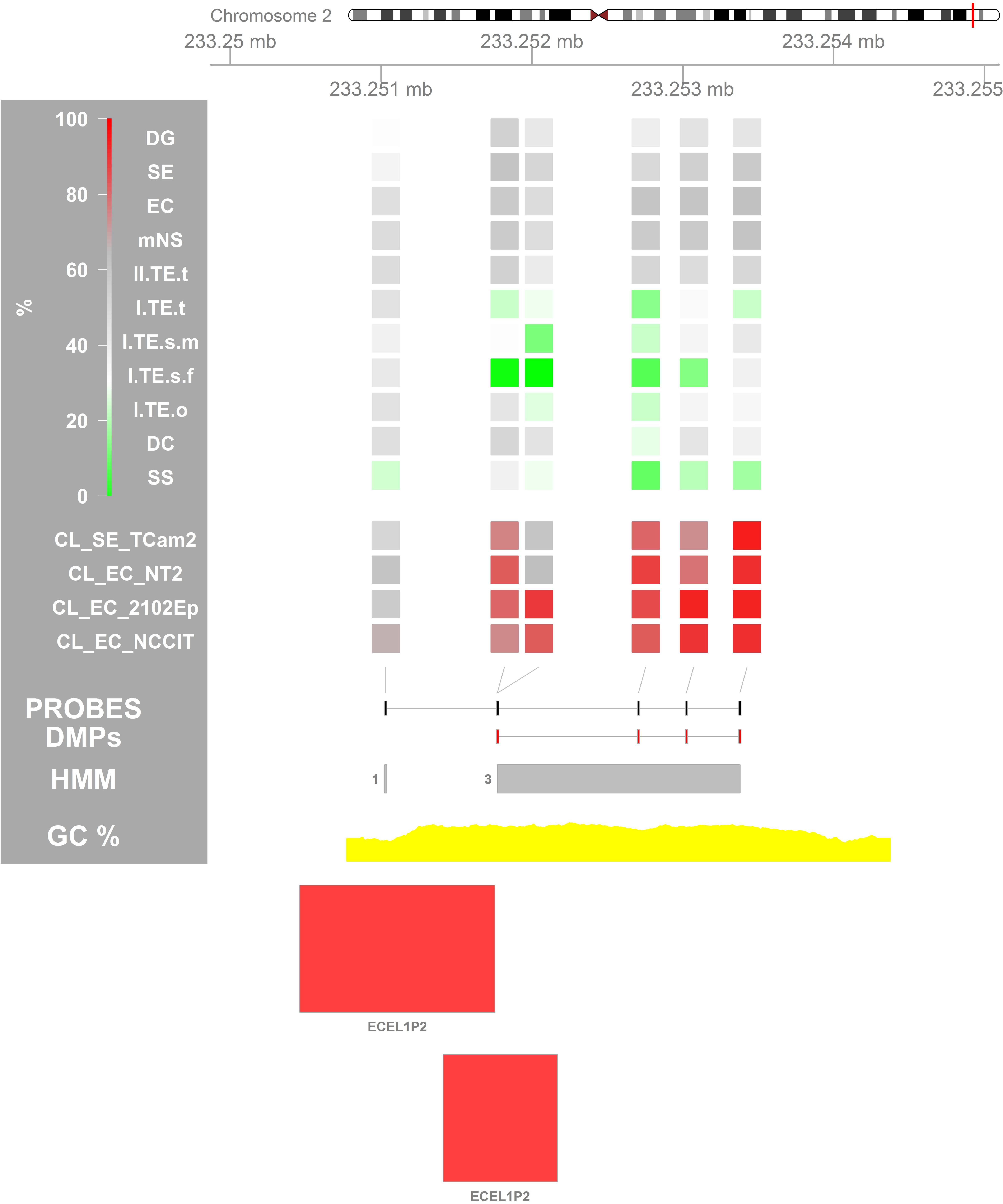

./clusters/cluster236_SE_DGvsSShypermethylated_SS_chr6-29625990-29628845

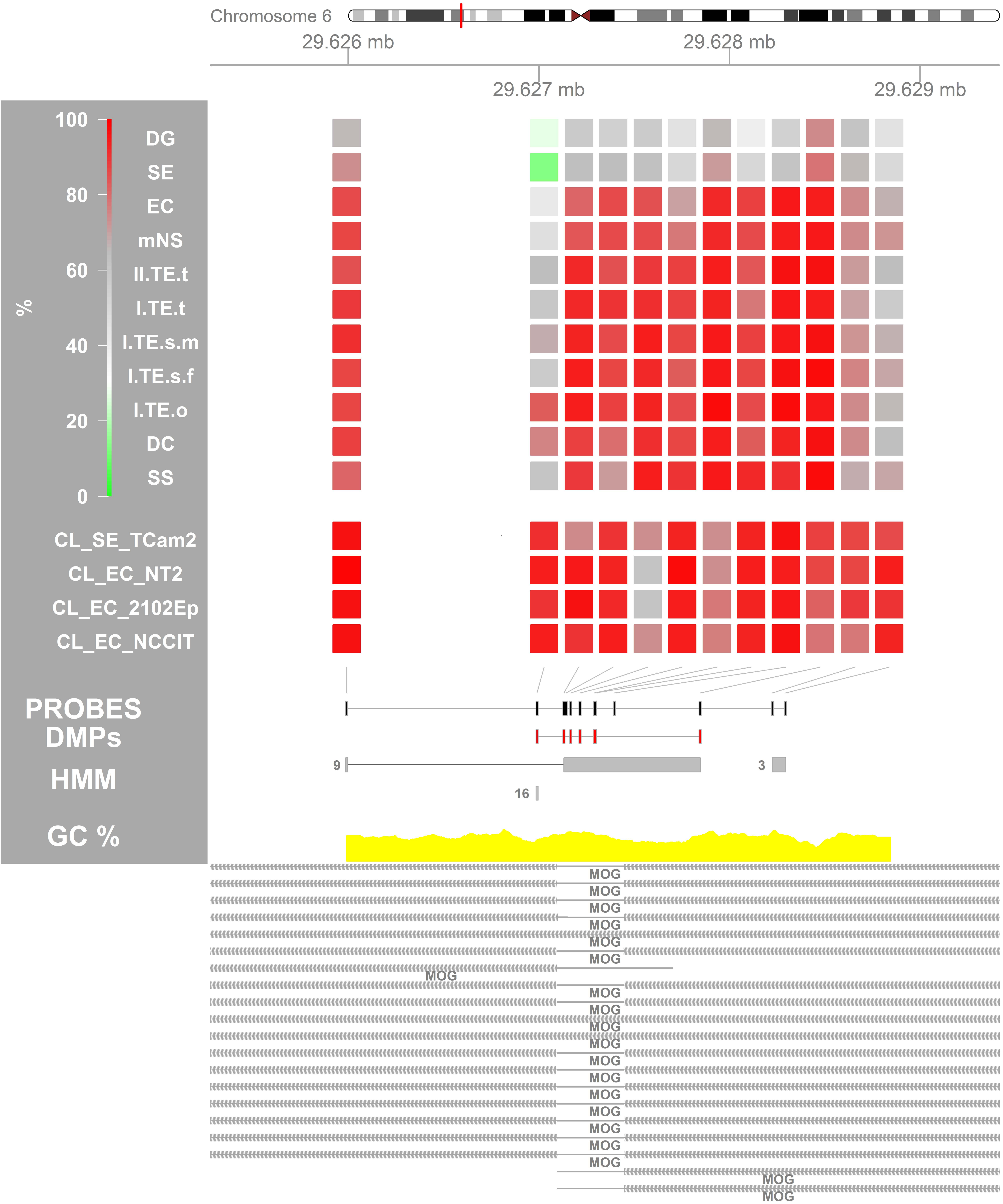

./clusters/cluster2451_SE_DGvsSShypermethylated_SE_DG_chr4-4862874-4865430

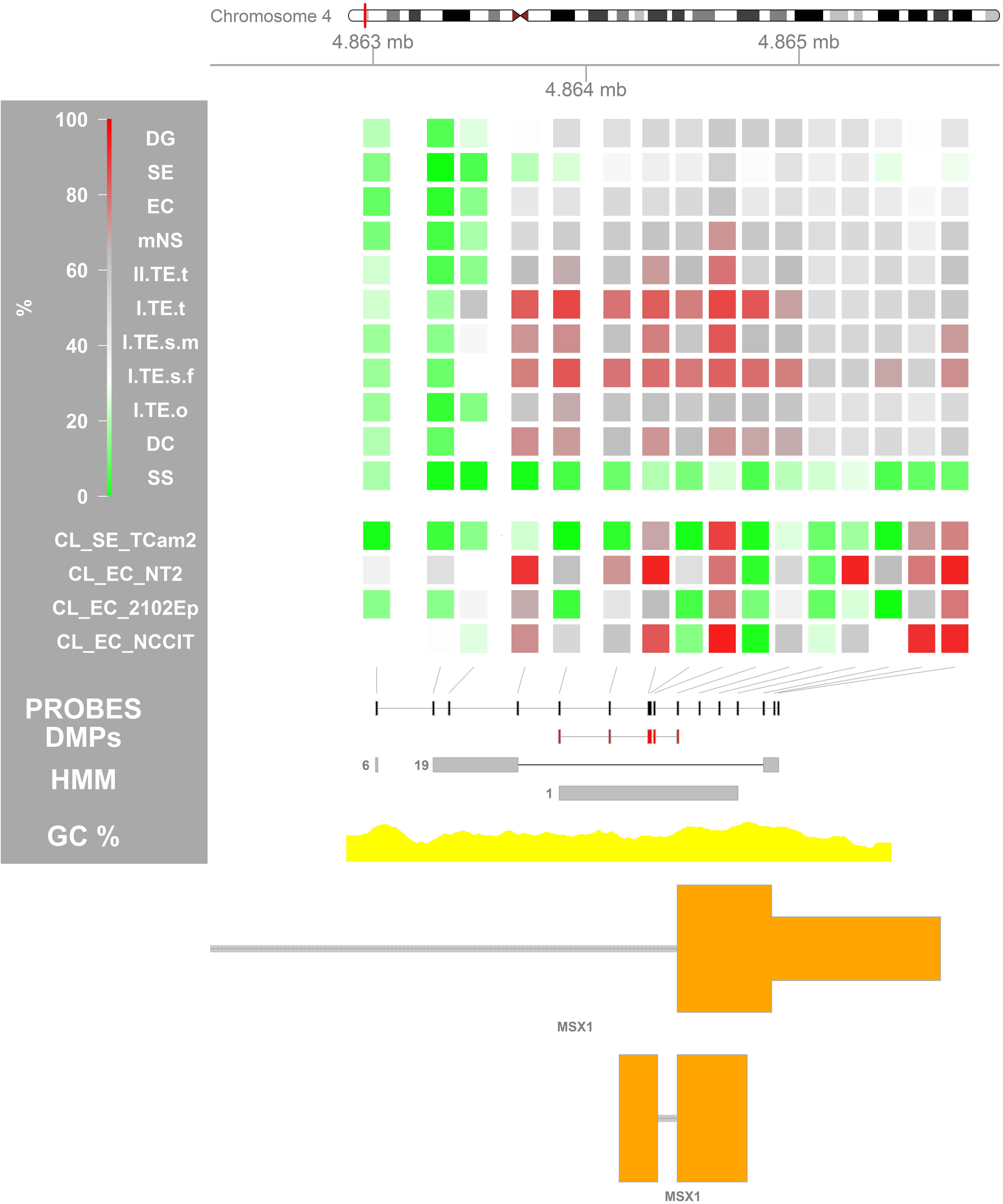

./clusters/cluster287_SE_DGvsSShypermethylated_SS_chr7-100768903-100771476

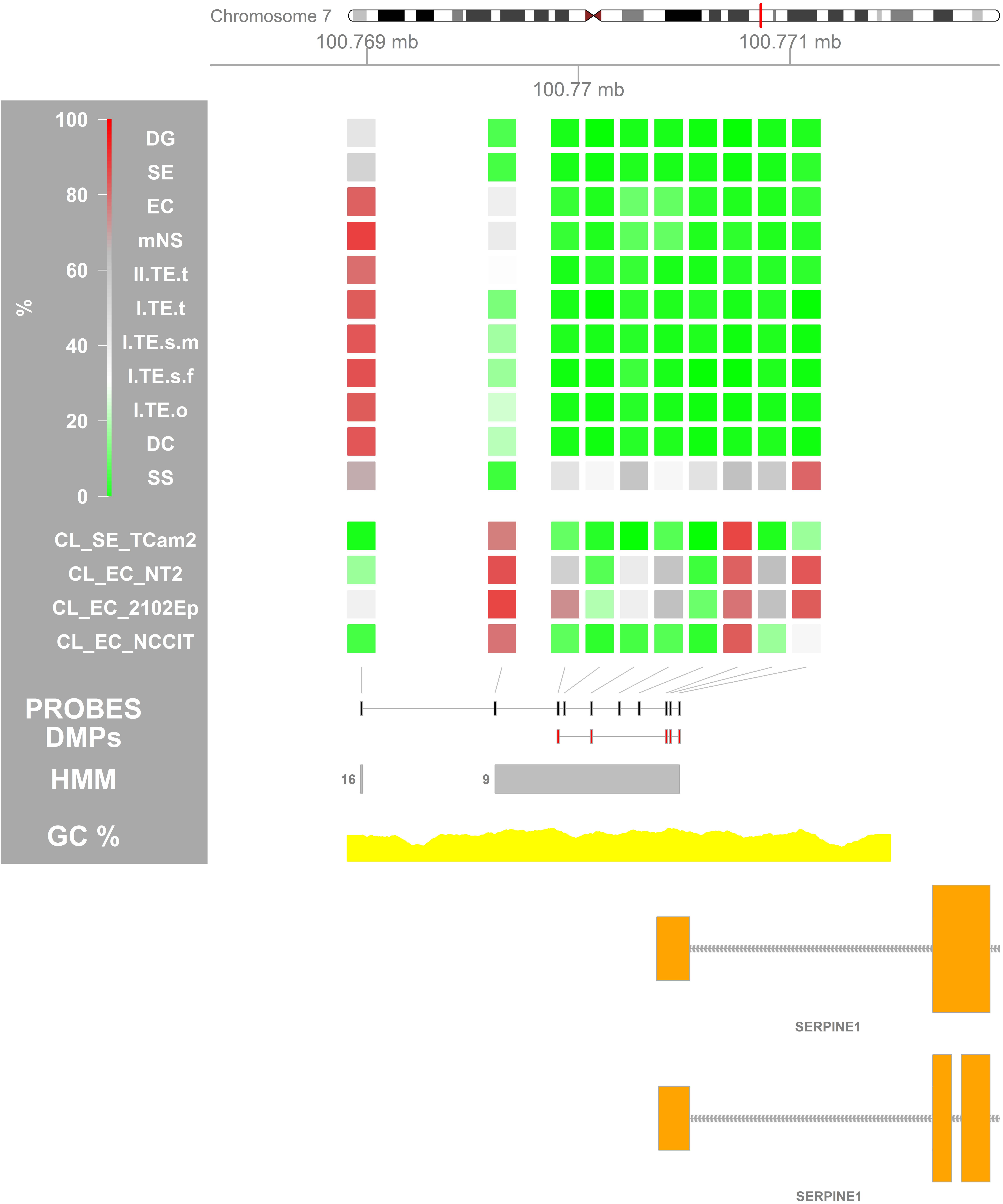

./clusters/cluster769_SE_DGvsSShypermethylated_SE_DG_chr12-124807992-124810934

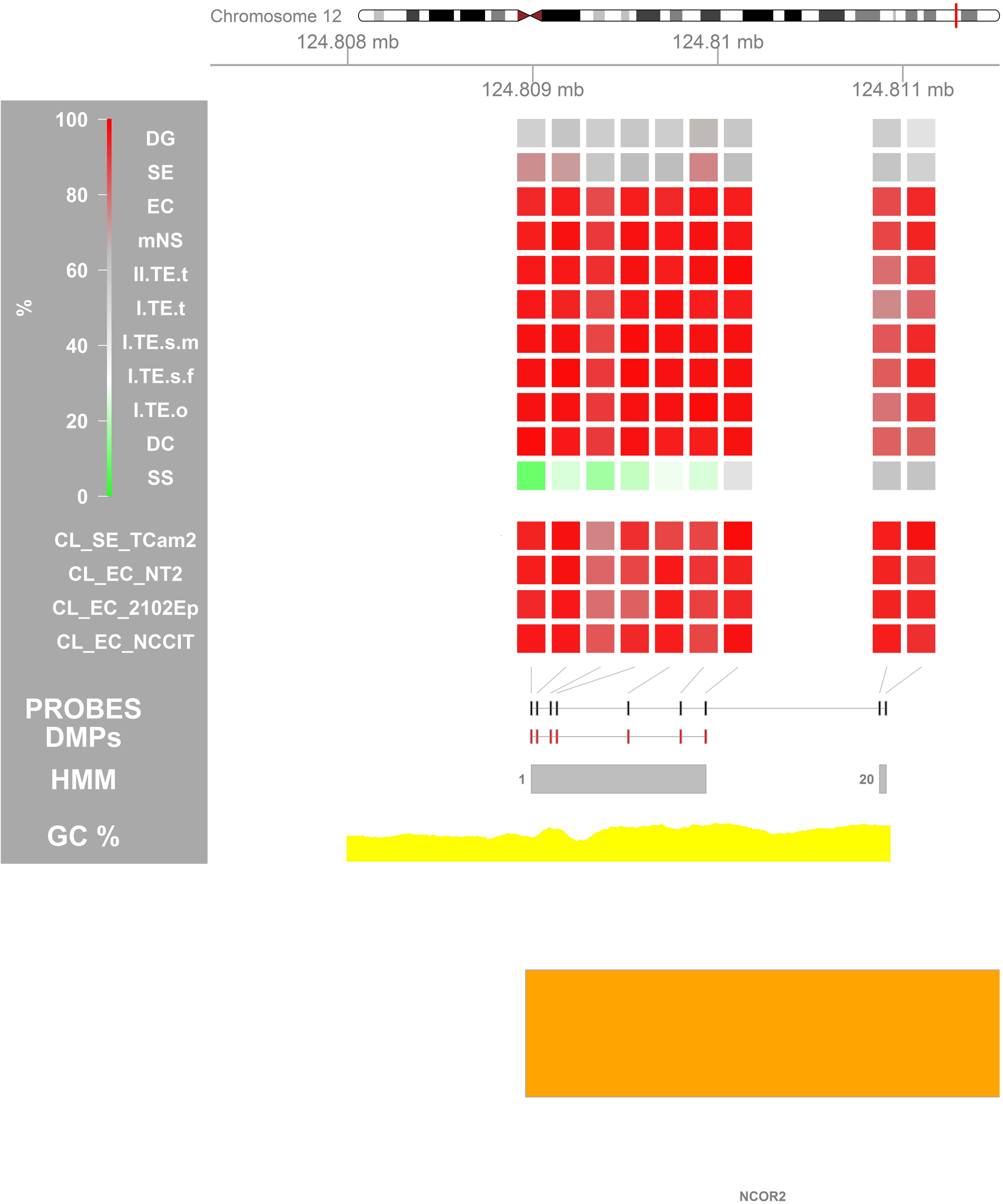

./clusters/cluster881_SE_DGvsSShypermethylated_SE_DG_chr13-110432636-110437780

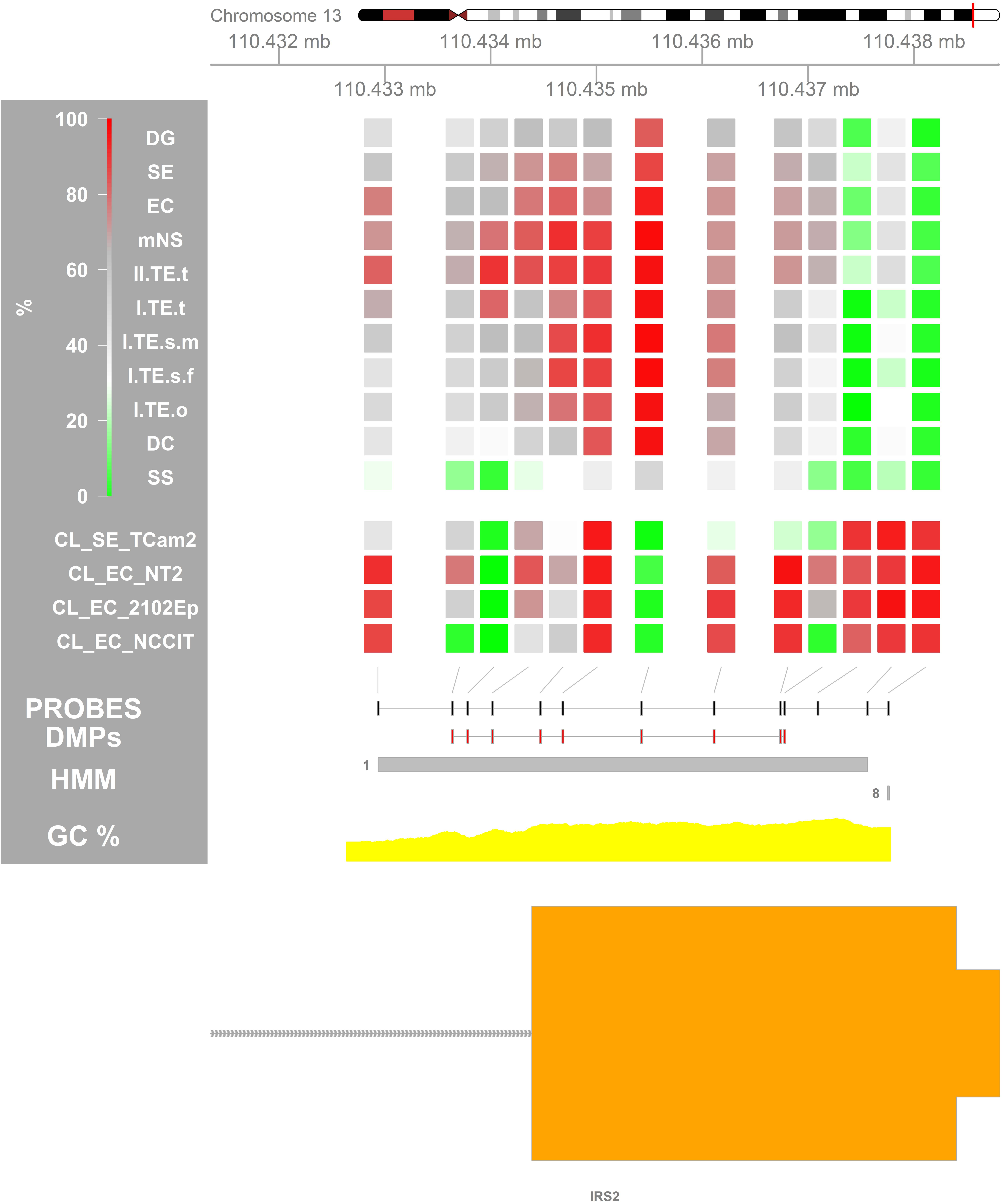

Supplement: S4 Fig — This figure depicts the DMRs between GCT subtypes discussed in the main text in addition to those already visualized in Fig 4. (Visualizations) From top to bottom the following is depicted: (1) Four-color heat map indicating methylation % for each individual probe in the depicted region. For the sample groups specified on the left the median methylation % is shown. (2) Position of all probes in the region of interest (ROI) is annotated as black rectangles. (3) HMM segments are displayed as grey boxes spanning the segment’s width and grouped per state. Numbers indicate the state of each (group of) segment(s). (5) GC% was obtained from the UCSC genome browser database (gc5Base table). (6) Transcripts overlapping with the ROI are plotted at the bottom. Plot generated using the Gviz package. Abbreviations of histological subtypes are explained in Fig 1A. Please note that the TE group is subdivided based on gender and localization: I = type I; II = type II/formally part of the mNS group, s = sacrum, t = testis, o = ovary, m = male, f = female. CL indicates cell lines. S4B Fig, Methylation profile at GCT subtype specific differentially methylated regions (DMRs)—continued—EC/mNS versus type I TE. This figure depicts the DMRs between GCT subtypes discussed in the main text in addition to those already visualized in Fig 4. (Visualizations) From top to bottom the following is depicted: (1) Four-color heat map indicating methylation % for each individual probe in the depicted region. For the sample groups specified on the left the median methylation % is shown. (2) Position of all probes in the region of interest (ROI) is annotated as black rectangles. (3) HMM segments are displayed as grey boxes spanning the segment’s width and grouped per state. Numbers indicate the state of each (group of) segment(s). (5) GC% was obtained from the UCSC genome browser database (gc5Base table). (6) Transcripts overlapping with the ROI are plotted at the bottom. Plot generated using the Gviz p [file pone.0122146.s004.pdf]
