# Supplementary material for: Genome Wide DNA Methylation Profiles Provide Clues to the Origin and Pathogenesis of Germ Cell Tumors
Source: PLoS One. 2015 Apr 10;10(4):e0122146. doi: 10.1371/journal.pone.0122146 (PMC4479500; doi:10.1371/journal.pone.0122146)
Supplement: S6 Fig — ICRs identified as described in the materials and methods sections were checked for coverage on the 450K array. 21/28 unique ICRs were covered by one or more probes. These were visualized here (overview: S4 Table). H19_IGF2 regions: the overlapping transcript is an aberrant, long alternative transcript (H19-012, ENST00000428066). These ICRs regulates H19 and IGF2 expression and lie upstream all other transcripts of H19. (Visualizations) From top to bottom the following is depicted: (1) Four-color heat map indicating methylation % for each individual probe in the depicted region. For the sample groups specified on the left the median methylation % is shown. (2) Position of all probes in the region of interest (ROI) is annotated as black rectangles. (3) HMM segments are displayed as grey boxes spanning the segment’s width and grouped per state. Numbers indicate the state of each (group of) segment(s). (5) GC% was obtained from the UCSC genome browser database (gc5Base table). (6) Transcripts overlapping with the ROI are plotted at the bottom. Plot generated using the Gviz package. Abbreviations of histological subtypes are explained in Fig 1A. Please note that the TE group is subdivided based on gender and localization: I = type I; II = type II/formally part of the mNS group, s = sacrum, t = testis, o = ovary, m = male, f = female. CL indicates cell lines. (PDF) [file pone.0122146.s006.pdf]

GRB10, GB10 - Paternal_chr7-50849753-50850871

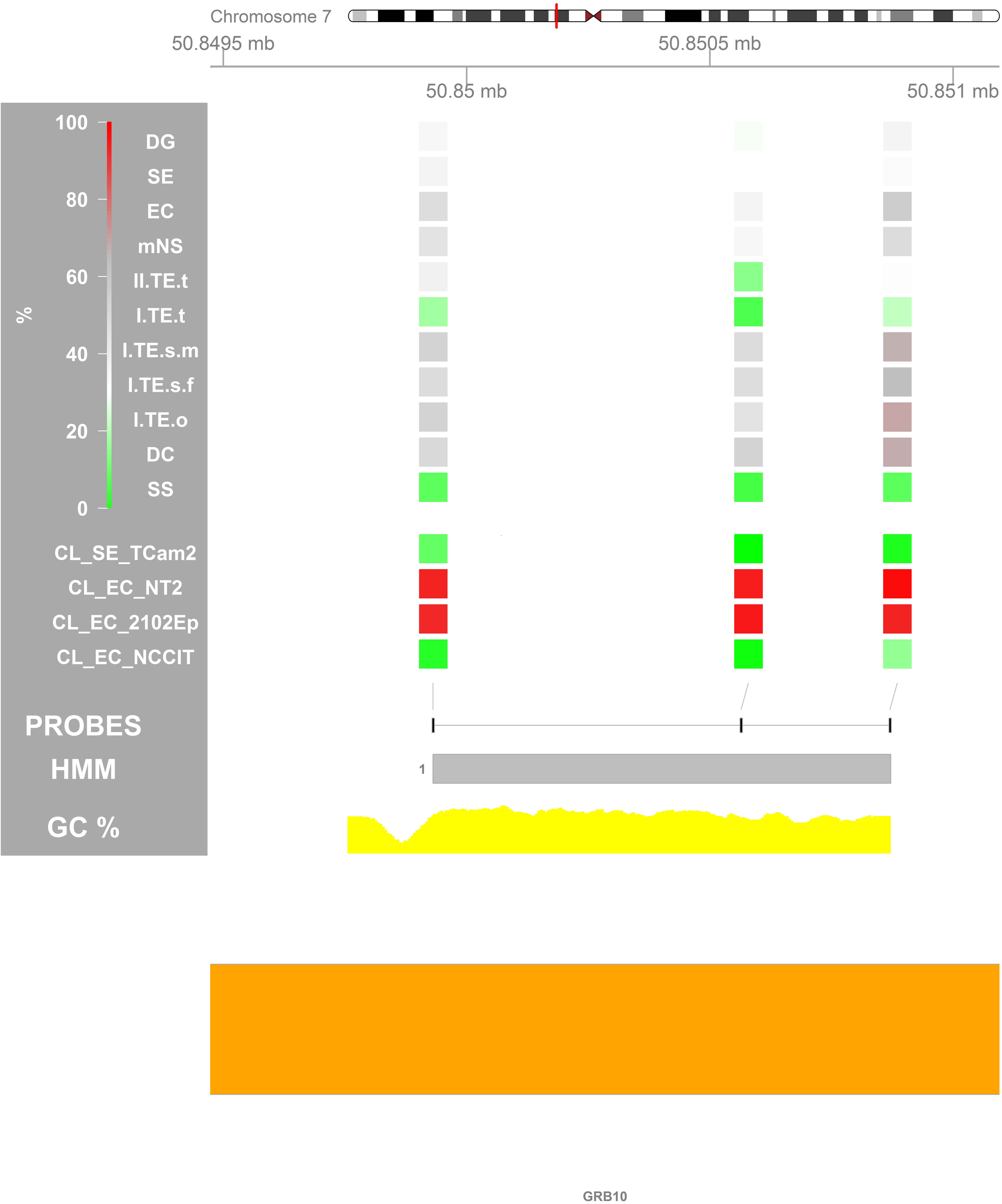

H19_IGF2 - Maternal_chr11-2019368-2019728

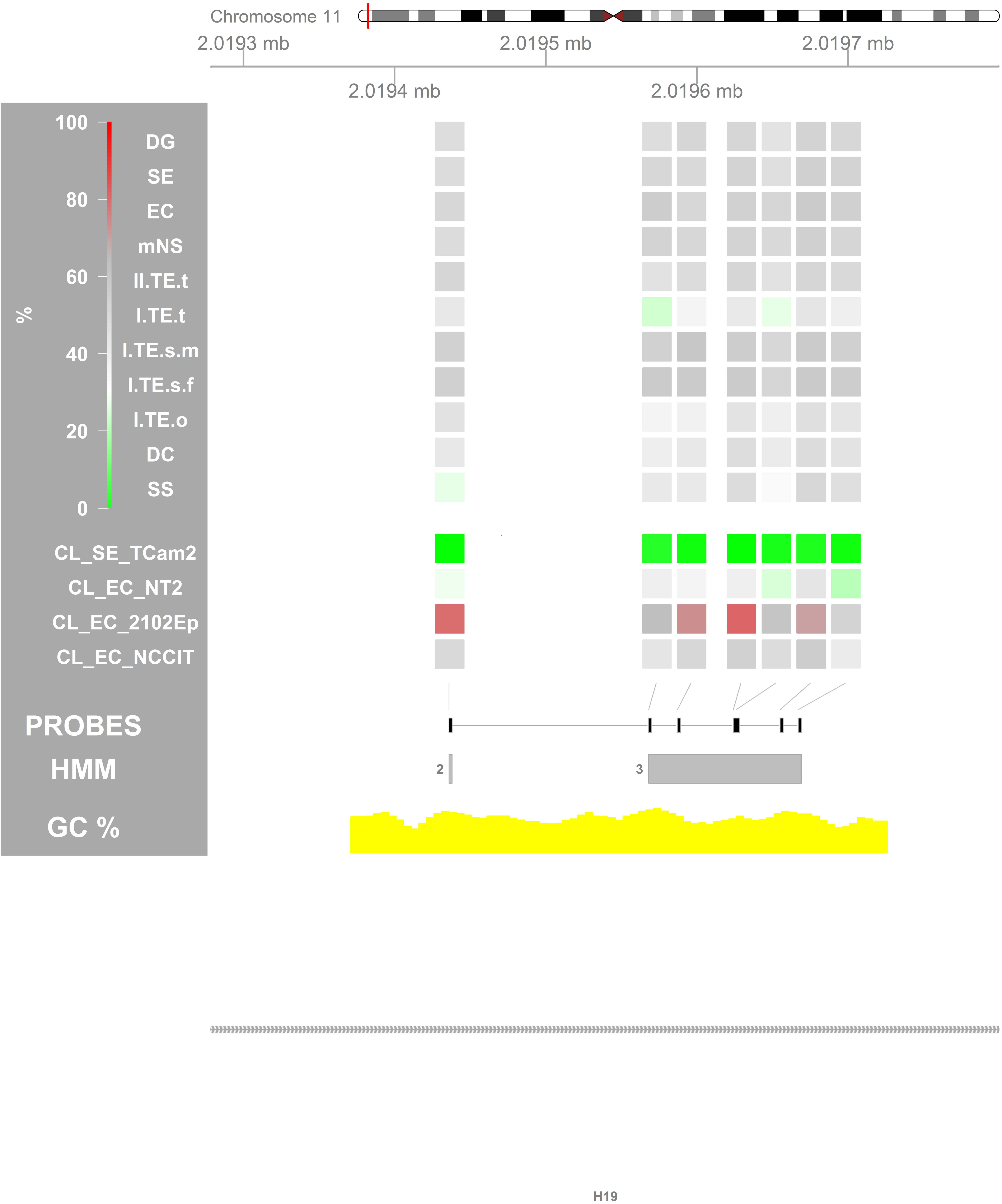

H19_IGF2, IGF_H19.ICR - Maternal_chr11-2020834-2023499

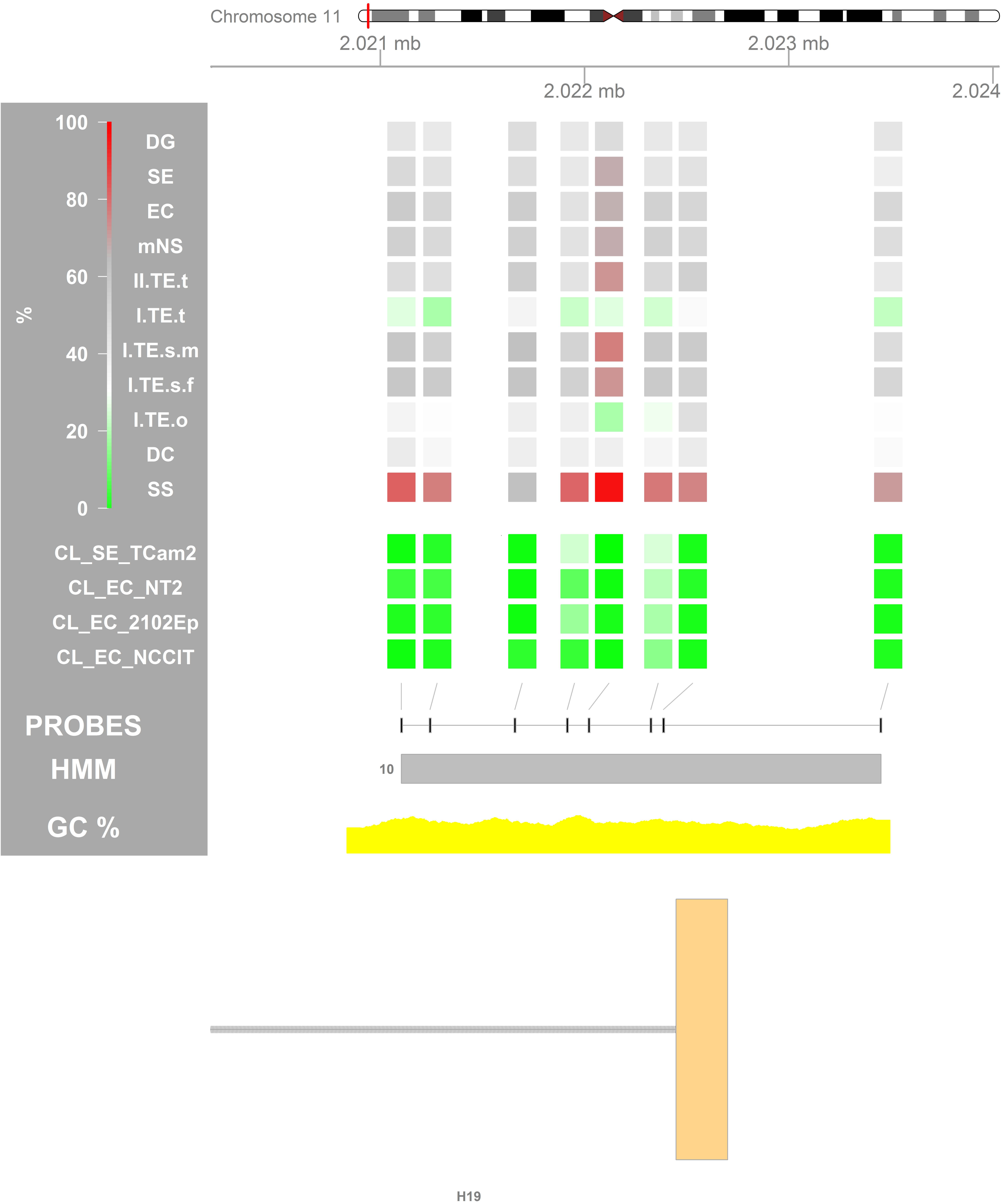

IGF2.DMR2 - Paternal_chr11-2154283-2154956

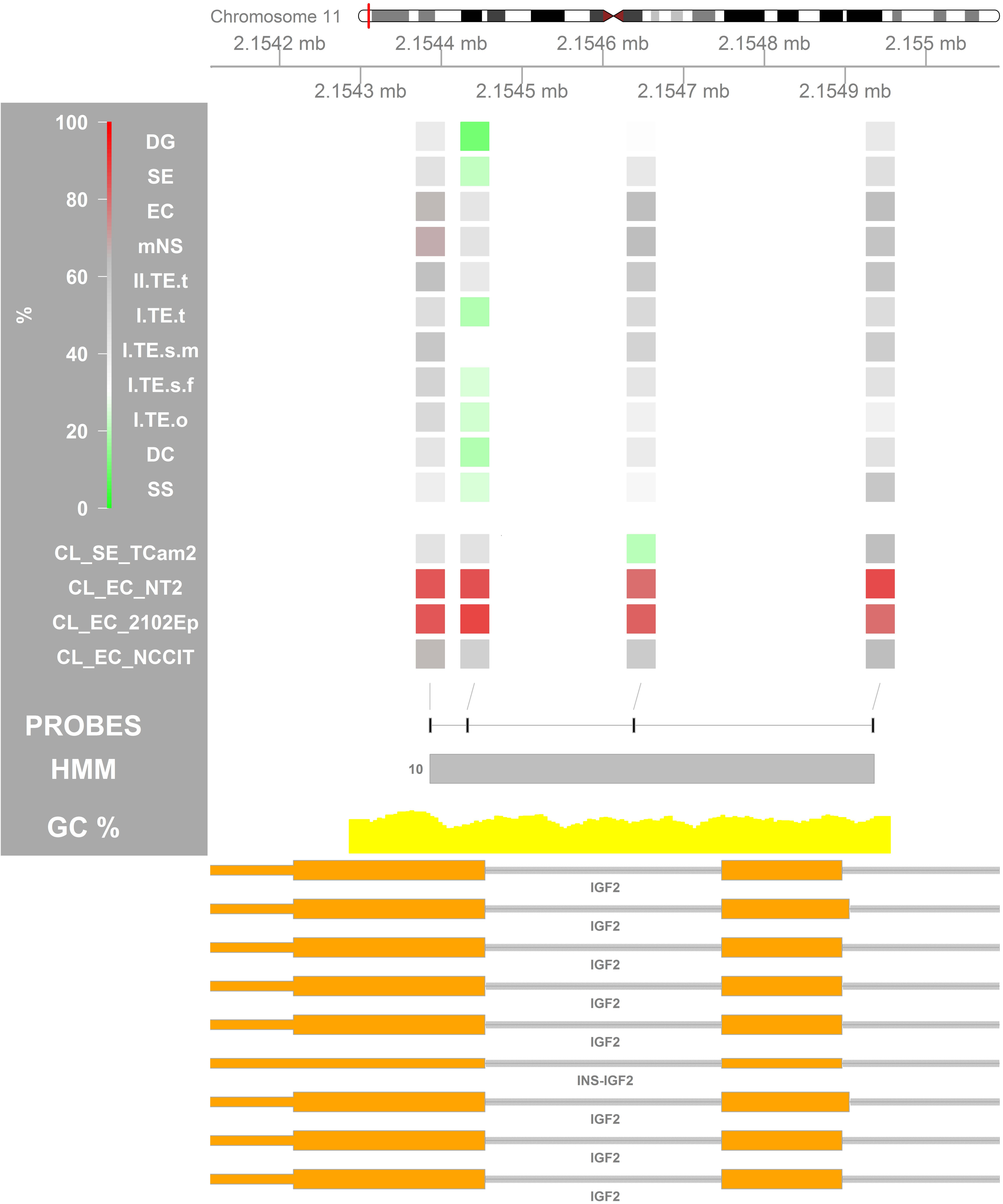

INPP5F_V2 - Paternal_chr10-121577530-121578385

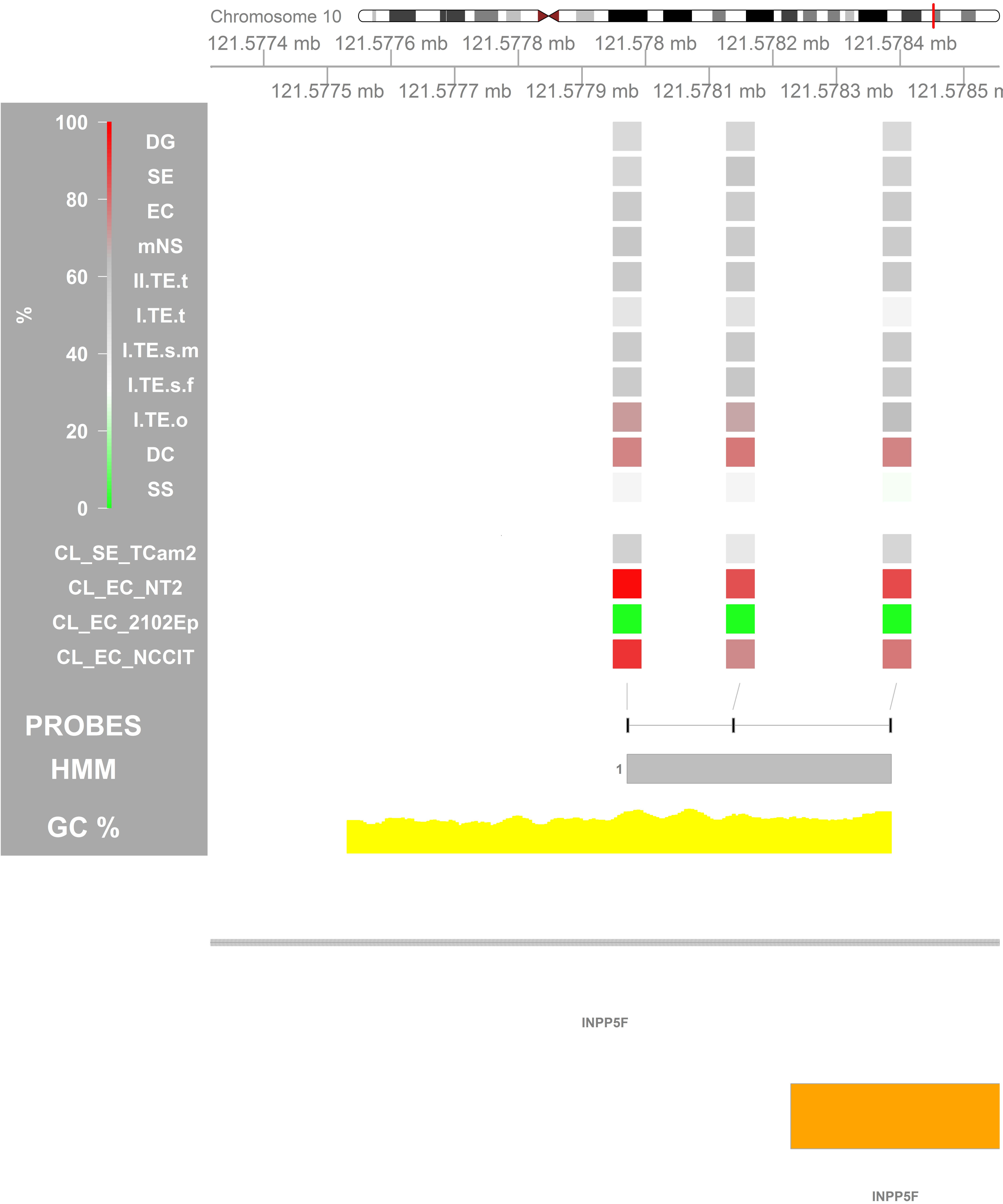

KCNQ1OT1 - Paternal_chr11-2720535-2722187

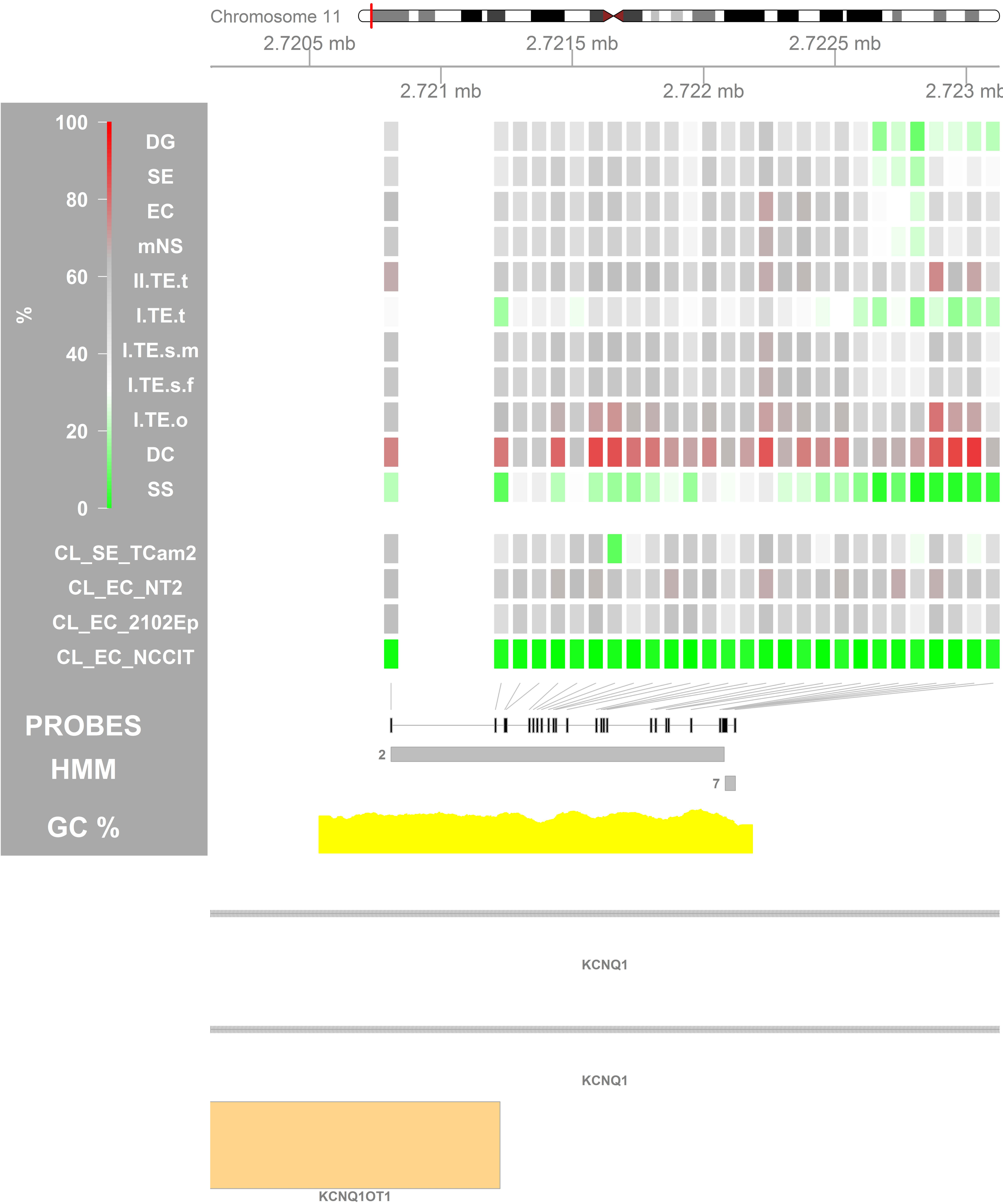

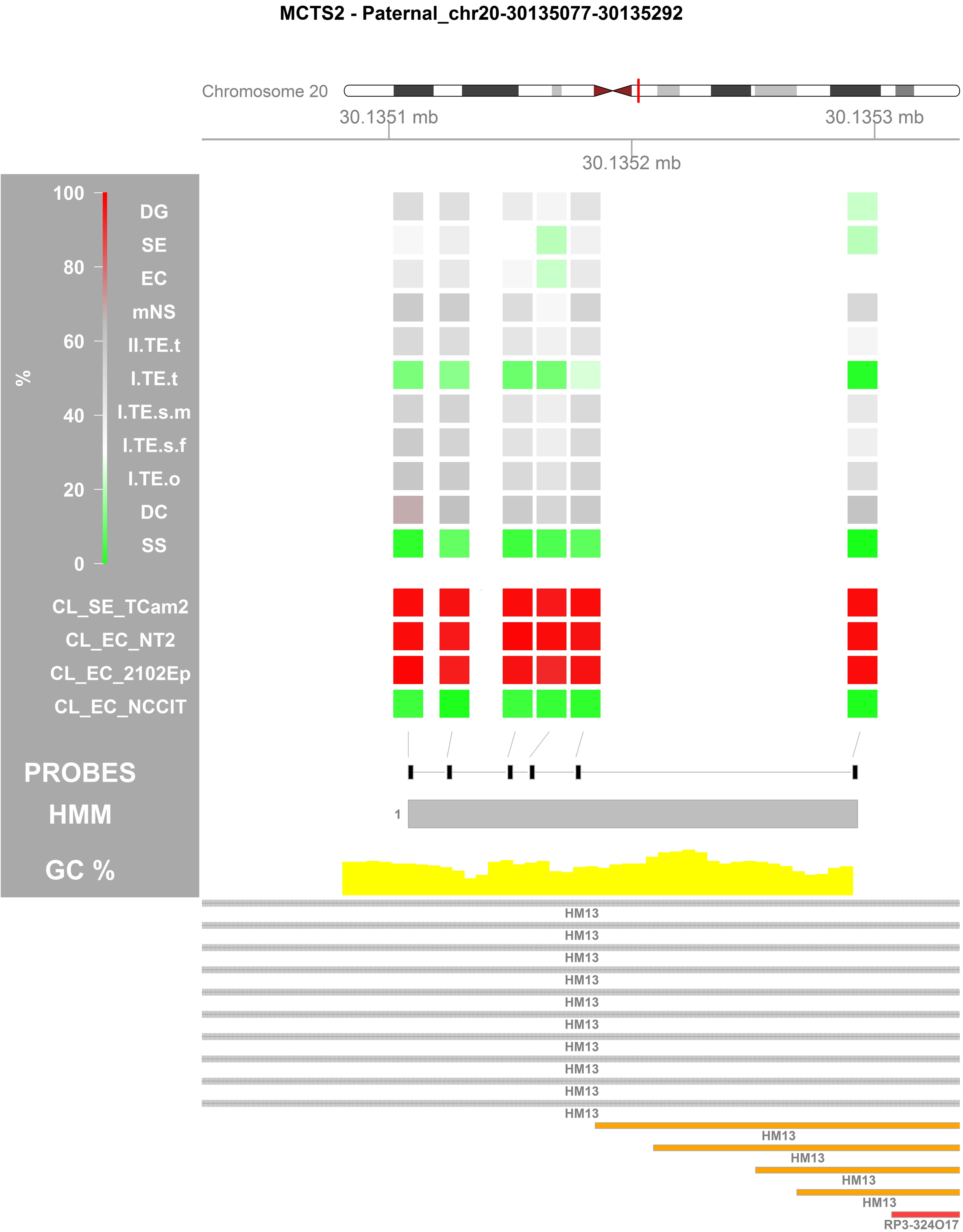

MCTS2 - Paternal_chr20-30135077-30135292
Chromosome 20
30.1351 mb
30.1352 mb
30.1353 mb
100
80
60
40
20
0
%
DG
SE
EC
mNS
II.TE.t
I.TE.t
I.TE.s.m
I.TE.s.f
I.TE.o
DC
SS
CL_SE_TCam2
CL_EC_NT2
CL_EC_2102Ep
CL_EC_NCCIT
PROBES
HMM
1
GC %
HM13
RP3-324O17

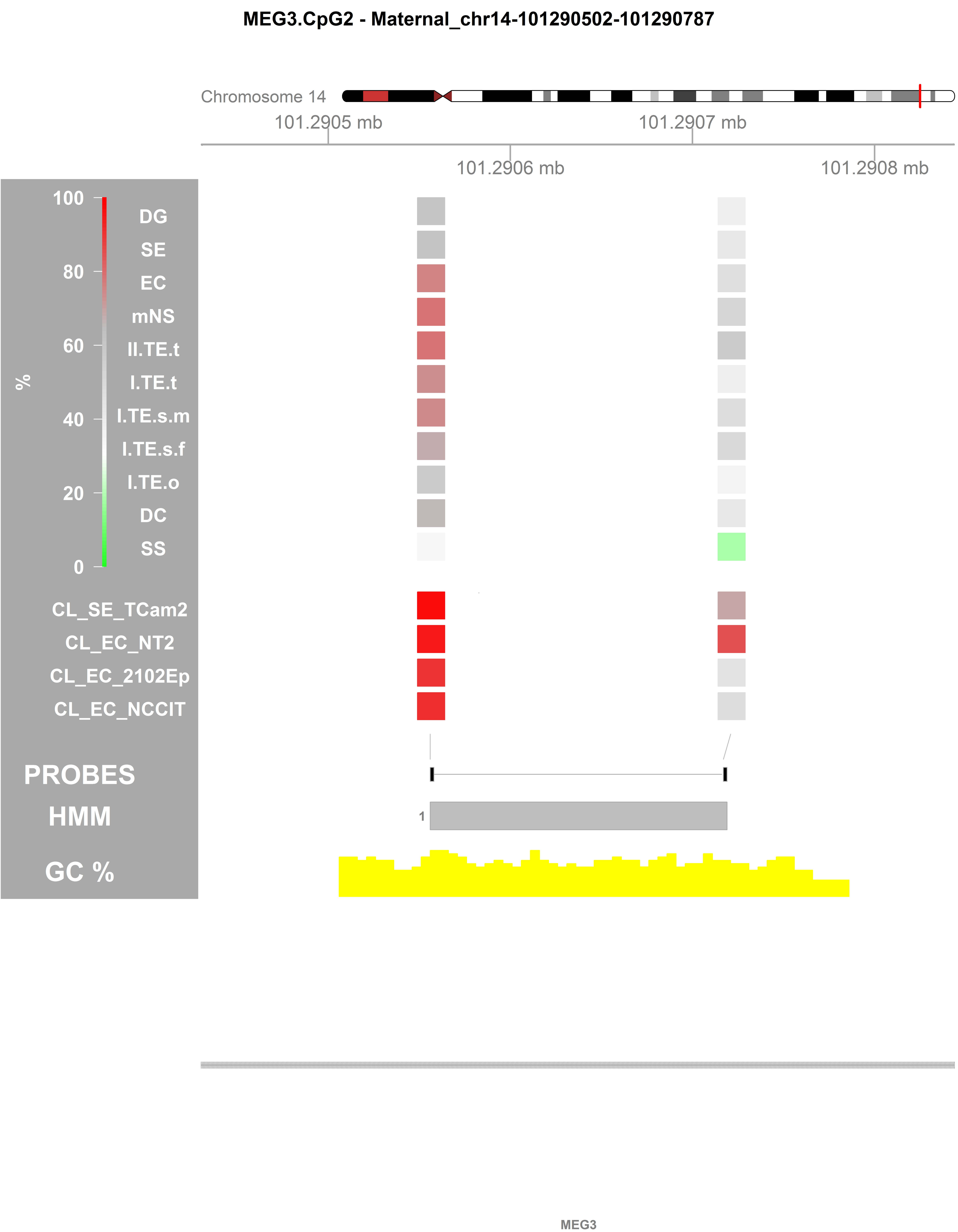
MEG3.CpG2 - Maternal_chr14-101290502-101290787
Chromosome 14
101.2905 mb
101.2906 mb
101.2907 mb
101.2908 mb
100
80
60
40
20
0
%
DG
SE
EC
mNS
II.TE.t
I.TE.t
I.TE.s.m
I.TE.s.f
I.TE.o
DC
SS
CL_SE_TCam2
CL_EC_NT2
CL_EC_2102Ep
CL_EC_NCCIT
PROBES
HMM
1
GC %
MEG3

MEG3.CTCF - Maternal_chr14-101294222-101294655

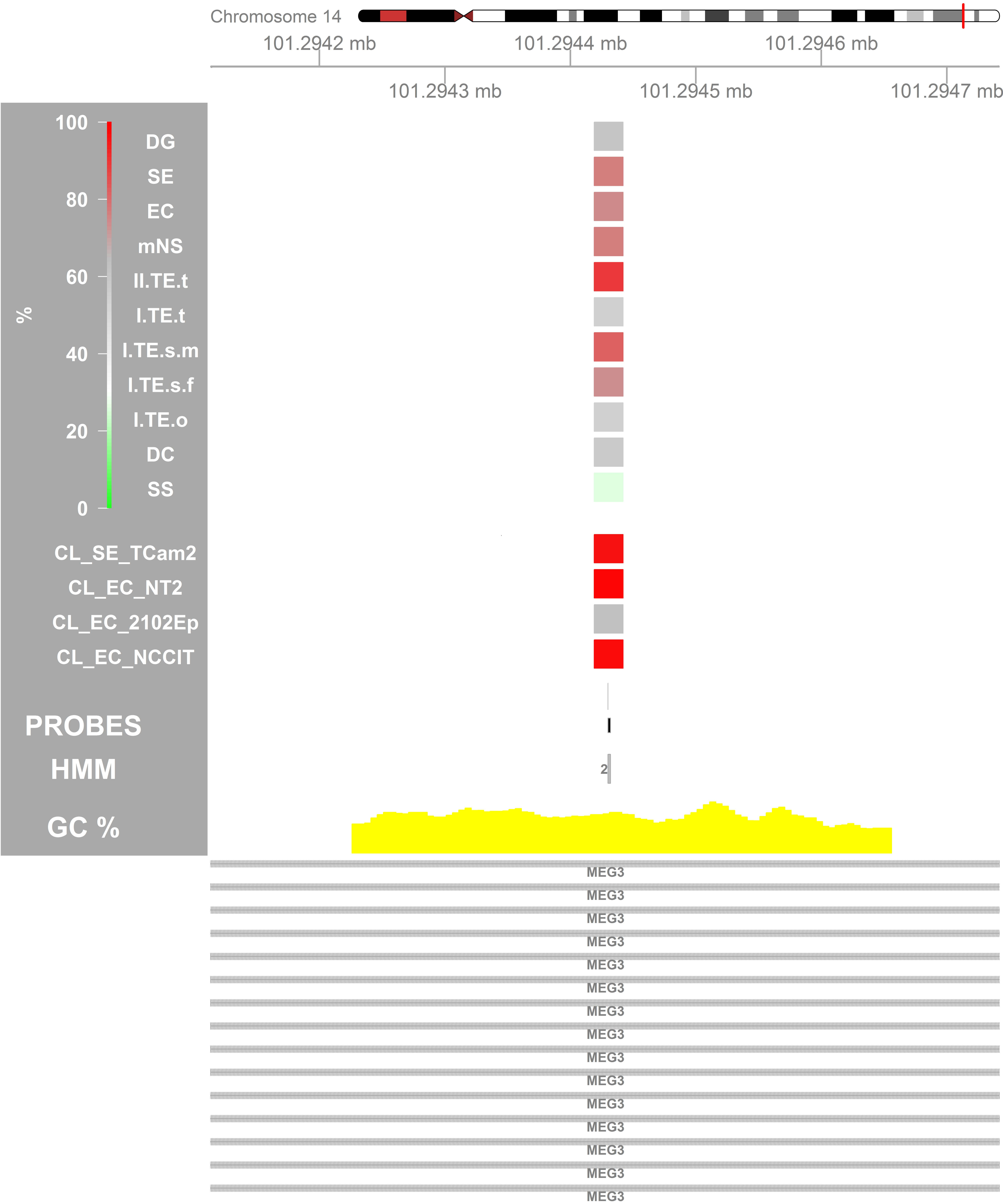

MEST - Paternal_chr7-130130740-130133111

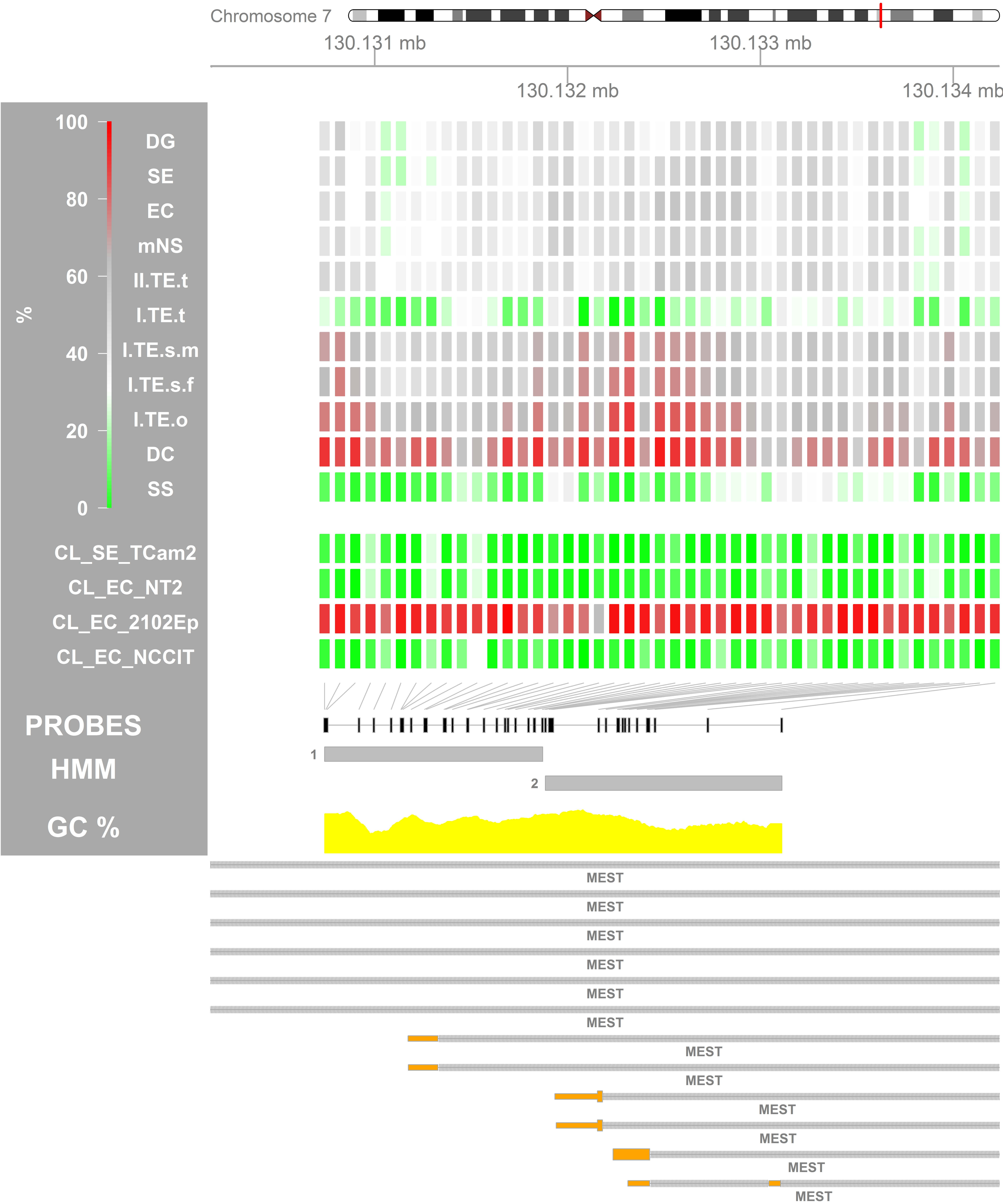

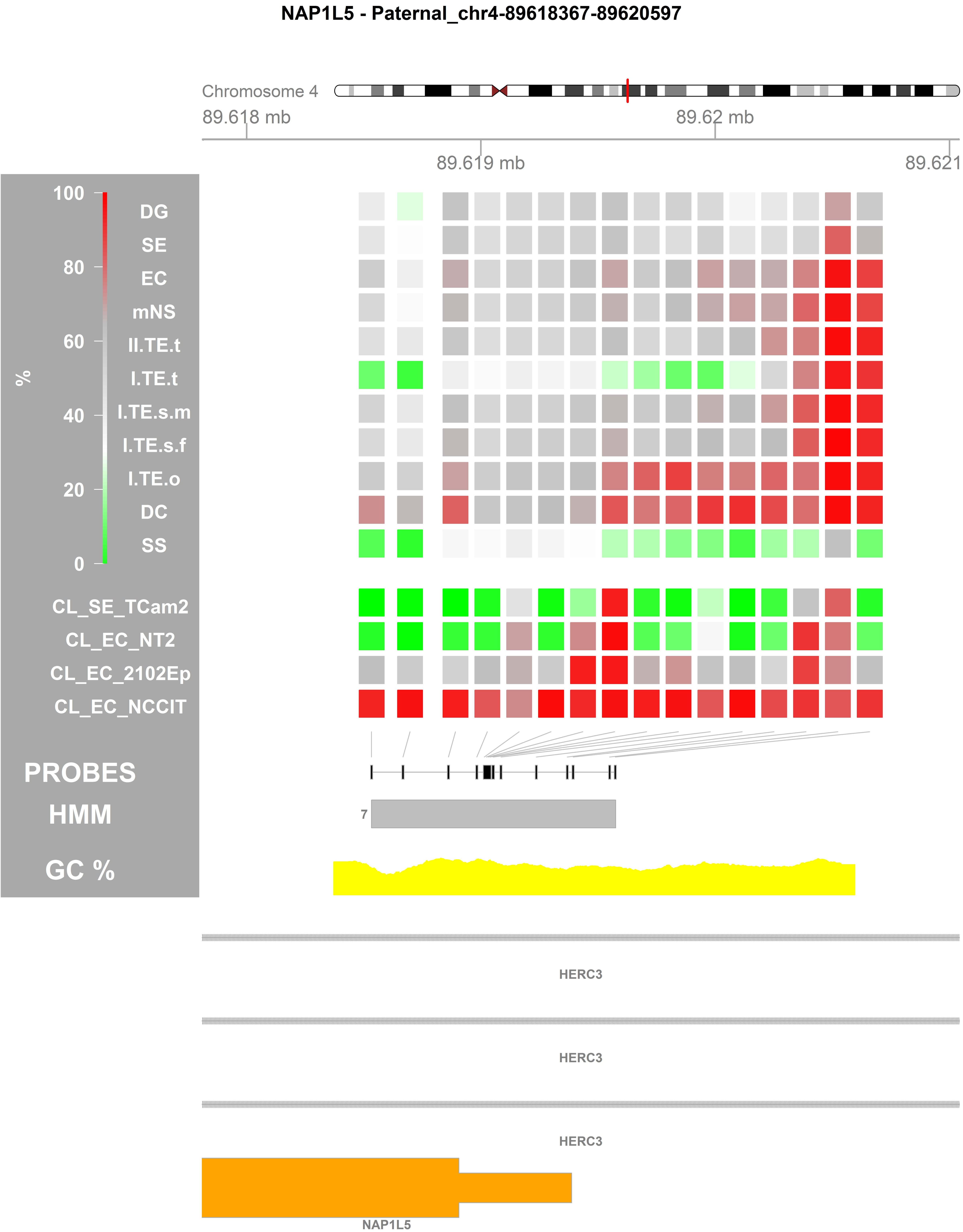
NAP1L5 - Paternal_chr4-89618367-89620597
Chromosome 4
89.618 mb
89.619 mb
89.62 mb
89.621
100
80
60
40
20
0
%
DG
SE
EC
mNS
II.TE.t
I.TE.t
I.TE.s.m
I.TE.s.f
I.TE.o
DC
SS
CL_SE_TCam2
CL_EC_NT2
CL_EC_2102Ep
CL_EC_NCCIT
PROBES
HMM
7
GC %
HERC3
HERC3
HERC3
NAP1L5

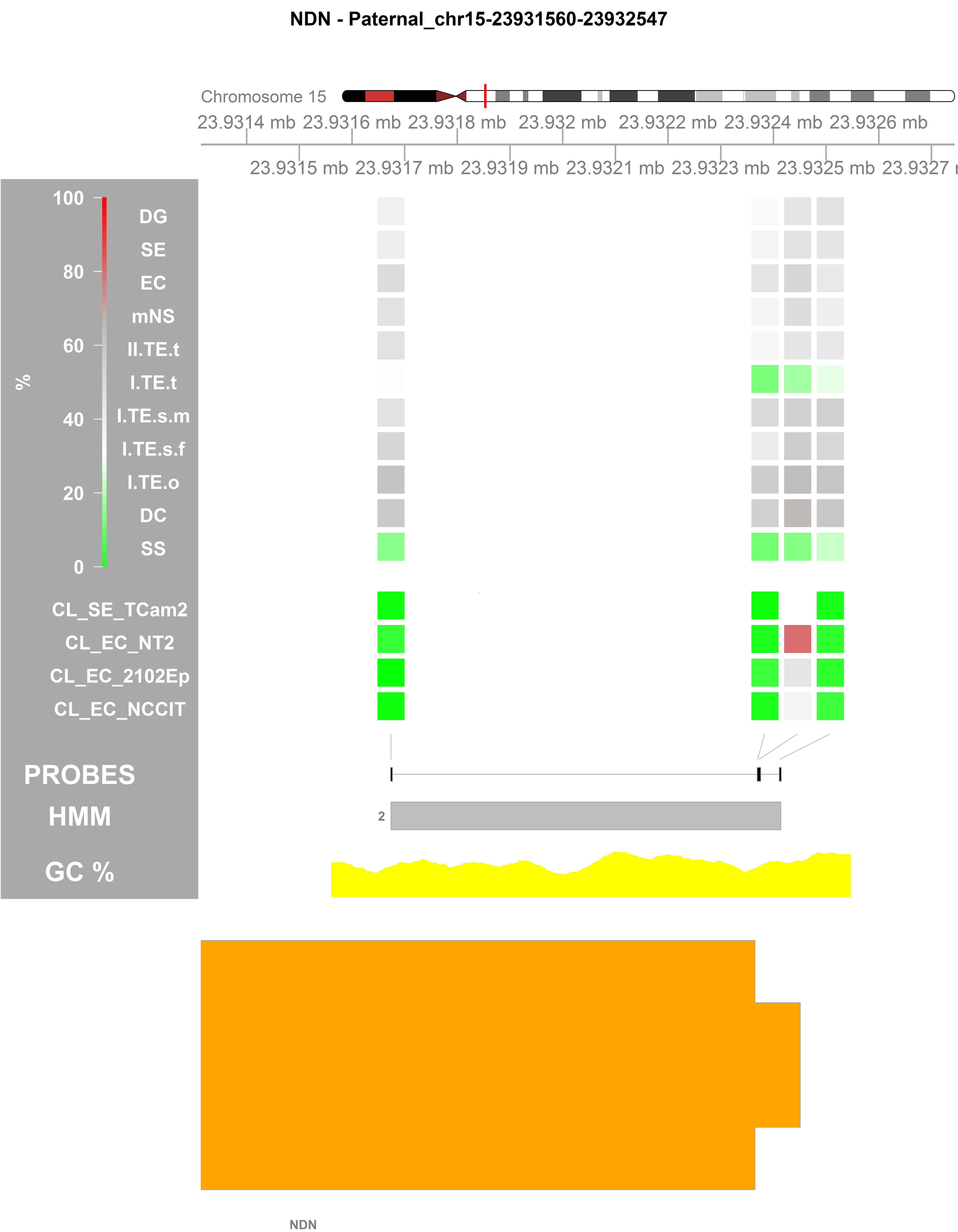
NDN - Paternal_chr15-23931560-23932547
Chromosome 15
23.9314 mb 23.9316 mb 23.9318 mb 23.932 mb 23.9322 mb 23.9324 mb 23.9326 mb
23.9315 mb 23.9317 mb 23.9319 mb 23.9321 mb 23.9323 mb 23.9325 mb 23.9327 mb
%
100
80
60
40
20
0
DG
SE
EC
mNS
II.TE.t
I.TE.t
I.TE.s.m
I.TE.s.f
I.TE.o
DC
SS
CL_SE_TCam2
CL_EC_NT2
CL_EC_2102Ep
CL_EC_NCCIT
PROBES
HMM
2
GC %
NDN

NNAT - Paternal_chr20-36147118-36151058

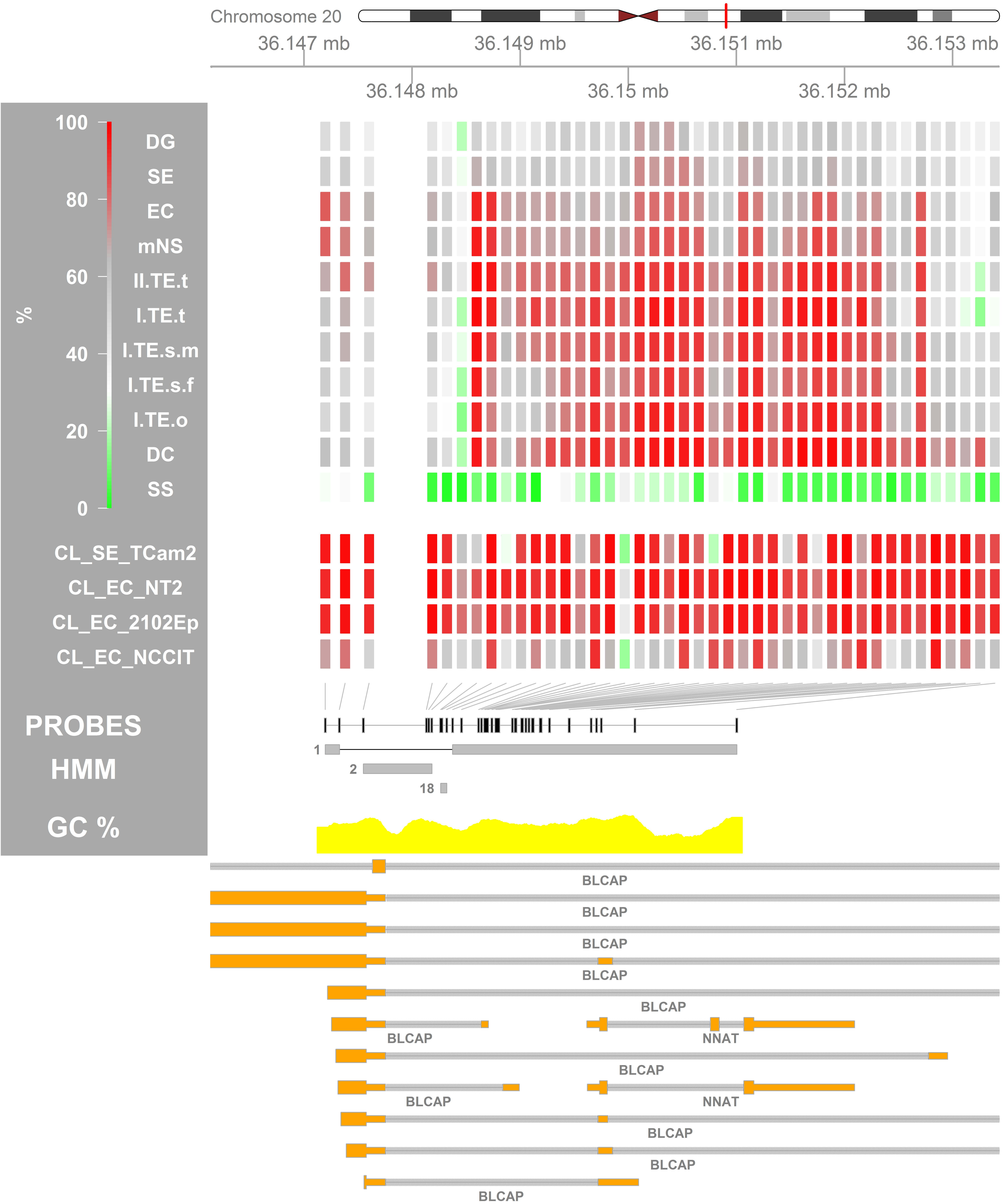

PEG13 - Paternal_chr8-141107838-141110984

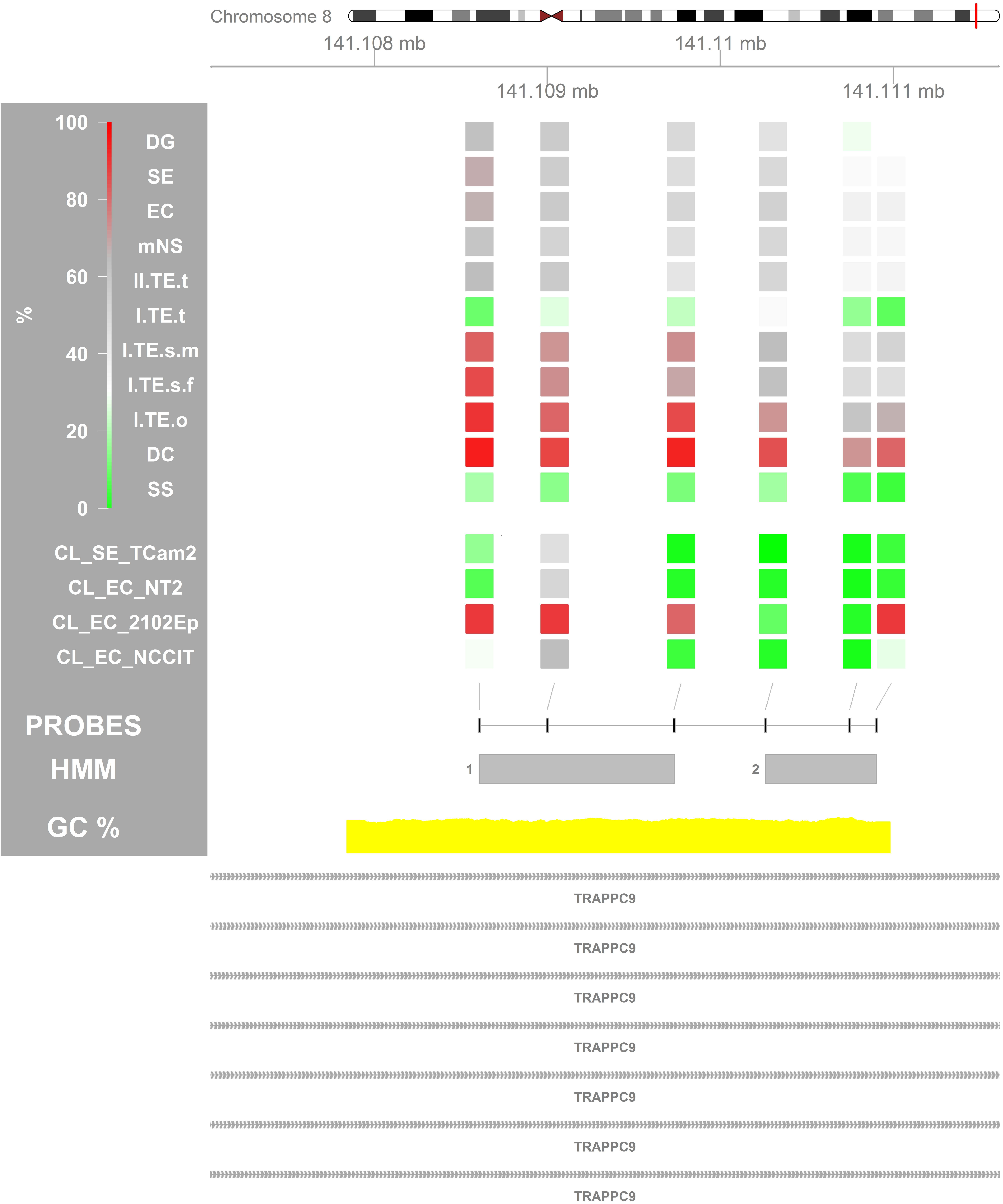

SGCE, PEG10 - Paternal_chr7-94284859-94286557

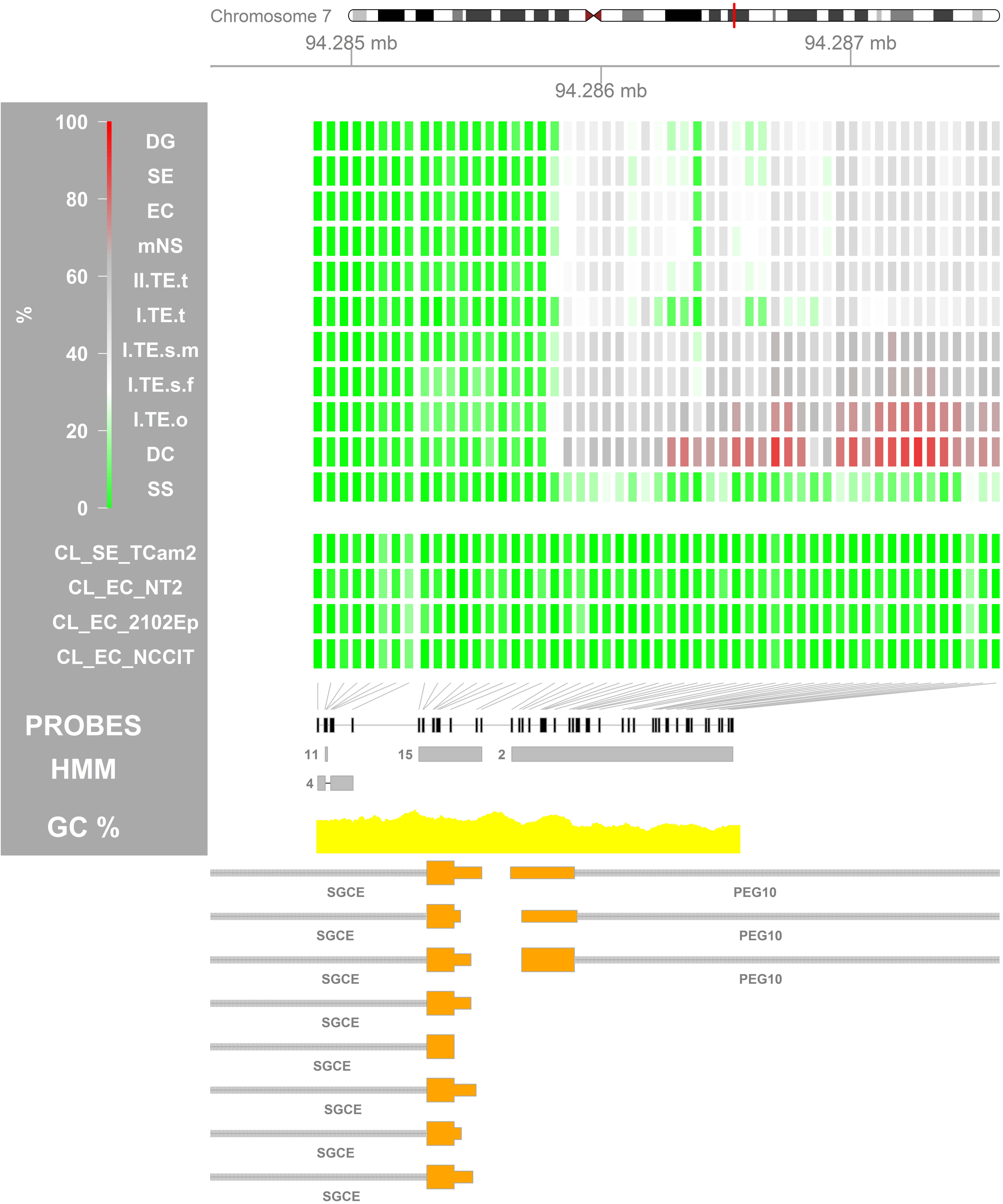

SNURF_SNRPN, SNURF-SNRPN - Paternal_chr15-25199934-25200343

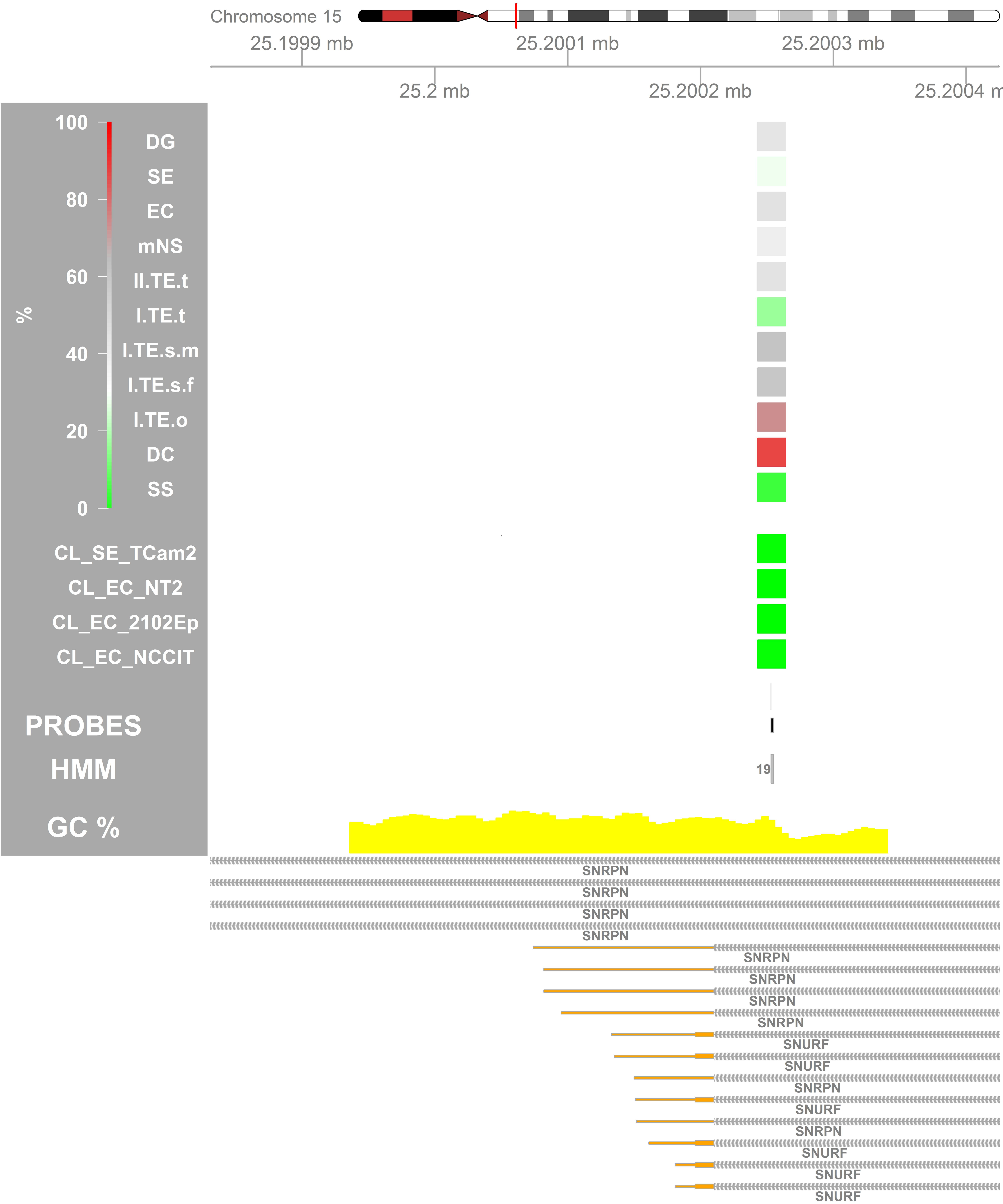

TCEB3C - Unknown_chr18-44554880-44556671

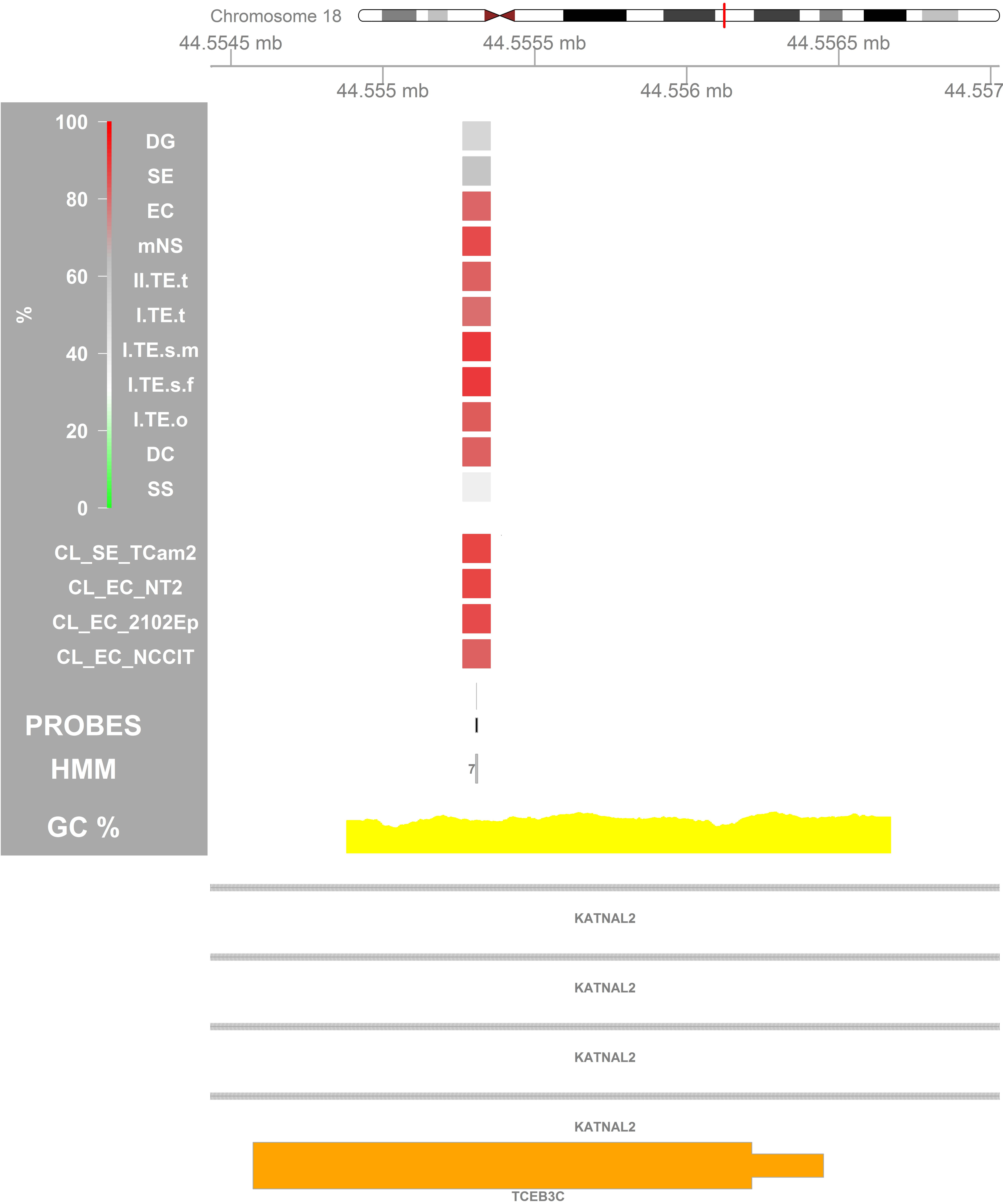

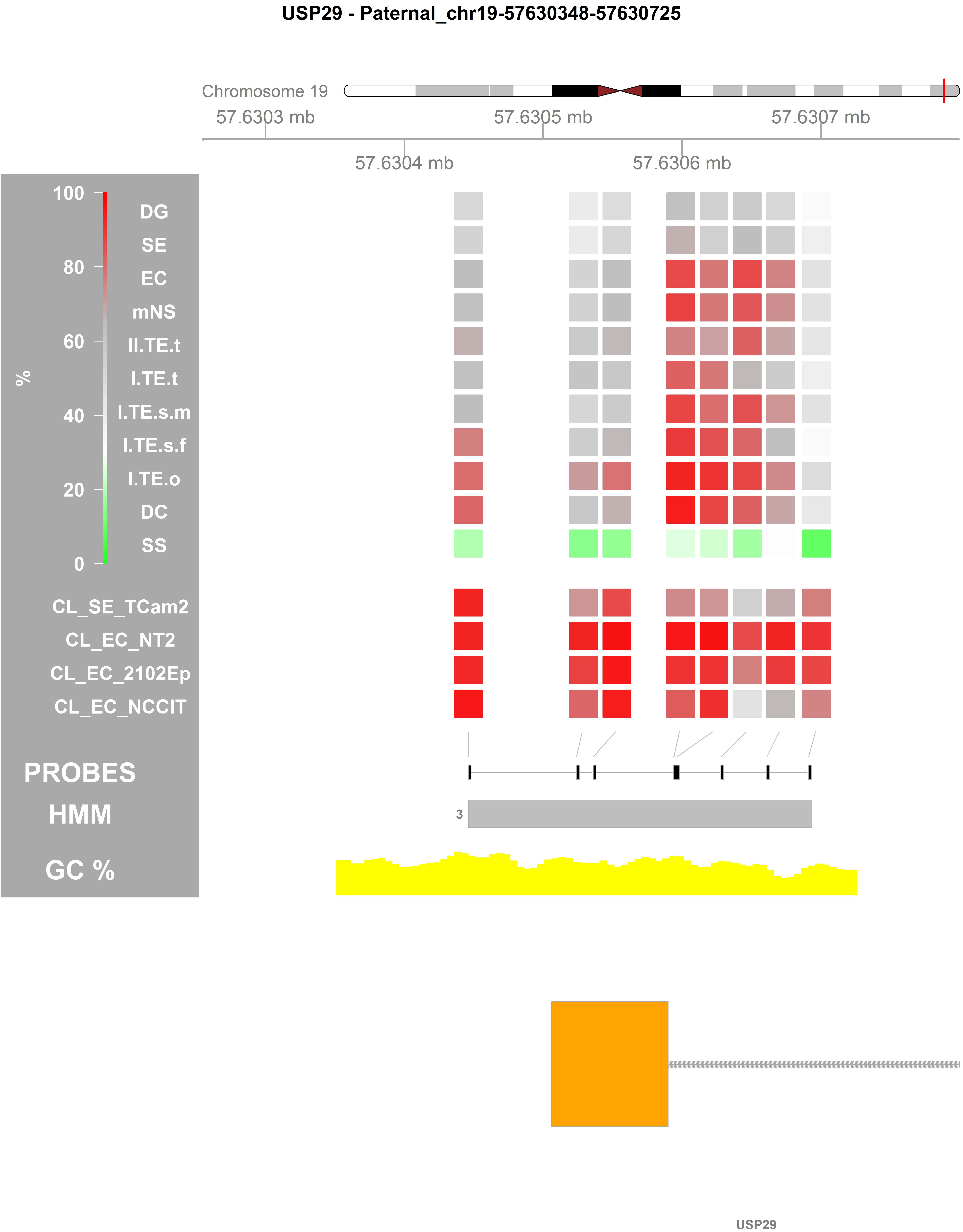
USP29 - Paternal_chr19-57630348-57630725
Chromosome 19
57.6303 mb
57.6304 mb
57.6305 mb
57.6306 mb
57.6307 mb
100
80
60
40
20
0
%
DG
SE
EC
mNS
II.TE.t
I.TE.t
I.TE.s.m
I.TE.s.f
I.TE.o
DC
SS
CL_SE_TCam2
CL_EC_NT2
CL_EC_2102Ep
CL_EC_NCCIT
PROBES
HMM
3
GC %
USP29

WT1.ARR, WT1-AS - Paternal, Unknown_chr11-32453776-32455394

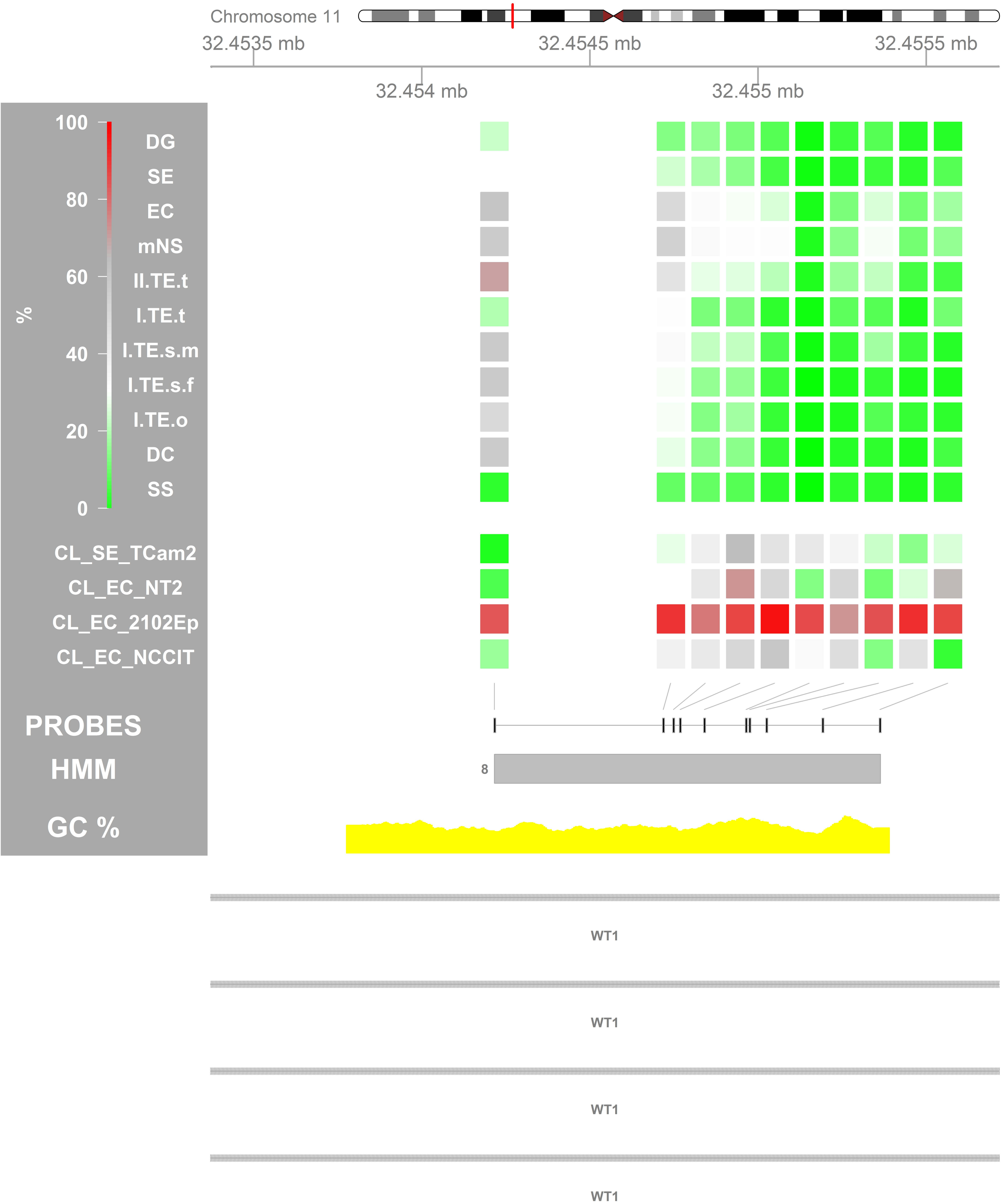

ZAC_HYMAI, ZAC1 - Paternal_chr6-144328917-144329947

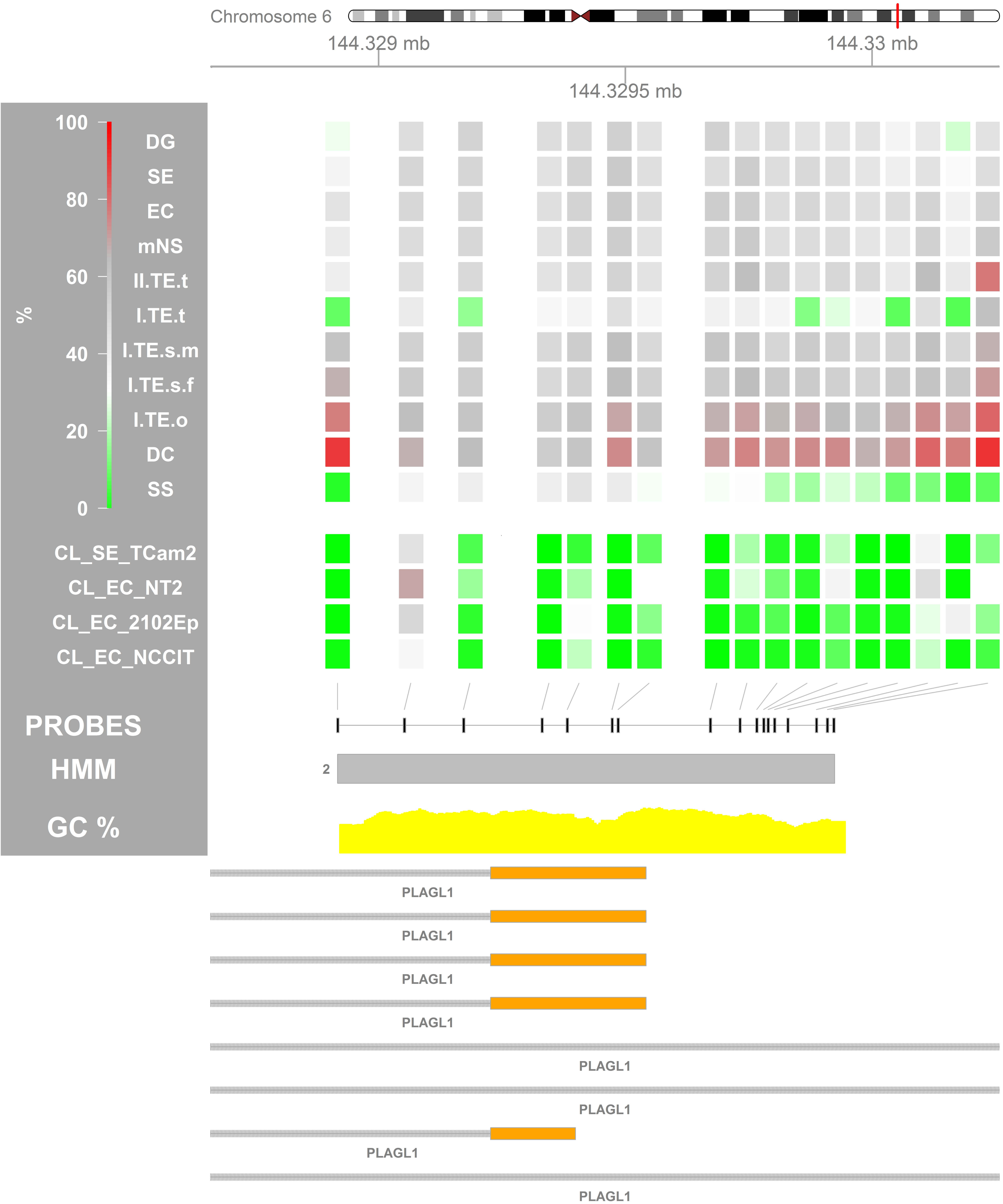

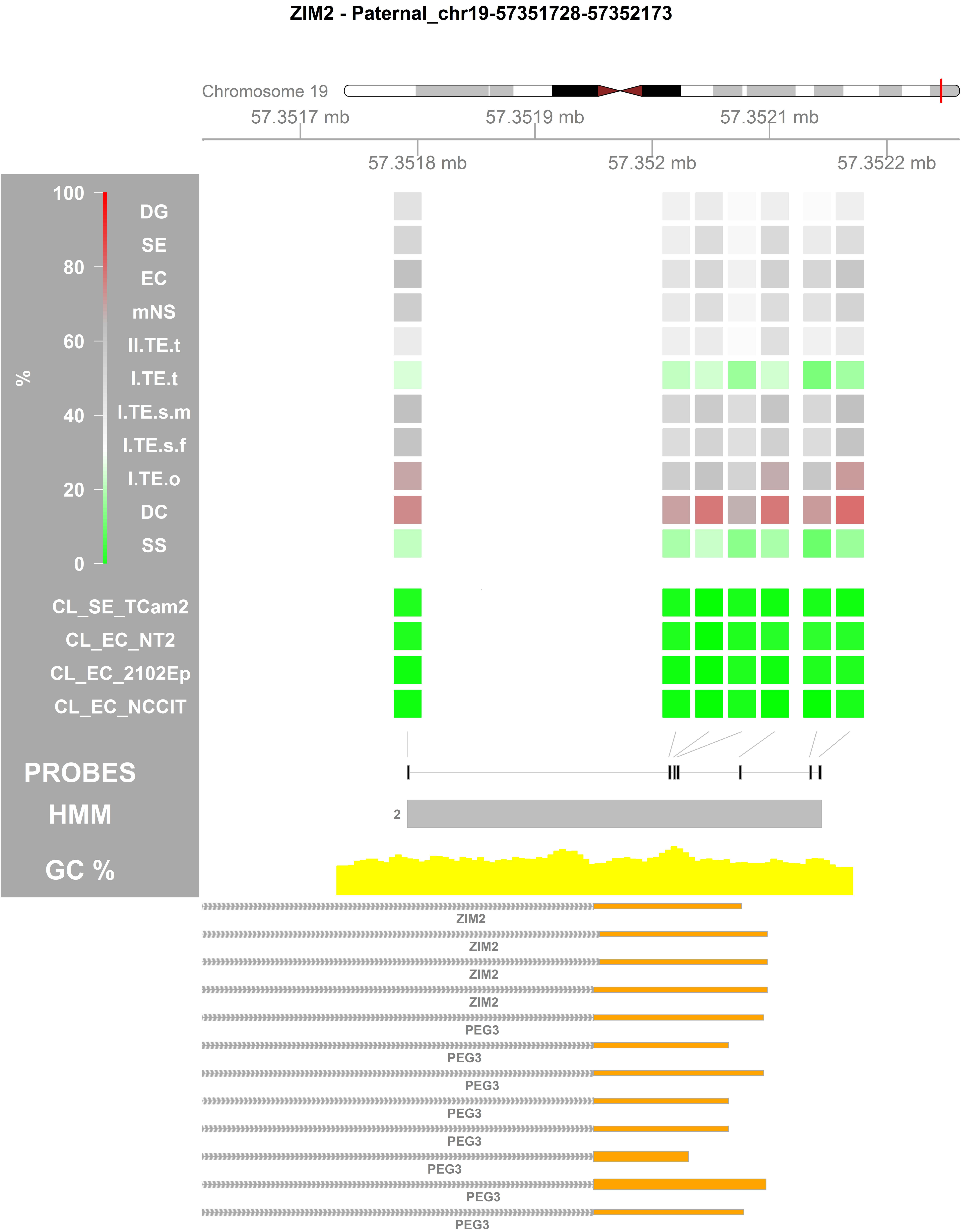

ZIM2 - Paternal_chr19-57351728-57352173
Chromosome 19
57.3517 mb
57.3519 mb
57.3521 mb
57.3518 mb
57.352 mb
57.3522 mb
100
80
60
40
20
0
%
DG
SE
EC
mNS
II.TE.t
I.TE.t
I.TE.s.m
I.TE.s.f
I.TE.o
DC
SS
CL_SE_TCam2
CL_EC_NT2
CL_EC_2102Ep
CL_EC_NCCIT
PROBES
HMM
2
GC %
ZIM2
ZIM2
ZIM2
ZIM2
PEG3
PEG3
PEG3
PEG3
PEG3
PEG3
PEG3
PEG3
