# Supplementary material for: Correction: Genome Wide DNA Methylation Profiles Provide Clues to the Origin and Pathogenesis of Germ Cell Tumors
Source: PLoS One. 2015 Jul 14;10(7):e0132981. doi: 10.1371/journal.pone.0132981 (PMC4501728; doi:10.1371/journal.pone.0132981)
Supplement: S2 File — (PDF) [file pone.0132981.s002.pdf]

PLOS | ONE

RESEARCH ARTICLE

Genome Wide DNA Methylation Profiles Provide Clues to the Origin and Pathogenesis of Germ Cell Tumors

Martin A. Rijlaarsdam[1], David M. J. Tax[2], Ad J. M. Gillis[1], Lambert C. J. Dorssers[1], Devin C. Koestler[3], Jeroen de Ridder[2], Leendert H. J. Looijenga[1]*

1 Department of Pathology, Erasmus MC Cancer Institute—University Medical Center Rotterdam, Rotterdam, The Netherlands, **2** Faculty of Electrical Engineering, Mathematics and Computer Science Intelligent Systems—Delft Bioinformatics Lab, Technical University of Delft, Delft, The Netherlands, **3** Department of Biostatistics, University of Kansas Medical Center, Kansas City, Kansas, United States of America

* l.looijenga@erasmusmc.nl

CrossMark
click for updates

OPEN ACCESS

Citation: Rijlaarsdam MA, Tax DMJ, Gillis AJM, Dorssers LCJ, Koestler DC, de Ridder J, et al. (2015) Genome Wide DNA Methylation Profiles Provide Clues to the Origin and Pathogenesis of Germ Cell Tumors. PLoS ONE 10(4): e0122146. doi:10.1371/journal.pone.0122146

Academic Editor: Osman El-Maarri, University of Bonn, Institut of experimental hematology and transfusion medicine, GERMANY

Received: September 17, 2014

Accepted: February 7, 2015

Published: April 10, 2015

Copyright: © 2015 Rijlaarsdam et al. This is an open access article distributed under the terms of the Creative Commons Attribution License, which permits unrestricted use, distribution, and reproduction in any medium, provided the original author and source are credited.

Data Availability Statement: All data is available via GEO (GSE58538).The extended annotation for the Illumina 450K platform including its documentation is available at GEO (GPL18809).

Funding: MR is supported by a Translational Grant, Erasmus MC. The funder had no role in study design, data collection and analysis, decision to publish, or preparation of the manuscript.

Competing Interests: The authors have declared that no competing interests exist.

Abstract

The cell of origin of the five subtypes (I-V) of germ cell tumors (GCTs) are assumed to be germ cells from different maturation stages. This is (potentially) reflected in their methylation status as fetal maturing primordial germ cells are globally demethylated during migration from the yolk sac to the gonad. Imprinted regions are erased in the gonad and later become uniparentally imprinted according to fetal sex. Here, 91 GCTs (type I-IV) and four cell lines were profiled (Illumina's HumanMethylation450BeadChip). Data was pre-processed controlling for cross hybridization, SNPs, detection rate, probe-type bias and batch effects. The annotation was extended, covering snRNAs/microRNAs, repeat elements and imprinted regions. A Hidden Markov Model-based genome segmentation was devised to identify differentially methylated genomic regions. Methylation profiles allowed for separation of clusters of non-seminomas (type II), seminomas/dysgerminomas (type II), spermatocytic seminomas (type III) and teratomas/dermoid cysts (type I/IV). The seminomas, dysgerminomas and spermatocytic seminomas were globally hypomethylated, in line with previous reports and their demethylated precursor. Differential methylation and imprinting status between subtypes reflected their presumed cell of origin. Ovarian type I teratomas and dermoid cysts showed (partial) sex specific uniparental maternal imprinting. The spermatocytic seminomas showed uniparental paternal imprinting while testicular teratomas exhibited partial imprinting erasure. Somatic imprinting in type II GCTs might indicate a cell of origin after global demethylation but before imprinting erasure. This is earlier than previously described, but agrees with the totipotent/embryonic stem cell like potential of type II GCTs and their rare extra-gonadal localization. The results support the common origin of the type I teratomas and show strong similarity between ovarian type I teratomas and dermoid cysts. In conclusion, we identified specific and global methylation differences between GCT subtypes, providing insight into their developmental timing and underlying developmental biology. Data and extended annotation are deposited at GEO (GSE58538 and GPL18809).

Introduction

During fetal development primordial germ cells (PGC) migrate from the yolk sac, via the hindgut to the genital ridge and enter the gonad where they undergo further maturation into the sex specific lineage, i.e. oogonia for females and spermatogonia for males. During migration and maturation an epigenetic "reset" takes place. This includes global DNA CpG demethylation during the early phases of migration. Specific areas like imprinted regions remain methylated until the PGCs arrive in the developing gonads where imprinting is subsequently gradually erased. After these maturing gonadal germ cells reach mitotic (male) or meiotic (female) arrest, *de novo* methylation is initiated and uniparental sex specific imprinting is acquired [1–8]. Another informative marker of developmental stage is X chromosome reactivation which occurs in female germ cells before the initiation of oogenesis. Studies report varying results regarding the exact timing of the various steps of the epigenetic reset, i.e. during migration or after arrival in the gonads. However, PGCs with an XX chromosomal constitution have been shown to lack X chromosome reactivation if they never reach the gonad [9–12]. For ethical reasons, most of these data have been experimentally investigated and validated in mice. Even though germ cell development differs between mice and men [13], methylation patterns during germ cell development are reported to be highly similar [14,15].

Germ cell tumors (GCT) originate from germ cells at different developmental stages and are thought to inherit their methylation profile from their ancestors. The WHO classification supports five GCT subtypes. Each subtype has specific molecular, clinical and histopathological properties [16–19]. GCT subtypes have been put in context of normal germ cell development (Fig 1A) based on gene/microRNA expression, (targeted) epigenetic analysis and genomic constitution as described below and reviewed extensively elsewhere [13,16,17,20–22]. Most of these studies were targeted at specific genes/genomic regions or concerned a subset of the GCT subtypes only, most prominently type I or II.

Type I ("infantile") GCTs manifest clinically as teratoma (TE) and/or yolk sac tumor (YS) along the migration route of developing PGCs, i.e. the midline of the body. Extra-gonadal, sacral TEs occur most frequently and are mostly benign. Typically these rare tumors (incidence 0.12/100 000) arise before the age of 6 and no *Carcinoma In Situ* (CIS, see below) is found. They show global methylation patterns that are reminiscent of their embryonic stem cell progenitor (i.e. bimodal with modes at ≈0 and ≈100% methylation). These tumors showed somatic/biparental (≈50%) imprinting status in earlier studies. Therefore, type I GCTs have been suggested to originate from PGCs at an early stage, prior to global demethylation and imprinting erasure [16–18,23–25].

Type II GCTs present most frequently in the gonads and are also called germ cell cancer (GCC). The incidence of these tumors peaks between 25–35 years of age depending on the subtype [16,17,19]}. They comprise ≈1% of all solid cancers in Caucasian males and are responsible for 60% of all malignancies diagnosed in men between 20 and 40 years with increasing incidence in the last decades [26] (8.38/100,000 Dutch population. Dutch Caner Registration (IKNL), www.cijfersoverkanker.nl). Risk factors have been thoroughly investigated and are integrated in a genvironmental risk model, in which risk is determined by a combination of micro/macro-environmental and (epi)genetic factors [19,26–32]. A common precursor lesion called CIS or intratubular germ cell neoplasia unclassified (IGCNU, WHO definition [18]) is identified for type II GCT [16,17,33,34]. Because of the non-epithelial origin these tumors, CIS is technically not a proper term but will be used throughout this article in the interest of consistency with existing literature. Type II GCT consist of non-seminomatous (NS) and seminomatous (SE) tumors (Fig 1A), which differ in clinical behavior and molecular profile. SE and embryonal carcinoma (EC) are the stem cell components of type II GCT and EC can further

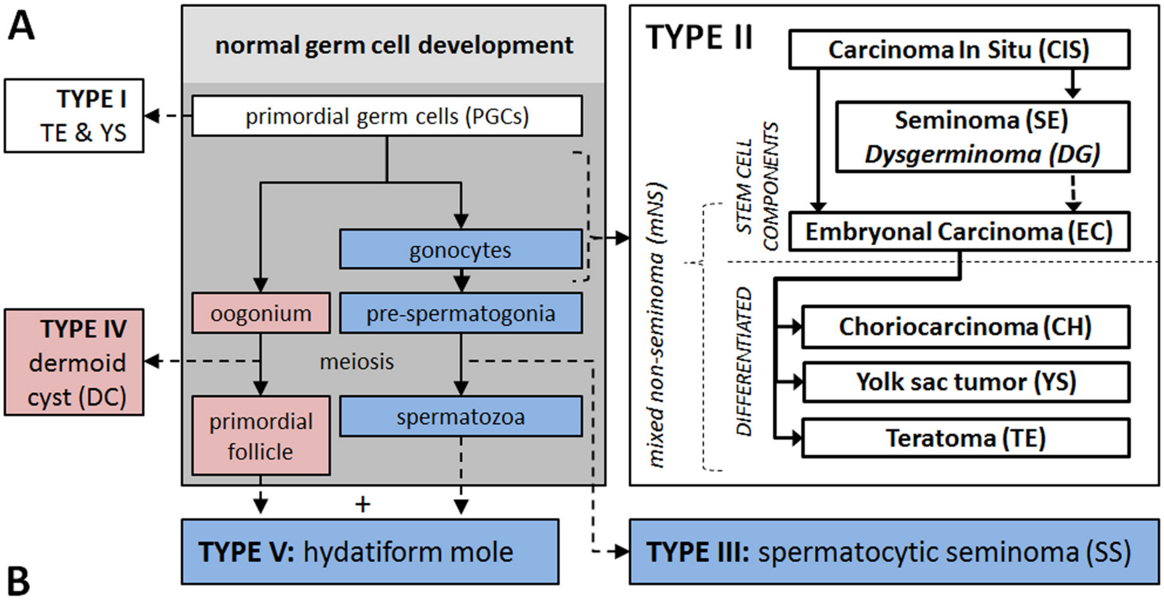

B

GCT subtype	n	
I: TE	14	6 sacrum (male:3 [I.TE.s.m], female: 3 [I.TE.s.f]), 4 testis [I.TE.t], 4 ovary [I.TE.o]
II: EC	30	
II: mNS	24 (3 pure type II TE)	
II: SE	12	
II: DG	4	
III: SS	4	
IV: DC	3	
Cell lines (type)	TCam-2 (SE), NT2 (EC), 2102EP (EC), NCCIT (EC)	

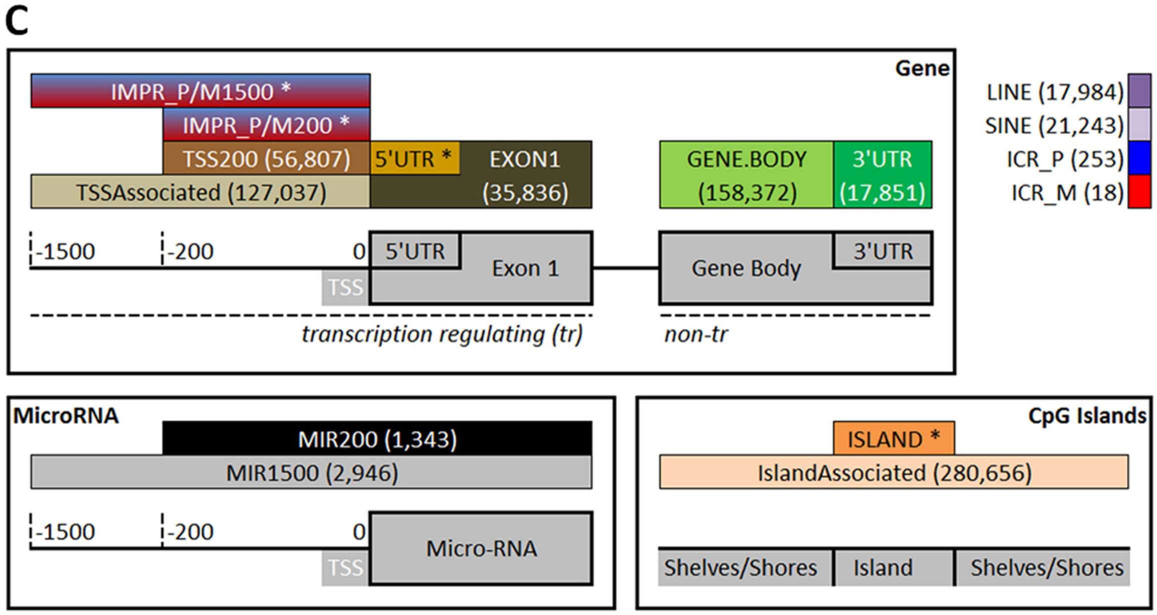

Fig 1. Tumor types/samples and cell lines analyzed and schematic visualization of genomic functional categories of interest. (A) GCT subtypes in the hypothesized context of normal germ cell development as proposed in earlier studies (grey box). Developmental schemes are indicated in blue (male), red (female) or when possible in both sexes (white). DG does not originate from CIS but is indicated together with SE for reasons of consistency. **(B)** Samples included in this study. Abbreviations match Fig 1A and roman numbers indicate the GCT type to which the histological subtypes belongs. n indicates the number of tumor samples per group. All samples are from male patients except the DGs, DCs and a subset of the type I TEs. Please note that when only TE

is denoted, this indicates the group of all type I TEs together. Otherwise II.TE (type II pure TE) or the abbreviations for specific localizations are used as indicated in this figure. Four GCT cell lines were included; tumor of origin between brackets. **(C)** Reference to (abbreviations of) the functional genomic regions as mentioned in the rest of the manuscript. Probes were classified according to their relation to gene coding regions, micro-RNA (MIR) coding regions, CpG islands and/or transposon elements (LINE/SINE). The distance to the transcription start site (TSS) was used in accordance with the Illumina manifest: 200 or 1500 bp. Of note, the TSSAssociated category contains all probes with a distance < 1500 bp to the TSS in contrast to the TSS1500 category from Illumina which is only contains probes 200-1500bp from the TSS. Probes within imprinting associated regions were classified as (1) mapped inside a known imprinting control region (ICR) or (2) either mapped inside an ICR or mapped close to the TSS of a transcript of an imprinted gene (200/1500bp upstream, not mutually exclusive). P/M indicates the expressed allele, i.e. paternal/maternal respectively. Numbers between brackets indicate the number of valid probes within each specific category (total number of valid probes: 437,881). *The visualization did not permit including the probe count for all categories. The counts for the empty categories are: 5'UTR = 59,338; ISLAND = 136,339; IMPR_P200 = 638; IMPR_P1500 = 1,659; IMPR_M200 = 610; IMPR_M1500 = 2,265.

doi:10.1371/journal.pone.0122146.g001

differentiate in the other NS subtypes: TE, YS and choriocarcinoma (CH) [16,17]. Type II GCT originate from maturation arrested, germ line committed PGCs or gonocytes and historically have been suggested to exhibit erasure of genomic imprinting [13,16–19,22,35]}

Type III, IV and V GCTs originate from more differentiated germ cell progenitor cells. Type III GCTs are also known as spermatocytic seminoma (SS) and occur solely in the testis. They arise after the age of 50 and are generally benign and rare (incidence: 0.2/100000). Their presentation in elderly males, morphology and immunohistochemical profile separates SS from SE. They originate from germ cells around the spermatogonium stage and are paternally imprinted [16,36–40]. Type IV tumors are historically hypothesized to originate from a maternally imprinted, committed female germ cell. Type V GCT were excluded from this study because they show an independent pathogenesis. They originate from the fertilization of an empty ovum by two sperm cells, resulting in a completely paternally imprinted genomic constitution. This explains their mono-directional lineage of differentiation, unrelated to the germ cell origin [16–18].

This study aims to identify specific and global differences between the genome-wide methylation profiles of GCT subtypes. Type I, II, III and IV GCTs and four cell lines representative of type II GCTs are investigated (Fig 1A and 1B). Differences in methylation profile provides insight into the developmental timing and underlying biology of GCTs. The findings ultimately relate GCT subtypes to specific stages of (early) developing (embryonic) germ cells. Emphasis was placed on combining the results with the available literature and on providing extensive accompanying data to supply an integrated, hypothesis generating data source for future research.

Results

Methylation differences were investigated, starting from global methylation profiles, followed by functional enrichment analysis. Probes were functionally classified according to their relation to genes: transcription regulating (200 or 1500bp upstream of the TSS & 5'UTR) or gene coding (exon 1, gene body and 3'UTR). Probes covering micro-RNA (MIR) coding regions, CpG islands and/or transposon elements (LINE/SINE) were classified separately as were imprinting associated genes. For a detailed explanation, please see Fig 1C and the Materials and Methods section (section: (Additional) annotation 450K array). After functional enrichment analysis, specific differentially methylated probes were identified (DMPs). Probes represent individual CpG sites. Also, differentially methylated regions (DMRs) containing multiple adjacent probes were identified. Finally, imprinting status was evaluated. Please note that differential methylation indicates a statistically significant difference after correction for multiple testing, unless specifically stated otherwise. Differential methylation of $\Delta M > |0.9|$ was considered relevant, in line with the recommendations of Du et al [41]. For details about the statistical procedures, please see the materials and methods section (analysis protocol). Abbreviations are explained in (the legend of) Fig 1.

SS and SE/DG show global hypomethylation when compared to EC/mNS and TE

Fig 2A shows the methylation distributions for all probes, probes associated with the TSS, 3' UTR, LINES, microRNAs and CpG Islands, respectively. The distributions of the remaining functional categories are presented in S2A Fig. SS showed global hypomethylation (Fig 2A), i.e. a large concentration of probes showing a low percentage of methylation and few probes showing a high methylation percentage. Hypermethylated configurations contain a large concentration of probes showing a high percentage of methylation and few probes showing a low methylation percentage. Hypomethylation was also shown in DG and SE samples albeit to a lesser extent, as can be observed from the mode at 50–60% methylation (Fig 2A). The SE group showed consistent hypomethylation (S2B Fig, page 2), in contrast to study of Nettersheim et al who showed separate groups of hypo- and hypermethylated SE in a larger sample series [42]. In contrast to the SE and DG samples, the EC and partly differentiated mNS, type I TE and DC samples consistently showed a bimodal pattern with one mode around 10% and one around 90% (Fig 2A and Fig 1: relation between subtypes). This bimodal pattern was also observed in three EC cell lines and a single SE cell line (Fig 2A, CL_SE & CL_EC). In line with previous reports [14,43], the EC cell lines were more methylated than the SE cell TCam-2 (Fig 2A). The transcription regulatory region upstream of the TSS (TSSAssociated, TSS200) was generally hypomethylated in all tumor types as were regions annotated as first exon, 5'UTR and CpG islands. The gene body, 3'-UTR, micro-RNAs and LINE/SINE elements were generally hypermethylated except in SS, which show a bimodal pattern (Fig 2A and S2A Fig). At these sites, SE/DG showed a median methylation level of 50% in line with the maximal methylation of their global profile and previous reports [20,44]. Hypermethylation of LINE/SINE elements NS and hypomethylation (Fig 2A) in SE was in line with a recent genome wide study [20] but contrasted with a targeted study that showed hypomethylation of 3 specific repetitive elements in both SE and NS [45].

GCT subtypes can be distinguished based on their methylation profile

Principal component analysis (PCA) showed robust separation of homogeneous clusters of EC/mNS, SE/DG, TE/DC and SS samples when all probes were considered (Fig 2B and S2A Fig). In line with the larger inter-sample variation (S2B Fig), SE/DG and SS were more scattered in the PCA plot. Some mNS, which consist partly of differentiated tissue, showed a tendency towards the differentiated TE/DC group. The type I TE and DC showed an indistinguishable global methylation profile. Similar observations were made when subsets of probes were considered that were annotated to specific functional genomic regions (Figs 2B and S2A).

Zooming in: GCT subtype specific methylation patterns

To further pinpoint differences between pairs of GCT subtypes, DMPs were identified (Table 1, S1 Table), tested for functional and chromosomal enrichment (Fig 3, S3 Fig, Table 1 and S2 Table) and grouped into DMRs (Fig 4, S4 Fig, Table 1, S3 Table, GSE58538: File S1). SE + DG and EC + mNS (including type II pure TE) subtypes were merged because of high similarity of the observed methylation profile (Figs 2A and 2B, S3A Fig), in line with literature regarding their similar origin [46] and their close relation in the current WHO classification [16,18]. Recurrent DMRs were identified as genes occurring more than once within or between comparisons, which may indicate regions of importance (S3 Table, n = 149).

(Differential) methylation of GCT cell lines (4136 DMRs between the cell lines: GSE58538: File S2) showed little similarity to their in vivo counterparts (Figs 2 and 6, S2 Fig). The cell line analysis did however identify a biologically relevant DMR previously validated in these cell lines

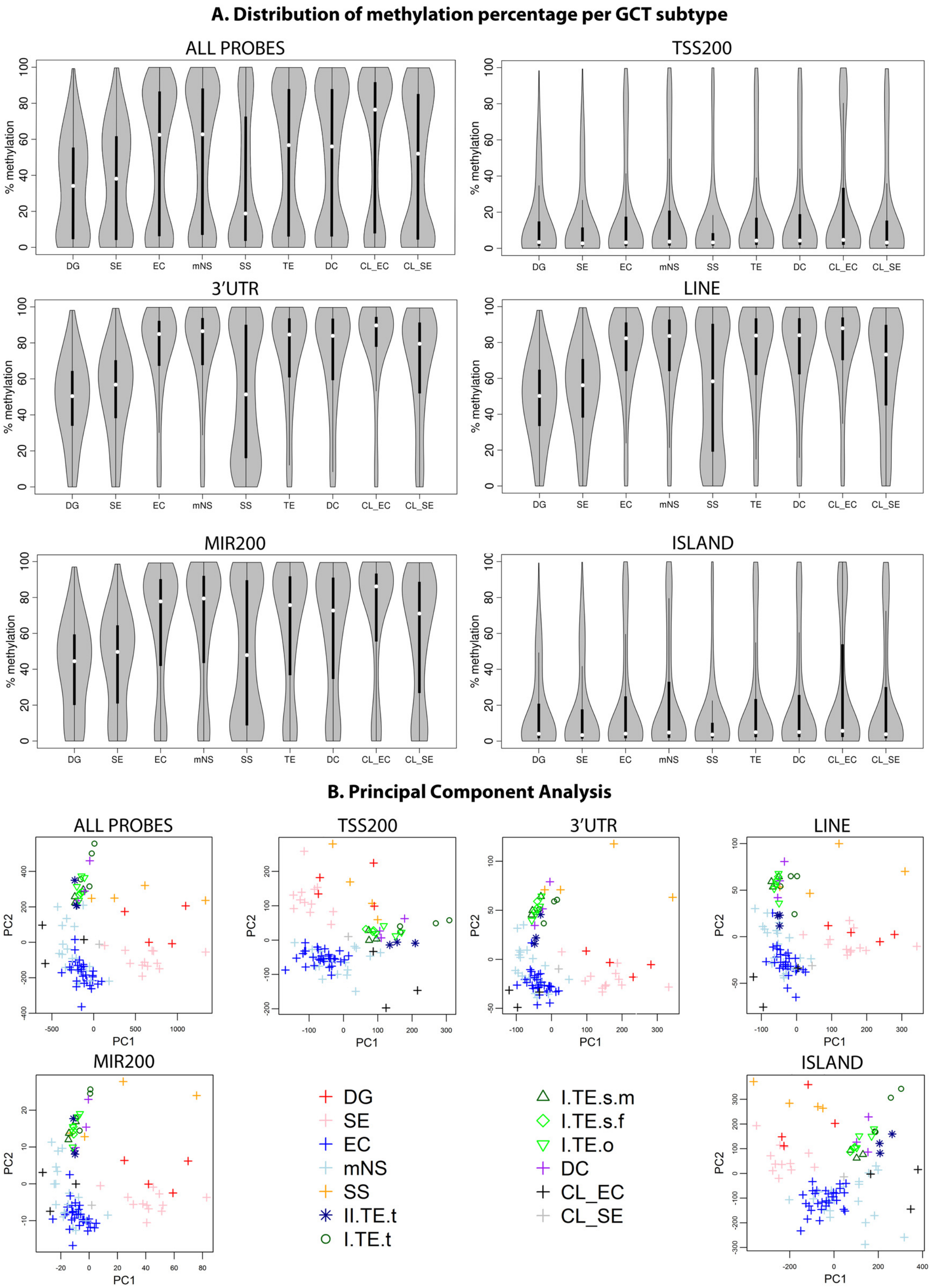

Fig 2. Methylation patterns in GCT subtypes and cell lines. To illustrate differences in methylation status between histological GCT subtypes two (visualization) methods were applied. Firstly, the methylation pattern over the whole genome and specific functional categories (Fig 1C) is visualized using the distribution of the methylation percentage β in all samples of a certain GCT subtype. Next, the discriminatory power of the methylation pattern for each individual sample is shown using principal component analysis. **(A) Distribution of methylation percentage.** Violin plots: grey areas indicate a kernel density plot of the methylation percentage (β) of all probes in all samples in a certain category. The boxplot indicates the interquartile range (black bars) and

median (white squares). X-axis labels indicate histological subgroup according to Fig 1A and 1B. TE indicates type I TE only. **(B) Principal Component Analysis.** The first two principal components (PC) are plotted to evaluate the discriminative power of the methylation pattern between the subtypes. Abbreviations of histological subtypes are explained in Fig 1A. CL indicates cell lines. Please note that in the legend of the PCA the TE group is subdivided based on gender and localization: I = type I; II = type II/formally part of the mNS group, s = sacrum, t = testis, o = ovary, m = male, f = female. A more detailed visualization of the TE classes is provided in S2 Fig, which also includes the full series of 18 functional categories, bootstrap validation of the PCA and an estimation of the variance explained by the first two principal components.

doi:10.1371/journal.pone.0122146.g002

using bisulfite sequencing in [63] (microRNA-371/2/3 cluster, Table 2). 719 gene symbols intersected between tumor and cell line DMRs (S3 Table). The major differences between the subgroups of GCT will be summarized hereafter.

Comparing SE/DG, EC/mNS and type I TE. Regardless of their presumed common origin, EC/mNS and SE/DG showed vastly different methylation profiles. The relative hypermethylation in EC/mNS versus SE/DG was concentrated in regions not involved in transcription regulation (Fig 3A). This pointed to a global difference in methylation status rather than differential methylation of specific regulatory elements. This also held for the hypermethylation of type I TE when compared to SE/DG (Fig 3B). The 61 DMPs hypermethylated in SE/DG relative to type I TE were concentrated at three specific genes: *NCOR2*, *ALOX12* and *ECEL1P2* (Table 1, S3 Table, S4A Fig).

DMPs between type I TE and EC/mNS indicated a more methylated profile of the EC/mNS group (Fig 3C). Moreover, the majority of the probes hypermethylated in type I TE were located on the X chromosome and can therefore be traced back to hemi-methylation of chromosome X in females (TE = male/female, EC/mNS = male only) (Table 1, S3B Fig). DMRs included many genes involved in male gametogenesis like *DMRT3* (Fig 4A). The EC marker *SOX2* [17,64] was present as one of the only 15 hypermethylated autosomal DMRs in type I TE (Fig 4B). These DMRs presumably relate to the cell of origin as well as to the sex of the patient (S4B Fig, Table 1 and S3 Table).

Type III (SS) versus type II seminomatous GCT (SE/DG). The general, probes significantly hypomethylated in SS as compared to SE/DG were enriched for regions associated with paternal expression (Fig 3D). DMRs hypermethylated in SE/DG predominantly included recurrent DMRs and DMRs within genes associated with germ cell and testis development (Table 1 and S3 Table). The promoter of *POU5F1* was relatively hypomethylated in SS, while it is a marker for the stem cell component of type II GCTs and not expressed in SS [17,46,65] (Fig 4C, discussed in Table 2). DMRs hypermethylated in SS also included genes associated with male germ cell determination, fertility and GCTs, enforcing the epigenetic relation between GCT cells and their cell of origin (Table 1 and S3 Table).

Specific GCT associated genes. A number of genes has been associated with (methylation in) GCTs, both regarding pathogenesis and diagnosis. Table 2 summarizes the literature for these genes and combines this with the methylation data from this study, e.g. overlap with DMRs and methylation profile of these genes (see also Fig 5 and S5A Table). A recent meta-analysis of GCT GWAS studies identified 19 SNPs associated with 13 genes [29]. For most genes their methylation profile was non discriminative between the GCT subtypes, the exceptions being *TEX14* which was also independently identified as a DMR[SE/DG-ss] (Fig 4D) and *BAX1*, which also contained a DMR[se/dg-SS] (all SNP related genes: S5B Table).

Imprinting status and X chromosome reactivation

As reviewed in the introduction, gradual and tightly controlled establishment of uniparental imprinting and X chromosome reactivation (female only) has been demonstrated in developing germ cells which is at least partly mirrored in their malignant counterparts. Regarding imprinting controlled regions (Fig 1C and S4 Table) in the tumor groups probes covering regions

Table 1. Pairwise comparison of GCT subtypes.

**A. Seminomatous (SE/DG) versus non-seminomatous (EC/mNS) GCTs**	
DMProbes[**SE/DG**-EC/mNS]	DMProbes[SE/DG-**EC/mNS**]
6	110,462 (25%)
[no enrichment]	↓ in tr and ↑ in non-tr, LINE/SINE suggested global difference in methylation status rather than differential methylation of specific regulatory elements. CpG islands were ↓. miRs regions were weakly ↑. ICRs were ↓, suggesting no difference in imprinting status.
DMRegions[**SE/DG**-EC/mNS]	DMRegions[SE/DG-**EC/mNS**]
0	*[global]*
-	*[global]*
**B. Seminomatous type II (SE/DG) versus type I (TE)**	
DMProbes[**SE/DG**-TE]	DMProbes[SE/DG-**TE**]
61	56,764 (13%)
significantly overrepresented on chromosome 12 (13/61) and preferentially located in the 5'UTR of genes and CpG islands	↓on tr, CpG islands and ICRs and ↑of non-trs, transposons and miRs (≈DMPs[SE/DG-**EC/mNS**]).
DMRegions[**SE/DG**-TE]	DMRegions[SE/DG-**TE**]
3	*[global]*
NCOR2: (*SMRT*, silencing mediator for retinoic acid and thyroid hormone receptors) nuclear receptor co-repressor on 12q24.31 involved in mouse spermatogenesis [47] and vitamin D metabolism in GCTs [48]. ***ALOX12:*** lipoxygenase family [49], has not implicated in GC(T) biology. ***ECEL1P2:*** increased methylation upon aging [50] → although not implicated in normal/aberrant germ cell development, this might explain the hypomethylation in pediatric type I TE as compared to adult type II SE/DG. All three genes were also DMR hotspots.	*[global]*
**C. Non-seminomatous type II (EC/mNS) versus type I (TE)**	
DMProbes[**EC/mNS**-TE]	DMProbes[EC/mNS-**TE**]
17,407	1,520
Enrichments were weak, the strongest being ↓ in transposons.	≈80% on X chromosome → differences in sex (TE = male+female, EC/mNS = male). ↑ in tr and ↓ in non-tr and transposon elements suggests differential methylation of specific regulatory elements. ↑ in CpG islands.
DMProbes[**EC/mNS**-TE]	DMRegions[EC/mNS-**TE**]
580 (all autosomal)	128 (15 autosomal)
e.g. ***DMRT3:*** implicated in testis development and male sex determination [46,51] ***MOV10L1:*** which has been implicated in human male infertility [52] and germ cell maturation in mice [53]. ***DDR2:*** crucial for spermatogenesis in mice [54] ICR_P ***WT1*** was also present.	e.g. ***SOX2:*** EC marker (see: Table 1). ***IRX5:*** germ cell migration in Xenopus laevis embryos; DMR ≈1kb downstream of its 3'UTR [55]. ***MSX1:*** progression of germ cells into meiosis, leading to germ cell maturation arrest in mutant embryos; DMR at its 3'UTR [56]. Hypomethylation in type II GCTs [57].
**D. Seminomatous type II (SE/DG) versus type III (SS)**	
DMProbes[**SE/DG**-SS]	DMProbes[SE/DG-**SS**]
15,340	2,830
↑ in non-tr and ↓in the tr. ↑ in ICR_P/IMPR_P200/1500 in line with paternal cell of origin of SS.	↓ at non-tr and CpG islands.
DMProbes[**SE/DG**-SS]	DMRegions[SE/DG-**SS**]
559	30
e.g. hotspot genes like ***NCOR2, ALOX12, ECL1P2, MSX1*** (see above). ***IRS2:*** associated with male germ cell and testis development [58]. ***POU5F1:*** SE/DG/EC marker (Table 1). ***TEX14:*** associated with known high risk GCT SNP [29].	***SERPINE1*** (plasminogen activator inhibitor 1, PAI-1): hypomethylated in GCT except in SS. PAI-1 SNPs have been associated with poor prognosis in GCTs [59]. The plasminogen activator system has been implicated in human infertility [60]. ***MOG:*** hypermethylated in SS, knockdown causes male germ cell differentiation in *mog* deficient C. Elegans [61,62].

This table concisely summarizes the results of the search for differentially methylated (DM) probes (P) and differentially methylated regions (R) between pairs (A and B) GCT subtypes. Briefly, the number of DMPs and DMRs is shown separately for probes hypermethylated in A or B. The subtype in which the probes are hypermethylated is indicated in bold and underlined. Also, a brief interpretation of the genomic function of the DMPs is provided. For the DMRs the associated genes are discussed in the context of GCTs. (Abbreviations) ↓ significantly underrepresented; ↑ significantly overrepresented; % DMPs is calculated relative to the total number of valid probes (Materials and Methods section). tr = transcription regulation associated regions (TSS200/TSSAssociated/5'UTR/EXON1); non-tr = non transcription regulation associated gene coding regions (GENE.BODY/3'UTR). The other functional categories are depicted in Fig 1C. [global] = global methylation difference between subtypes; no distinguishable potential subtype specific differentially methylated regulatory elements. (Associated sources) Statistical procedures are described in the Materials and methods section. The overall methylation pattern of each histological subtype is visualized in Fig 2. Functional enrichment of DMPs is visualized in Fig 3. Details of enrichment calculations and raw counts and percentages are presented in Table S2. Enrichment of chromosomes is depicted more detailed in S3B Fig. DMRs, recurrent tumor DMR and DMPs are listed in S3 and S1 Tables respectively. DMRs are visualized in Fig 4, S4 Fig and GSE58538: Files S1 and S2.

doi:10.1371/journal.pone.0122146.t001

that are regulating paternally expressed genes (ICR_P) showed somatic methylation in type I and II GCTs with a trend towards hypermethylation in DC (Fig 6A). SS and the cell lines showed hypomethylation of ICR_Ps, a distinction also visible in the PCA plots. In IMPR_P200/1500 the pattern of the ICR_P probes seemed to be pooled with a set of unmethylated probes (type I, II, IV GCT) presumably indicating contamination by non-imprinting related regions and hence not informative for imprinting status (S2A Fig, pages 15 and 16). A somatic methylation state was shown for ICR_M except in the SS (bimodal) and the CL_SE (hypomethylated); a difference corroborated by the separation of these groups in the PCA plot (Fig 6B). IMPR_M200/IMPR_P1500 probes showed hypomethylation similar to non-imprinted genes in all groups (S2A Fig, pages 18 and 19). No reactivation of chromosome X was seen in GCTs from female patients, which is reflected by the consistent 50% median methylation of the X chromosome in these cases (Fig 6C). The cell lines did not reflect the imprinting status of their *in vivo* counterpart, warranting caution when using the cell lines as a GCT model system in methylation based experiments.

Methylation status of ICR_Ps and ICR_Ms was similar between individual samples of the same histology (S2B Fig) with the exception of type I TE and DC (Fig 6D and S2B Fig). In addition to the earlier analysis, where the type II TEs were grouped with the mNS and the type I TEs were assessed as one class, TEs were also investigated individually, grouped according to sex and anatomical site, in line with sex specific imprinting occurring during fetal/germ cell development (Fig 6D). The genome-wide methylation pattern was similar for all TEs. No reactivation of chromosome X was seen in the GCTs from female patients. Sacral type I TEs showed somatic imprinting patterns both in males and females. In line with sex specific imprinting, ICR_P sites in testicular type I TEs were relatively hypomethylated compared to sacral TEs. In contrast, ovarian type I TEs showed a tendency towards hypermethylation. Of note, testicular type I TE also showed a trend towards hypomethylation in ICR_M (only 18 probes). On the other hand, the expected inverse pattern of ICR_P was seen in the ovarian TEs at the ICR_M sites. A pattern similar to ovarian type I TE was observed in the individual DC samples: heterogeneity and gradual deviation from biparental imprinting towards uniparental maternal imprinting. Two out of three type II TEs showed a somatic imprinting pattern of both ICR_P and ICR_M.

Validated ICRs (S4 Table) were also studied individually. After merging overlapping validated ICRs from literature, 28 unique ICRs remained of which 21 were covered by the 450K array (4 ICR_M, 16 ICR_P, 1 unknown). ICRs controlling the expression of *H19/IGF2*, *SNURF/SRPN* and *MEST* have been studied in GCTs previously (review & results in Table 2). In the ICR_Ps which constitute the majority of the validated ICRs, the dominating pattern is: (1) somatic methylation in the type II tumors (2) hypomethylation in the type I testicular TEs and SS and (3) a trend towards hypermethylation in DC and ovarian TE. (Fig 7A and 7B, S6 Fig).

In summary, ovarian type I TE and DC showed partial sex specific uniparental maternal imprinting, inverse of the uniparental paternal imprinting of SS. Testicular type I TE shows a trend towards erasure and type II GCTS (SE/DG/EC/mNS) showed somatic imprinting status.

Discussion

This study provides a detailed overview of the differences in global and local methylation status between type I-IV GCTs (Fig 1) and relates it to their cell of origin during normal germ cell development. Normal germ cell maturation includes complete de- and subsequent remethylation. Establishment of sex specific uniparental imprinting is physiological as is reactivation of chromosome X in female gametes. The largest methylation differences were detected between the hypermethylated EC/mNS + type I TE and hypomethylated SS + SE/DG clusters, in line with

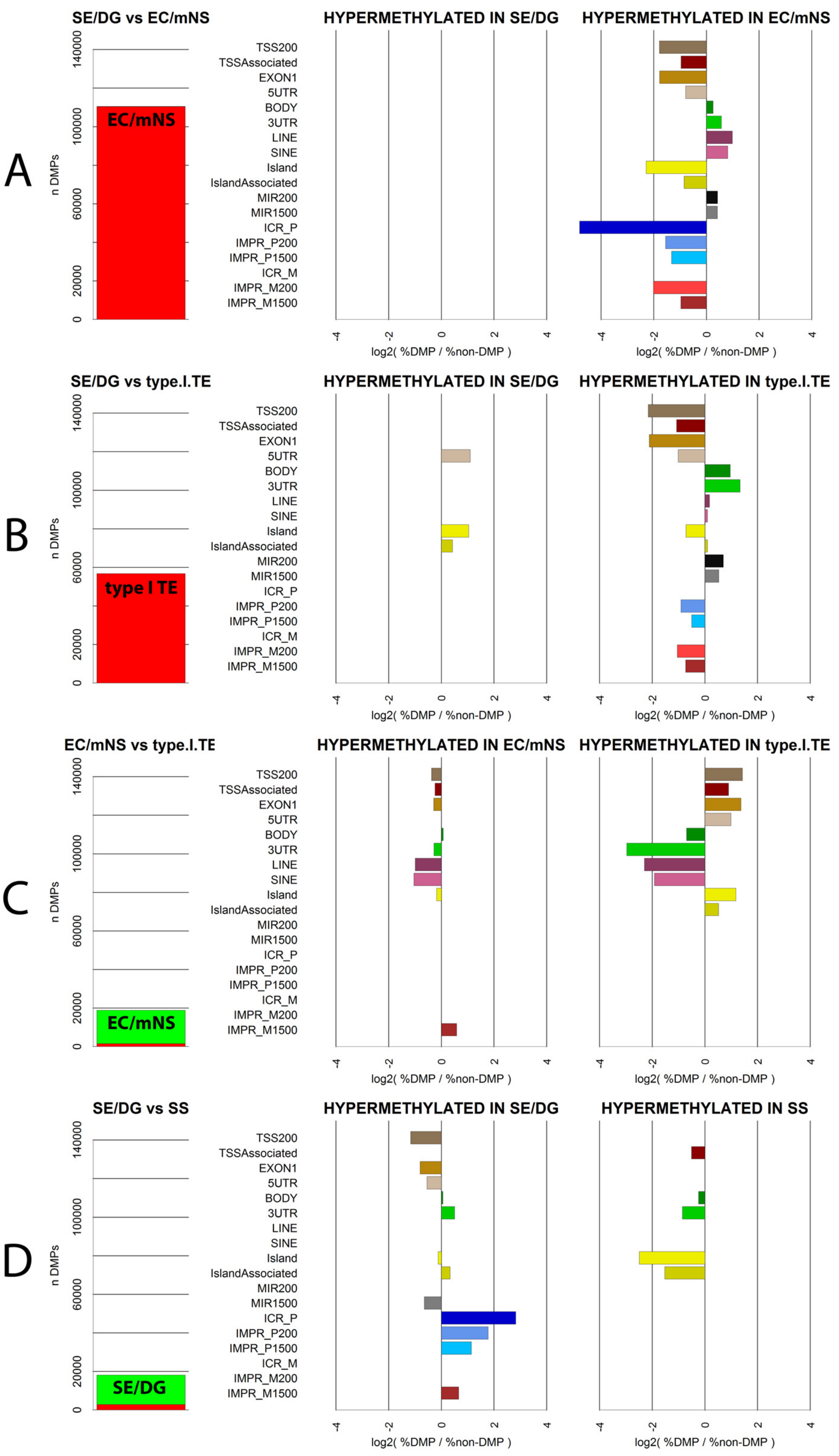

Fig 3. Functional enrichment of DMPs. DMPs were classified according to their functional genomic location (Fig 1C). Statistical over- and underrepresentation of probes in certain categories provides clues to differences between GCT subtypes in regarding function of methylation. Enrichment was assessed by comparing the number of probes in a functional category in a subset of DMPs with the that in the total dataset (Fisher's Exact test, see Materials & Methods section). Results are shown for four pairwise (A vs B)

comparisons of histological subtypes: **(A)** SE/DG versus EC/mNS; **(B)** SE/DG vs type I TE; **(C)** EC/MNS vs type I TE and **(D)** SE/DG vs SS. **(LEFT)** The number (n) of DMPs identified in either the DMP[**A**-B] (hypermethylated in A, green) or DMP[A-**B**] (hypermethylated in B, red) group. **(MIDDLE/RIGHT)** Functional enrichment in the DMP[**A**-B] and DMP[A-**B**] group respectively. X-axis: positive numbers indicate a significant overrepresentation of DMPs in a functional category compared to non-DMPs while negative numbers indicate a significant underrepresentation. Depicted is the log2 ratio of (1) the % of either DMP group assigned to a category and (2) the % of non-DMPs assigned to that category. Only significant enrichments are depicted (2-sided Fisher's Exact test, see Methods section for Bonferroni corrected α threshold). DMPs[SE/DGvs**SS**].IMPR_P1500 showed significant underrepresentation, but could not be plotted on log scale (0 probes in DMP group). Details of calculations and raw counts and percentages are presented in S2 Table. Y-axis: functional categories as specified in Fig 1C.

doi:10.1371/journal.pone.0122146.g003

previous reports [14,43,117,135] (Fig 2A). However, the methylation profiles also allowed for a more detailed separation of EC/mNS, SE/DG, TE/DC and SS clusters, which is in line with the differentiation status of the tumors and their cell of origin. This distinction was also apparent when specific functional genomic regions were evaluated (Fig 2B). Hypermethylation in EC/mNS and type I TE is concentrated at non-transcription related regions when compared to SE/DG, pointing to a global difference in methylation status rather than differential methylation of specific regulatory elements. Moreover, EC/mNS is somewhat more methylated than type I TE and shows specific differences at transcription regulating genomic regions including genes implicated in male germ cell development. Regarding type III tumors, differential hypomethylation in SS relative to SE/DG is enriched for paternally expressed imprinting associated regions and DMRs cover male germ cell related genes (Figs 3, 4 and 5, Tables 1 and 2). In addition, marked differences in imprinting status were observed. Ovarian type I TE and DC showed partial uniparental maternal imprinting, inverse of the uniparental paternal imprinting of SS. Testicular type I TE shows a trend towards imprinting erasure and type II GCTS (SE/DG/EC/mNS) showed somatic imprinting status (Figs 6 and 7). The local and global methylation difference observed between GCTs could be matched to physiological germ cell development, but did not match with their respective cell line models (Fig 8).

Limited knowledge exists about the progenitor of type I tumors. The absence of CIS and clinically different presentation (pediatric, frequently extra-gonadal, fully differentiated histology: TE/YST) sets them apart from the type II tumors [16–18]. Their bimodal global methylation status could a pattern generally observed in normal differentiated tissues and in very early germ cell progenitors (pre-migration. Historically type I and II tumors are also thought to be different with regard to their imprinting status. Imprinting status in these tumors was earlier shown to be somatic (biparental) or partially erased in case of the type I tumors and erased in case of the type II GCTs [16]. This positions the progenitor cell of type I tumors before imprinting erasure in the gonad. Indeed biparental (somatic) imprinting status in extra-gonadal TE was confirmed in this study and by Amatruda and colleagues [20]. There is a trend towards imprinting erasure in testicular type I TE. Ovarian type I TE show a trend towards completely maternal imprinting, but starting from a biparental status (50%), not showing any evidence of prior complete erasure (Fig 6D). This (partial) mimicking of female germ cells in ovarian type I TE is in line with in several studies [20,131,132]). However, the non-erased imprinting status, inactivated X chromosome and generally methylated state fits with the cell of origin at the very early PGC stage, which is then blocked in physiological complete demethylation, erasure and X reactivation and, when subjected to a gonadal micro-environment, shows partial erasure/uniparental imprinting [16–18] (Fig 8).

Most data is available on the epigenetic constitution of the type II tumors, as reviewed before [13,21]. A strongly hypomethylated state was recently shown for all CIS, the common precursor of SE and EC [136]. Earlier studies have suggested separated NS-CIS and a SE-CIS types

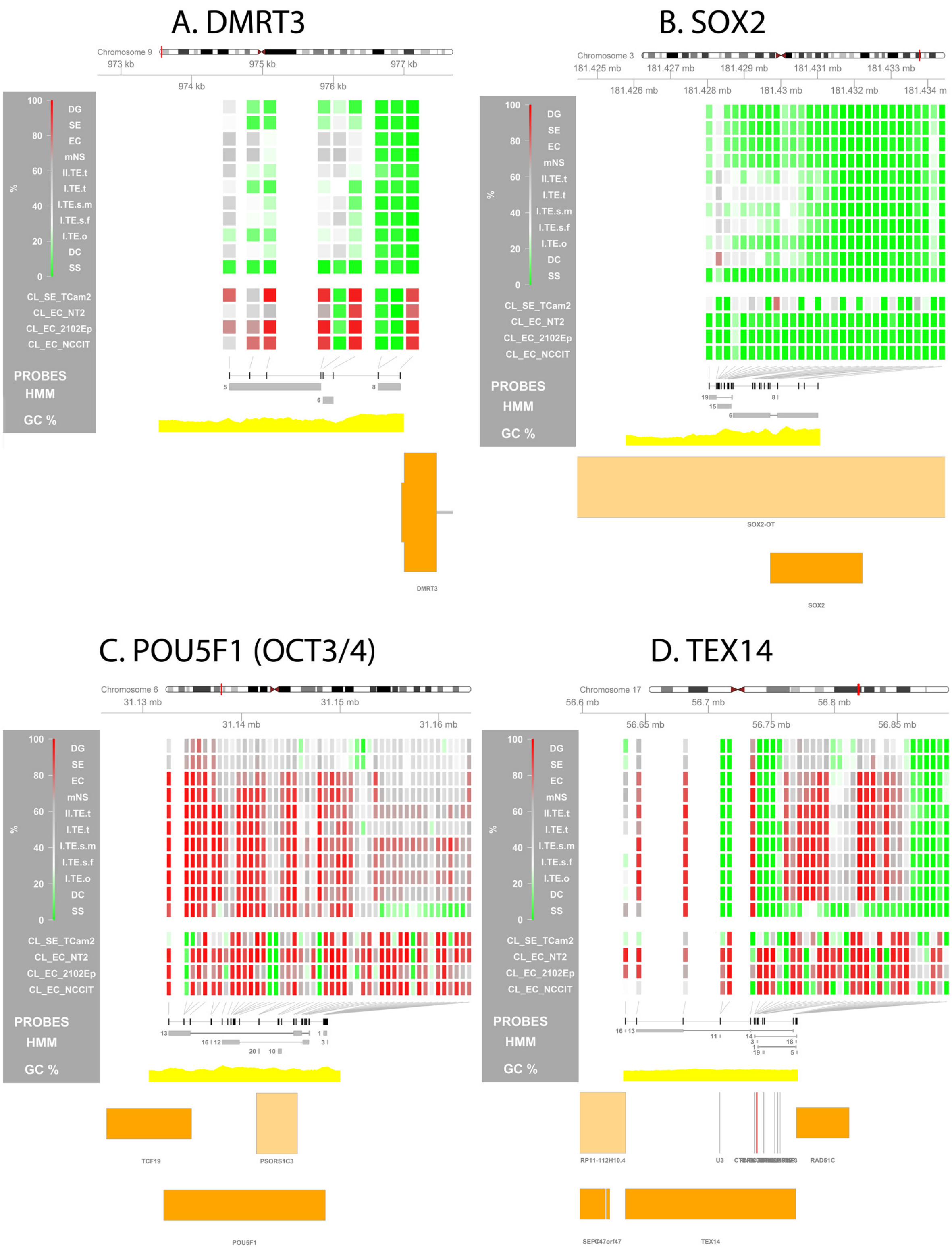

Fig 4. Methylation profile at GCT subtype specific differentially methylated regions (DMRs). Visualization of the methylation percentage at specific loci is used to zoom in on a predefined region and investigate local methylation differences between GCT subtypes. **(A) *DMRT3*, (B) *SOX2*, (C) *POU5F1* (*OCT3/4*), (D) *TEX14*. (Visualizations)** From top to bottom the following is depicted: (1) Four-color heat map indicating methylation % for each individual probe in the depicted region. For the sample groups specified on the left the median methylation % is shown. (2) Position of all probes in the region of interest (ROI) is annotated as black rectangles. (3) HMM segments are displayed as grey boxes spanning the segment's width and grouped per state. Numbers indicate the

state of each (group of) segment(s). (5) GC% was obtained from the UCSC genome browser database (gc5Base table). (6) Transcripts overlapping with the ROI are plotted at the bottom. Plot generated using the Gviz package. Abbreviations of histological subtypes are explained in Fig 1A. Please note that the TE group is subdivided based on gender and localization: I = type I; II = type II/formally part of the mNS group, s = sacrum, t = testis, o = ovary, m = male, f = female. CL indicates cell lines.

doi:10.1371/journal.pone.0122146.g004

[135], but the lack of methylation in CIS combined with absence of SOX2 (EC marker) expression [64,136,137] increases the likelihood of a single precursor and progression into SE or NS. The CIS-like state is evident in the hypomethylated profile of SE/DG as shown in this article and previous research [14,43,117,135,136]. EC and mNS show a (*de novo*) methylated profile (Fig 2A). This is in line with the previously reviewed increased methylation in the transition of CIS into NS [13,14,43,138], possibly illustrating reversal to a hypermethylated ES like state [7,16,139–142] or a bimodal methylation state normally present in differentiated tissues as shown in the differentiated NS. The consistent somatic imprinting pattern in general and at specific ICRs (Fig 6, S6 Fig and S4 Table) was in line with an earlier report [20] but contrasted with targeted studies suggesting erased imprinting status at specific ICRs in these tumors using mainly indirect methods (allele specific expression analysis) and or non-quantitative methylation analysis (bisulfite specific restriction enzymes) (for review Table 2). The hypomethylated progenitor and somatic imprinting pattern (Fig 6A and 6B) situates the cell of origin of the type II tumors possibly earlier than previously described [16]: after global demethylation but before imprinting erasement, which is also in line with the occurrence of extra-gonadal type II GCTs (brain, anterior mediastinum) and their totipotent, embryonic stem cell like potential [16,139–142] (Fig 8).

The other GCT subtypes are historically hypothesized to originate from more mature germ cell progenitors. Their marker profile has placed the type III tumors at the pre-spermatogonium state with regard to their cell of origin [36–39,46]. Earlier epigenetic data showed a heterogeneous profile of histone modification and methylation profiles, not corresponding with a pre-spermatogonial origin [143]. Our limited series of SS show a consistent pattern of distinct hypomethylation and loss of imprinting at the paternally expressed ICRs (ICR_M: heterogeneous $\geq$ 50%, Fig 2B). This matches with a cell of origin between the gonocyte and spermatogonium stage, after establishment of uniparental imprinting but before initiation of *de novo* methylation. The type IV tumors (DC) show a pattern comparable to other differentiated tissues (ovarian type I TE) and show a general trend towards uniparental maternal imprinting but not starting from a completely erased state, potentially placing their cell of origin and pathogenesis parallel to the type I ovarian TE and not as a separate entity originating from a completely maternally imprinted an differentiated female germ cell as described before [16] (Figs 2B, 6 and 8).

In conclusion this exploratory study of genome wide methylation profiles of GCT subtypes identified specific and global methylation differences, providing novel insight into the developmental timing and underlying biology of the various subtypes of GCTs and their (embryonic) cells of origin (Fig 8). Methylation profiles allowed for separation of clusters of NS, SE/DG, SS and TE/DC, largely in line with the current WHO classification. SE/DG/SS were globally hypomethylated, in line with previous reports and the demethylated state of their precursor. Differential methylation between subtypes reflected the presumed cell of origin as did imprinting status. However, somatic imprinting in type II GCT might indicate a cell of origin after global demethylation but before imprinting erasure. This is earlier than previously described, but agrees with the totipotent/embryonic stem cell like potential of type II GCTs and their rare extra-gonadal localization. The results support the common origin of the type I TEs and show strong similarity between ovarian type I TE and DC. However, the limited samples size and

Table 2. GCT (methylation) associated genes.

Gene & region	Description
APC, chr5 (+), 112,043,195–112,181,936	**GCT link:** A single study with small sample size (n = 10) showed increase methylation in most YST as compared to germ cells in normal testis. Expression was high in germ cells and low in most YSTs [66]. **Findings:** 2102EP showed mild but significant relative hypermethylation compared to the other cell lines, but for all tumor groups *APC* was consistently hypomethylated.
AR, chr X (+), 66,763,874–66,950,461	**GCT link:** Androgen receptor methylation can be used as a readout for X inactivation in non-germ cells. *AR* was methylated in differentiated NS, but unmethylated in a proportion of ECs and all SE & SS. This supports the hypothesis that methylation does not occur in the germ cell lineage [67]. **Findings:** the promoter region of the *AR* was completely deprived of methylation in all male tumors while a certain amount of methylation (ca. 50%) was present in the female samples. *AR* contained a DMR only in the CL where it was relatively methylated in NT2 as compared to all other cell lines (Fig 5A).
**CTA genes**	**GCT link:** Cancer Testis Antigens (CTA) are primarily unmethylated in SE. *MAGEA1/3* are predominantly methylated in NS while *SYCP1* is unmethylated in NS [68]. *MAGEA1*: chrX:152,481,522–152,486,116(-), *MAGEA3*: chrX:151,934,652–151,938,240(-), *SYCP1*: chr1:115,397,424–115,537,991(+). **Findings:** Methylation differences in these genes were not remarkable except for differential hypomethylation of TCam-2 and NCCIT compared to the other cell lines. The TSS associated regions of *MAGEA1* and *SYCP1* were consistently hypomethylated in SS.
GATA4, chr 8 (+), 11,534,468–11,617,511	**GCT link:** previously identified DMR between TCam-2 and NCCIT, promoter region hypermethylated in TCam-2 [69]. **Findings:** The *GATA4* promoter was differentially hypomethylated in all CL_EC as compared to EC_SE, but this was exactly opposite in the SE and EC tumor samples. Testicular TEs, like EC/mNS samples showed relative hypermethylation while sacral/ovarian TEs, DCs and SS showed relative hypomethylation like the SE/DG samples.
HIC1, chr17 (+), 1,957,448–1,962,981	**GCT link:** 55% of the GCT show methylation of this area which shows frequent loss of heterozygosity in somatic adult cancers. 5AZA treatment strongly induced *HIC1* expression in non-GCT CLs [70]. *HIC1* promoter methylation has been implicated in treatment resistance in GCTs [71]. **Findings:** HIC1 was showed predominantly hypomethylation in all GCT subtypes even though a weak DMR[**EC/mNS**-TE] was identified. Of the cell lines, only 2102EP showed differential hypermethylation.
KIT, chr4 (+), 55,524,085–55,606,881 / ***KITL***, chr 12 (-), 88,886,570–88,974,628	**GCT link:** KIT and KITL regulate primordial germ cell development and homing to the gonad [72–76]. In the embryonic phase the guidance of KIT+ primordial germ cells from the hind gut epithelium to the gonads depends strongly on KITL mediated chemo attraction [75,77–79]. In the postnatal testis KIT-KITL signaling takes place via paracrine signaling in the germline stem cell niche and is crucial for spermatogenesis from the spre-matogonial stage onwards [73,75,80,81]. More mature mouse spermatids and spermatozoa express a c-terminal truncated form of KIT transcribed from an intronic promotor [82]. Mechanistically, constitutive paracrine / autocrine activation of KIT/KITL signaling is implicated to be a crucial initiating event for the malignant transformation of maturation arrested germ cell progenitors [17,19,22]. In the early stages, KITL positivity is a hallmark of maturation arrested germ cells, CIS and intratubular SE [17,83–85]. Progression into invasive SE is also strongly related to KIT/KITL signaling while much less association with the NS phenotype has been shown [80,86–89]. Activating *KIT* mutations are identified in ca 13–60% of the SE (rare in NS) and result in constitutive kinase activity because of ligand independent dimerization and phosphorylation [90–93]. Recent GWAS studies identified susceptibility loci for GCTs close to, within or directly related to GCTs [29,94–105]. No information about *KIT* or *KITL* methylation in tumors was presented in literature although *KITL* promoter methylation was significantly lower in blood of these patients [106] and SNPs in KITL combined with aberrations in cAMP regulation were suggested to contribute to tumor risk in these patients [105]. **Findings:** *KIT* (S6A Fig) and *KITL* (S6B Fig) were not differentially methylated between any of the tumor groups or cell lines.

(*Continued*)

Table 2. (*Continued*)

Gene & region	Description
miR-371/2/3, chr19 (+): ***(371)*** 54,290,929–54,290,995 / ***(372)*** 54,290,995–54,291,210 / ***(373)*** 54,291,959–54,292,027	**GCT link:** The *micro-371-2-3* cluster is expressed in the stem cell component of GCT [107] and is a potential diagnostic serum marker for GCT [108]. Upstream of the TSS of this cluster a DMR has been identified between TCam-2 and NCCIT [69]. Differential methylation in GCT cell lines has been validated using pyrosequencing and the methylation level showed significant and strong inverse correlation with the expression of miR-373 (Spearman's ρ -0.90, p = 0.037) [63]. **Findings:** The *miR-371-2-3* cluster was hypomethylated in TCam-2 (CL_SE) and 2102EP and hypermethylated in NT2 and NCCIT (Fig 5B). However, with the exception of SS the tumors showed hypermethylation of this region, despite known expression in the stem cell components of type II tumors [63,107].
NANOG, chr12 (+), 7,940,390–7,948,655	**GCT link:**Specific marker for the all stem cell components of GCTs [17]. RA treatment of NT2 cells also increased methylation here [109]. Analogous to this CpG sites in the *NANOG* promoter (0–306 bp upstream of the TSS) were found hypomethylated in spermatogonia and hypermethylated in sperm [110]. **Findings:** The *NANOG* promoter region showed a trend towards relative hypomethylation in the undifferentiated stem cell components of the type II tumors as compared to all other (more differentiated) GCT subtypes including the type II TE and mNS (intermediate status). However, The number of probes and consistency of the difference lacked significance (Fig 5C).
***POU5F1* (*OCT3/4*)**, chr6 (-), 31,132,114–31,148,508	**GCT link:** Specific marker for the all stem cell components of GCTs [17,65,111]. *OCT3/4* transcription is regulated by methylation of conserved regions up to 2.6kb upstream of the TSS. Another study also showed that little increase of methylation at specific sites upstream of *OCT3/4* strongly inhibited expression [109,112,113]. Differentiation of NT2 after retinoic acid treatment resulted in increased methylation and loss of expression [109].**Findings:** A promoter DMR[**SE/DG**-SS] was identified despite the fact the SE/DG express the OCT3/4 protein and SS do not [17,46,65] (Fig 4C). However, probes located close to its transcription start site are generally methylated between 20 and 40% in OCT3/4 positive tumors (SE/EC) which results in unmethylated alleles primed for expression. Moreover, the promoter region of *OCT3/4* showed a non-significant trend towards lower methylation levels in SE/DG and EC/mNS when compared to the differentiated tumors (TE). Most importantly however, regulation of *OCT3/4* expression is (also) crucially influenced by specific sites more upstream (ca. 2.6 kb) and a set of distant enhancer [112,113]. Also, we previously showed that even though high promoter methylation is generally associated with low expression, this is not always the case [69].
PRSS21, chr16 (+), 2,867,164–2,876,305	**GCT link:** *TESTISIN* (PRSS21) is a proposed tumor suppressor gene in TGCT regulated by methylation of a 385bp long CpG rich island [114] and CpG sites close to the TSS [115]. **Findings:** Al GCT subtypes except SS (DMR[**SE/DG**-SS]) showed hypermethylation of *PRSS21*.
RUNX3, chr1 (-), 25,226,002–25,291,612	**GCT link:** 90% of the infantile YSTs (type I) showed methylation of *RUNX3* while methylation was only rarely observed in the adult GCTs [57,116,117]. **Findings:** The promoter region of *RUNX3* was consistently hypomentylated, progressing to hemimethylation on larger distances from the TSS (except SS). *RUNX3* only showed differential methylation between the cell lines, most consistently showing hypomethylation in NCCIT and hypermethylation in 2102EP.
SOX17, chr8 (+), 55,370,495–55,373,456	**GCT link:** Discriminative marker between EC (+) and SE (-) [17,64]. **Findings:** *SOX17* was consistently hypomethylated in all tumor groups and cell lines (Fig 5D).
SOX2, chr3 (+), 181,429,712–181,432,224	**GCT link:** Discriminative marker between EC (+) and SE (-) [17,64]. Previously identified DMR upstream of TSS between (≈50%) TCam-2 and (≈0%) NCCIT [69] ≈1kb upstream of the *SOX2* TSS. The region directly upstream of the *SOX2* TSS has consistently been found hypomethylated in both cell lines [69,118]. TCam-2 has been shown to differentiate and become *SOX2* positive after extra-gonadal injection in mice [119]. **Findings:** A region ≈1 kb upstream of the *SOX2* TSS was differentially hypomethylated in all CL_ECs as compared to TCam-2 (GSE58538: File S2). EC and SE tumor samples showed consistent hypomethylation of the region -154 –-2283bp upstream of the *SOX2* TSS in contrast to the TE samples which showed higher levels of methylation (DMR[EC/MNS-**TE**], Fig 4B).

(*Continued*)

Table 2. (*Continued*)

Gene & region	Description
***TFAP2C* (*AP-2γ*)**, chr20 (+), 55,204,358–55,214,339 / ***TFAP2A* (*AP-2α*),** chr 6 (-), 10,393,419–10,419,892	**GCT link:***AP-2γ* is crucial for progression of PGCs into the germ line [120]. It is a known germ cell marker, abundantly expressed in CIS and SE, and heterogeneously expressed in NS and somatic tumors [120,121]. *AP-2γ* expression is induced by estrogens [122]. Epigenetically, ChIP-seq analysis targeting activating histone marks showed strong enrichment of *AP-2α* and *AP-2γ* motifs in the SE-like cell line TCam-2 [69]. **Findings:** *TFAP2A* showed mostly hypomethylation in all tumor groups and cell lines. Only NCCIT was showed significantly increased methylation at the gene coding region compared to the other cell lines (GSE58538: File S2). All TE samples showed a non-significant block of hemimethylated probes close to the TSS of *TFAP2A*. *TFAP2C* was consistently hypomethylated in all tumor groups and cell lines.
XIST, chrX (-), 73,040,486–73,072,588	**GCT link:** *XIST* is completely methylated in male somatic cells, in contrast to female somatic cells. Testicular GCTs show hypomethylation of the 5' end of *XIST* which, have been suggested for TGCT diagnostics [123] but has so far not been validated. SE/NS/SS showed *XIST* expression (X inactivation) [67]. **Findings:** *XIST* showed no significant differential methylation in the comparison of the tumor groups or cell lines. Female gonadal tumors, SE and SS showed a trend towards less methylation as compared to the strongly methylated profile of the non-seminomatous tumors and male type I TE.
**ICR_M: *H19-IGF2***, chr11, 2,020,834–2,023,499	**GCT link:** *H19* (M expressed) and *IGF2* (P expressed) are inversely controlled by this ICR upstream of *H19* [124]. In mice oocytes are erased at *H19* before meiosis while bialelic methylation occurs before the gonocyte stage in males [125]. In humans *H19* is erased in fetal spermatogonia, but becomes fully methylated before meisosis (spematogonia) [126]. *H19* erasure fis unctionally illustrated in [127] and related to pluripotency markers (SOX2 and OCT3/4) in germ cell development in [128]. Previous studies using have suggested low methylation of the *H19-IFG2* ICR in a variable, but generally high percentage of the type II GCTs. This has generally been interpreted as imprinting erasure. Somatic imprinting has been shown in non-gonadal TE and mimicking of female germ cells has been seen in ovarian TE. Most studies investigated imprinting indirectly using allele specific expression limiting the sample sizes because of the mandatory presence of SNPs in this analysis to be informative [129–132]. But a number of studies inquired the DNA methylation status directly using bisulfite restriction analysis, identifying consistent demethylation of one allele and variable methylation of the other in allele specific analysis and low, but not absent methylation in non-specific analysis [124,133]. Low-somatic imprinting in DG was also shown by Amatruda and coworkers in a high throughput approach [20]. **Findings:** The SS in our series show complete methylation at 1 of the two *H19*/*IFG2* sites indicating a paternal committed origin. The sacral TEs exhibit mainly a somatic pattern, presumably indicating a pre-erasure origin. The gonadal I TE/DC show the lowest level of methylation presumably representing (partial) erasure (I.TE.m.t, TE) or complete maternal imprinting (I.TE.f.o, DC). Type II GCTs were found to consistently show somatic imprinting (Fig 7B; 2 regions from literature: S4 Table).
**ICR_P: *SNURF/SNRPN***, chr15, 25,199,934–25,200,343	**GCT link:** *SNURF/SNRPN* has been described to show derivation from somatic imprinting in type II GCTs (non-quantitative, not necessarily indicating erasure) [134]. Low, but not absent methylation in non-allele-specific analysis [124]. Schneider and colleagues showed absence of the methylated band in bisulfite restriction analysis in 9 dysgerminomas [131]. **Findings:** In this dataset, this *SNURF/SNRPN* (controlling paternal expression) was only covered by a single probe (S6 Fig). This very limited evidence suggests somatic imprinting in the type II tumors and sacral TE and uniparental status in the other subtypes: loss of imprinting in the I.TE.m.t and complete methylation in the ovarian tumors (DC, I.TE.f.o).
**ICR_P: *MEST***, chr7, 130,130,740–130,133,111	**GCT link:** The *MEST* ICR regulates paternal expression, is already erased in fetal spermatogonia and remains so during male germ cell development [126]. **Findings:** The imprinting during germ cell development is reflected in our findings: (1) hypomethylation in the testicular type I TE and SS, (2) somatic imprinting in the type II tumors, (3) somatic-high imprinting in the ovarian and sacral TE, (4) high methylation in DC (Fig 7A).

Genomic locations and strand were retrieved from genecards.com/UCSC. Detailed visualizations of the methylation status of these genes is presented in Fig 5 and S5A Fig. DMRs in the cell lines are presented in GSE58538: File S2. ICRs are visualized in Fig 7 and S6 Fig.

doi:10.1371/journal.pone.0122146.t002

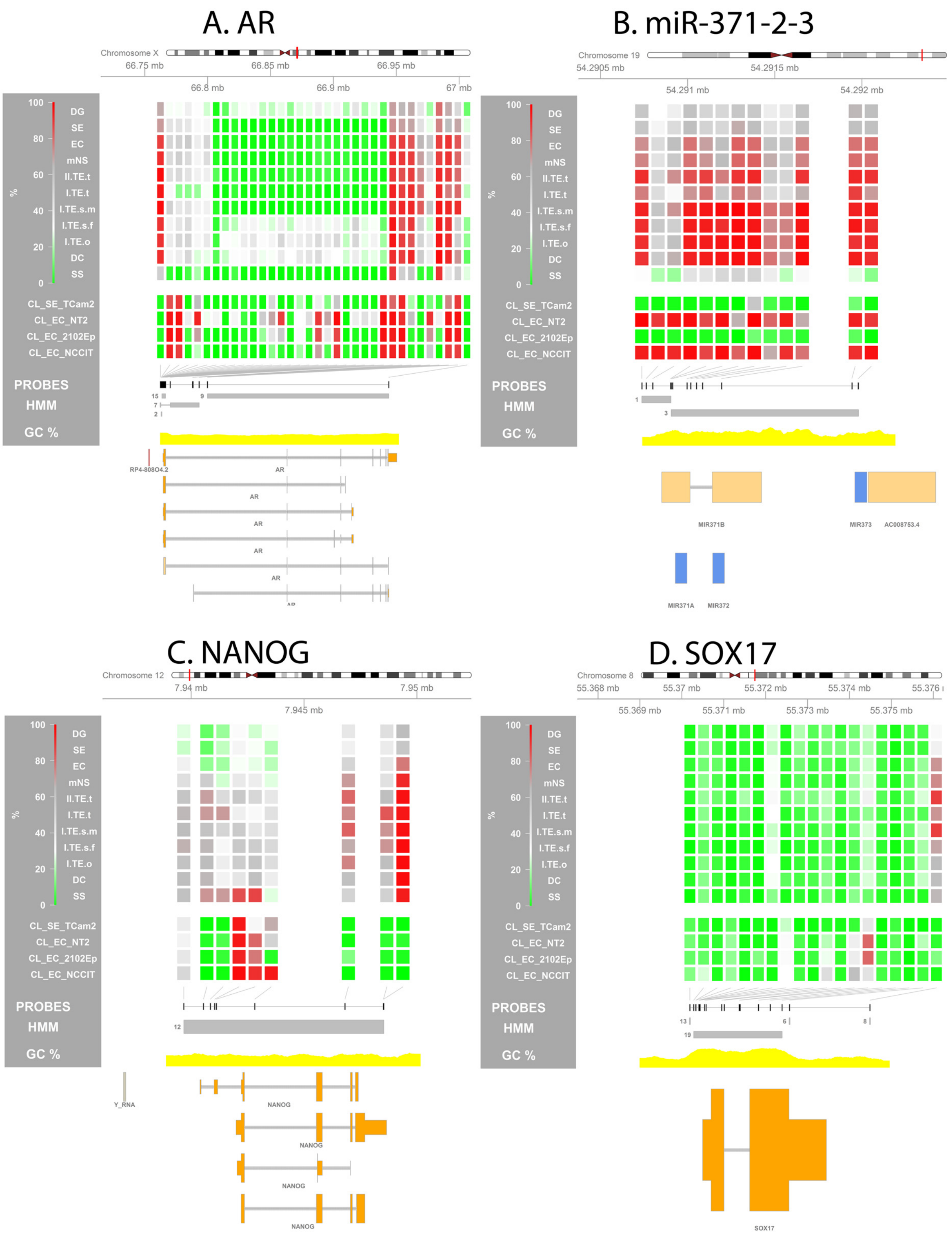

Fig 5. Methylation profile of GCT specific genes and regions of interest (ROIs). Visualization of the methylation percentage at specific loci is used to zoom in on a predefined region and investigate local methylation differences between GCT subtypes. The genes are reviewed in Table 2. **(A)** ***AR*****, (B)** ***miR-371-2-3*****, (C)** ***NANOG*****, (D)** ***SOX17*****. (Visualizations)** From top to bottom the following is depicted: (1) Four-color heat map indicating methylation % for each individual probe in the depicted region. For the sample groups specified on the left the median methylation % is shown. (2) Position of all probes in the region of interest (ROI) is annotated as black rectangles. (3) HMM segments are displayed as grey boxes spanning the segment's width and grouped per state.

Numbers indicate the state of each (group of) segment(s). (5) GC% was obtained from the UCSC genome browser database (gc5Base table). (6) Transcripts overlapping with the ROI are plotted at the bottom. Plot generated using the Gviz package. Abbreviations of histological subtypes are explained in Fig 1A. Please note that the TE group is subdivided based on gender and localization: I = type I; II = type II/formally part of the mNS group, s = sacrum, t = testis, o = ovary, m = male, f = female. CL indicates cell lines.

doi:10.1371/journal.pone.0122146.g005

conflicting results with some of the current literature warrants careful interpretation of the results and validation in a larger/extended dataset. Moreover, to interpret the function of differential methylation between GCT subtypes, targeted validation the findings using matched expression data or careful evaluation of the effects of methylation in cell line models of GCTs is a crucial next step, even though validation of a biological relevant and representative DMR in *microRNA-371/2/3* (Table 2) showed excellent match with the results of bisulfite sequencing. The in-depth review of related literature and extensive accompanying online data (supplementary and on GEO) serve as a hypothesis generating source for future research.

Materials and Methods

Samples

Patient samples. Use of tissue samples remaining after diagnosis for scientific reasons was approved by Medical Ethical Committee (MEC) of the Erasmus MC Rotterdam (The Netherlands), permission 02.981. This included the permission to use the secondary tissue without further consent. Samples were used according to the "Code for Proper Secondary Use of Human Tissue in The Netherlands" developed by the Dutch Federation of Medical Scientific Societies (FMWV (Version 2002, update 2011)). An overview of the samples in this study is presented in Fig 1A and 1B. Samples were collected when submitted to the pathology department and stored in liquid nitrogen.

Cell lines. Four cell lines were included (Fig 1B), all modelling type II GCTs. Cell lines derived from EC (CL_EC) include NT2[144–148], NCCIT [145,149] and 2102EP[144–148]. TCam-2 closely resembles SE (CL_SE) [150–152]. TCam-2 was grown in RPMI1640 (#61870–010, Thermo Fisher Scientific / Life Technologies, Carlsbad, CA, USA). 2102EP and NCCIT were grown in DMEM/F12 (#12634–010, Thermo Fisher Scientific / Life Technologies). NT2 was grown in DMEM high glucose (#31966–021, Thermo Fisher Scientific / Life Technologies). All cell lines were cultured in T75 cm^2 flasks to 75–90% confluence, each with the addition of 10% Fetal Calf Serum (#CH30160.03, FCS, GE Healthcare Life Sciences, HyClone Laboratories, Utah, USA) and 1/100 Penicillin/Streptomycin (#15140, Thermo Fisher Scientific / Life Technologies).

Methylation profiling

DNA was isolated as described in [110]. The GCT material used contained > 75% tumor cells. Bisulfite conversion and methylation detection was performed using Illumina's HumanMethylation450 BeadChip (450K array) and exported as described in [69]. This array does not distinguish between DNA methylation variants like 5mC and 5hmC [153].

Data analysis

Data (pre-)processing. Further processing was carried out in R using the LUMI package [154] according to [155,156]. In the raw data, no structural differences in quality or batch effects were observed. Poorly performing probes (detection $p<0.01$ in > 95% of the samples), cross hybridizing probes and probes with a SNP at or within 10 bp of the target CpG (allele frequency > = 0.05) were excluded [156]. As a result 44,540 probes were discarded, leaving 437,881 valid, methylation related probes for processing and analysis. Finally, color

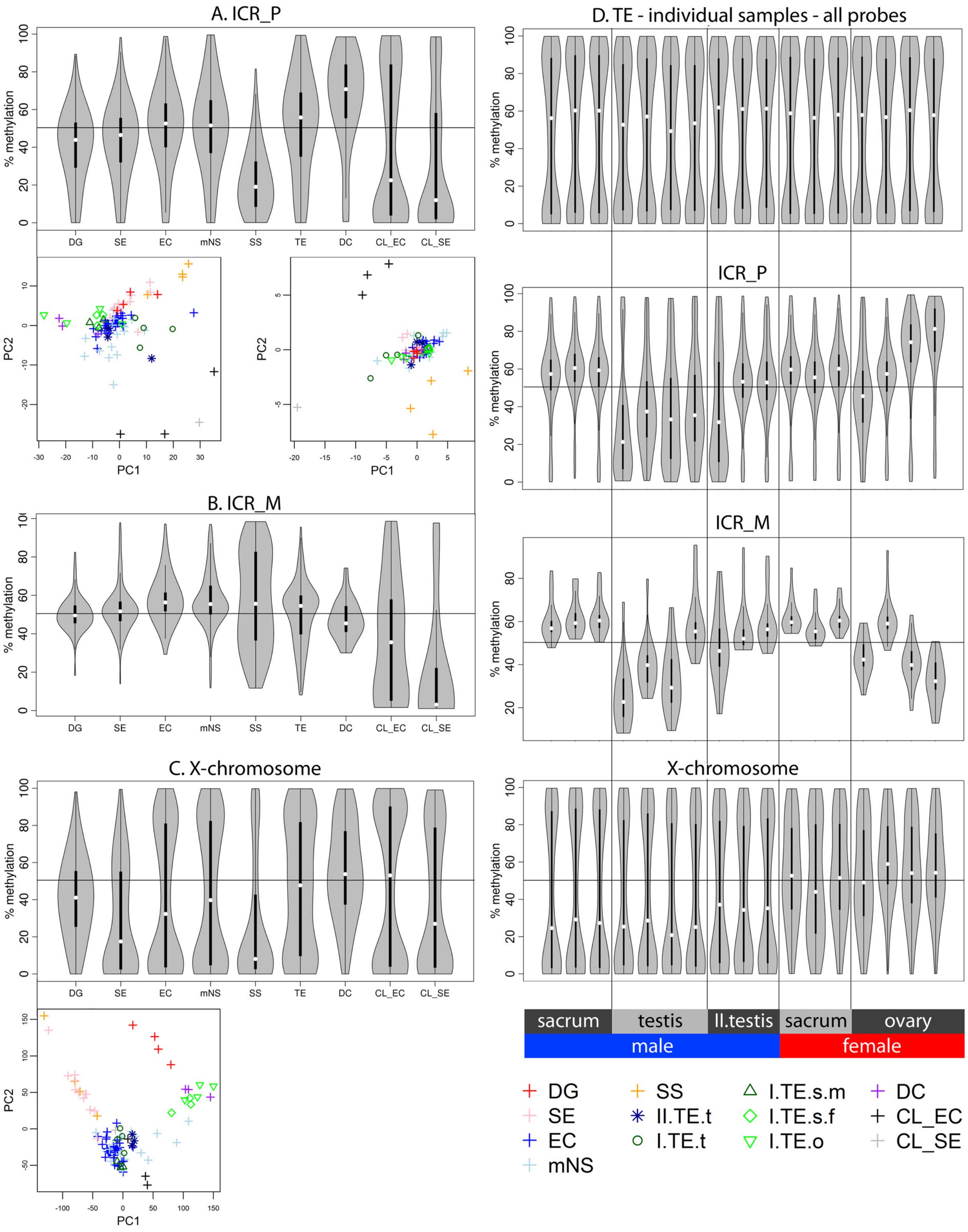

Fig 6. Methylation of imprinting control regions and the X chromosome. Analogous to Fig 2 the differences in methylation status between histological GCT subtypes is illustrated by two methods. Firstly, the methylation pattern is visualized using the distribution of the methylation percentage β. Next, the discriminatory power of the methylation pattern for each individual sample is shown using principal component analysis. **(A)** All probes associated with paternally expressed genes (ICR_P). **(B)** All probes associated with maternally expressed genes (ICR_M). **(C)** All probes located on the X chromosome. **(D)** Distribution of methylation in individual TE samples ordered by sex and localization. To compare type I and II TE the n = 3 type II pure TEs from the mNS

were included in this visualization. Methylation levels of all probes, and probes associated with ICRs (P/M) and probes on the X chromosome are subsequently shown. **(Distribution plots of methylation percentage.)** Violin plots: grey areas indicate a kernel density plot of the methylation percentage (β) of all probes in all samples in a certain category. The boxplot indicates the interquartile range (black bars) and median (white squares). X-axis labels indicate histological subgroup according to Fig 1A and 1B. TE indicates type I TE only. (**Principal Component Analysis.)** The first two principal components (PC) are plotted to evaluate the discriminative power of the methylation pattern between the subtypes. Abbreviations of histological subtypes are explained in Fig 1A. CL indicates cell lines. Please note that in the legend of the PCA the TE group is subdivided based on gender and localization: I = type I; II = type II/ formally part of the mNS group, s = sacrum, t = testis, o = ovary, m = male, f = female.

doi:10.1371/journal.pone.0122146.g006

adjustment, quantile normalization and BMIQ-based correction for probe type bias (Infinium I vs II) were performed [154,155,157]. Data processing resulted in two quantifications of a CpG site's methylation status: the methylation percentage β and an associated M-value which ($logit_2(\beta)$). M-values were used for statistical computations because of a more favorable

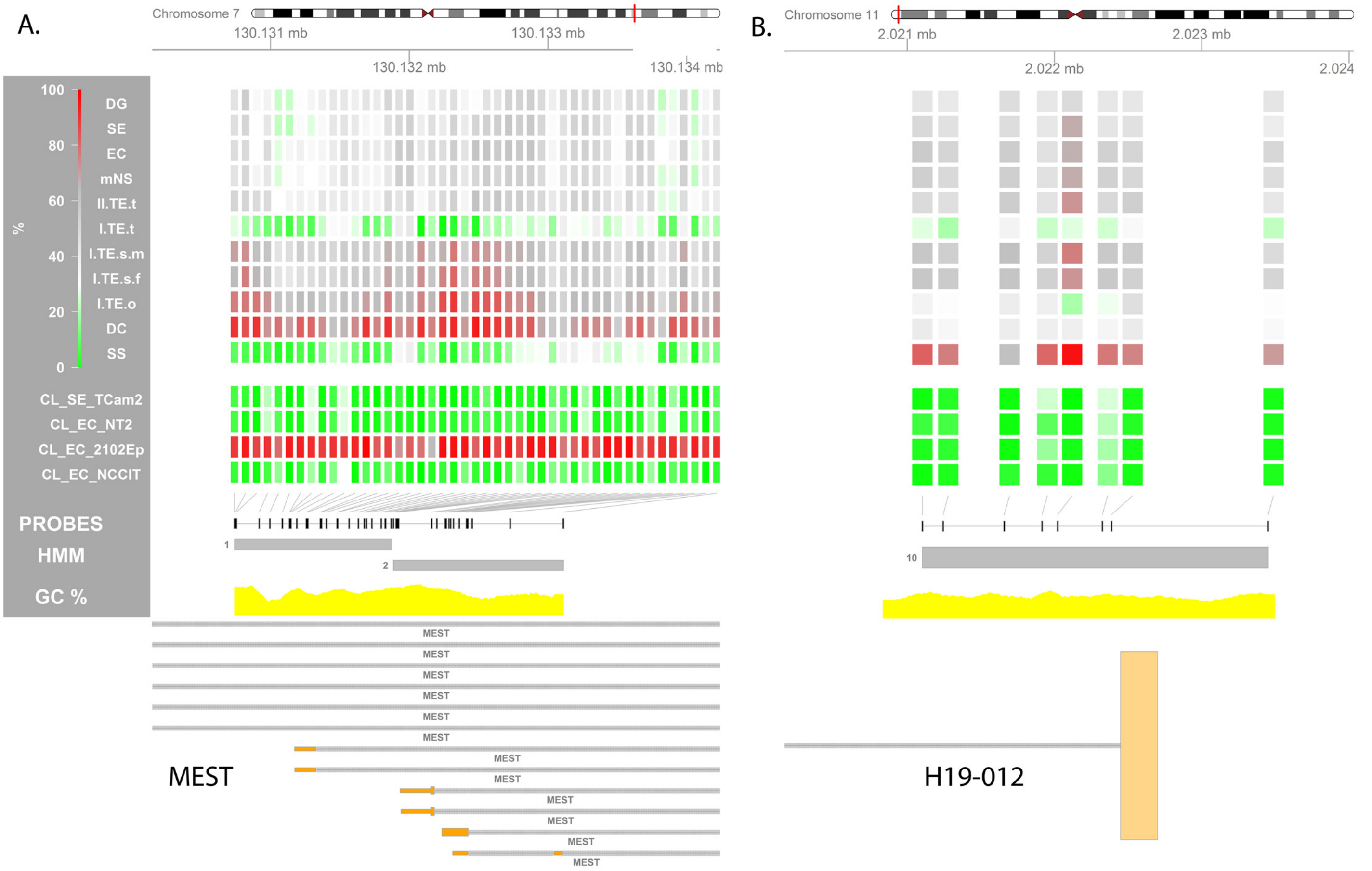

Fig 7. Methylation status of imprinting control regions. Visualization of the methylation percentage at specific loci is used to zoom in on a predefined region and investigate local imprinting differences between GCT subtypes. Two illustrative regions are depicted. **(A) ICR_P: *MEST*. (B) ICR_M: *H19-IGF2*.** The overlapping *H19* transcript is an aberrant, long alternative transcript (H19-012, ENST00000428066). This ICR regulates *H19* and *IGF2* expression and lies upstream all other transcripts of *H19*. The other ICRs are visualized in S6 Fig and listed in S4 Table. **(Visualizations)** From top to bottom the following is depicted: (1) Four-color heat map indicating methylation % for each individual probe in the depicted region. For the sample groups specified on the left the median methylation % is shown. (2) Position of all probes in the region of interest (ROI) is annotated as black rectangles. (3) HMM segments are displayed as grey boxes spanning the segment's width and grouped per state. Numbers indicate the state of each (group of) segment(s). (5) GC% was obtained from the UCSC genome browser database (gc5Base table). (6) Transcripts overlapping with the ROI are plotted at the bottom. Plot generated using the Gviz package. Abbreviations of histological subtypes are explained in Fig 1A. Please note that the TE group is subdivided based on gender and localization: I = type I; II = type II/formally part of the mNS group, s = sacrum, t = testis, o = ovary, m = male, f = female. CL indicates cell lines.

doi:10.1371/journal.pone.0122146.g007

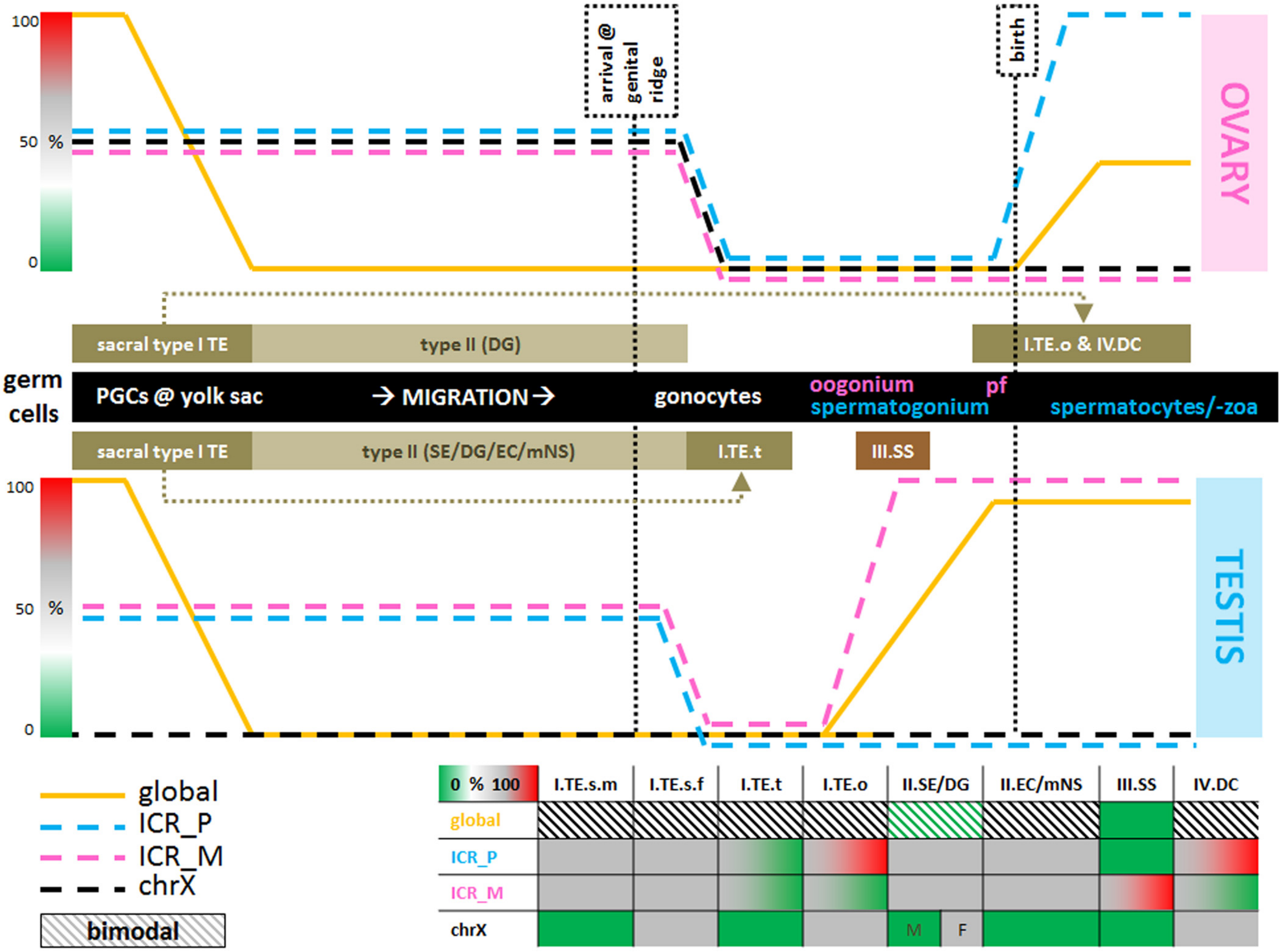

Fig 8. GCT methylation status in context of methylation during germ cell development. The top and bottom line charts depict normal germ cell development in female and male respectively (stages specified in the middle black bar). Methylation status during normal germ cell development is depicted for the global genome, ICRs and chromosome X (see Discussion). Putative cells of origin of the various types of GCTs are indicated in the brown boxes. ICR_P/M = ICR regulating paternally/maternally expressed genes. Bimodal indicates a methylation pattern peaking 0 and 100% with the exception of SE/DG (between 0 and ≈50). The table (bottom) provides a summary of the results, mainly Figs 2 and 6. Abbreviations: pf = primordial follicle. Type I tumors are indicated with their type (I), sex (m = male, f = female) and location (s = sacral, t = testis, o = ovary). Other GCT subtypes are indicated with their type (I, II, IV) and the abbreviation of each histological class, which are explained in the main text. Gradient bars indicate percentages of methylation (0→100%, green-white-grey-red) analogous to the gradient used in the other figures.

doi:10.1371/journal.pone.0122146.g008

tradeoff between true positive rate and detection rate [41]. All data is available via GEO (GSE58538).

(Additional) annotation 450K array.. The 450K annotation manifest (v1.2) as supplied by Illumina contains a number of functional genomic classes like a probe's association with CpG islands, gene coding regions, etc. The manifest was extended with (additional) functional genomic classes, based on the GRch37/hg19 assembly. Briefly, probes close to small nuclear RNAs and microRNAs from snoRNABase and miRBase were identified, as were probes within repeats defined by RepeatMasker (source: UCSC). Probes close to the transcription start site (TSS) of imprinted genes were also identified (geneimprint.com / igc.otago.ac.nz). Known imprinting control regions (ICR) and their association with either paternal or maternal expression were retrieved from WAMIDEX and igc.otago.ac.nz. Imprinting is indicated using the

expressed allele. Illumina probe classes were extended with a number of merged categories. Where applicable, the upstream (-) and downstream (+) margins reported in this manuscript are analogous to the Illumina annotation (-1500+0; -200+0). The eighteen functional categories of primary interest to this manuscript are illustrated in Fig 1C. The extended annotation including its documentation is available at GEO (GPL18809).

Analysis protocol. Below, the subsequent steps of the data analysis are described. More details are presented in S1 Fig. Depending on the context, "feature" can refer to a probe or a segment. All results are based on the GRch37/hg19 assembly.

***Global methylation*:** Violin plots were created per histological subtype using all (global) or functional subsets of 450K probes. Violin plots (vioplot package) integrate the benefits of a boxplot and a kernel density plot. Two-dimensional principal component analysis (PCA) was applied and validated using bootstrapping to assess how well the methylation values of (subsets of) the probes separated the histological subtypes. Formal statistical testing of the distribution of the methylation values identified (small) significant differences between almost all tumor classes (data not shown, Kruskal Wallis test followed by pairwise, Benjamini-Hochberg corrected Mann Whitney U tests). The PCA and violin plot based approach was preferred/used to identify the largest, most relevant differences.

***Defining genomic segments and discriminative methylation states*:** To detect regions of interest rather than only selecting individual differentiating probes (CpG sites) a HMM was trained on the tumor samples. Without a priori information about tumor type, the Hidden Markov Model (HMM) combines adjacent probes into segments and assigns these segments to k mutually exclusive states, each with distinct methylation profiles over all tumor samples. k = 20 was used as the likelihood of the model saturated around this number of states (S1 Fig, page 11). In total, 133,730 segments were identified. The median methylation value (M or β) of all probes in a segment or state was taken as methylation proxy. As a proof of concept, S1 Fig (page 17) shows clear separation of male and female samples based on state 15 which almost exclusively contains probes on the X chromosome. The result of the HMM is included in the GEO submission of the data (GSE58538) and its properties/procedures are summarized in S1 Fig.

***Differentiating features (probes or segments)*:** Features showing low variability over all samples were excluded before formally testing for differential methylation ($\sigma_{M,probes}<0.8$, n = 77,154/437,881 (17,62%) & $\sigma_{M,segments}<0.6$, n = 13,229/133,730 (9,89%), S1 Fig, page 8). A Mann Whitney U test was applied to each feature, comparing the distribution of M values between two histological subtypes. If significant ($p<0.05$, Benjamini-Hochberg corrected [158]), the subtype specificity was validated in 100 stratified bootstrap samples. If the feature proved to be significant in ≥95% of the validation samples and showed a difference in median M values > |0.9| between the pair of histological subtypes it was considered potentially discriminating. The value of 0.9 was chosen as the mean of the cut-off range recommended by Du and coworkers (0.4–1.4) [41]. Although a less stringent setting might result in a higher detection rate, it will considerably reduce the true positive rate [41]. The sign of the difference in median M value was used to assign a relative methylation status (hyper/hypo) in either of the two subtypes under pairwise consideration.

***Differentiating Hidden Markov Model (HMM) states*:** To identify non-adjacent regions that showed similar patterns of methylation a logistic LASSO regression model was fitted on the M values of the HMM states (glmnet packgage) [159]. Coefficients > 0 were selected from the most regularized regression model within 1 standard deviation of the model with minimal cross validation error. A 10 fold cross validated λ was used. Features included in the selected state(s) and showing a difference in median M values > |0.9| (see above) between the pair of histological subtypes compared were considered potentially discriminating. The sign of the difference in median M value was used to assign a relative methylation status (hyper-/hypomethylated) in each of the subtypes.

***Final selection of differentially methylated probes (DMPs)*:** Features of interest were identified in the intersection of (1) all probes in discriminating states, (2) all probes in discriminating segments and (3) all individually discriminating probes (S1 Fig (p. 3/5), also see above). This was done separately for probes that showed relative hypermethylation in either of the subtypes under pairwise comparison. This way, two groups of differentially methylated probes (DMPs) were identified, showing relative hypermethylation in one subtype and relative hypomethylation in the other.

***Functional enrichment*:** The sets of DMPs were subjected to enrichment analysis for 18 functional categories (Fig 1C) using a two sided Fisher's Exact test. Analogously, association with chromosome and state was tested. $p<0.05/(18+24+20) = 0.00080645161$ was considered significant, hence retaining a Bonferroni corrected Type I error rate of 5% (18 functional categories, 24 chromosomes, 20 states).

***Differentially methylated regions (DMRs)*:** Regions with ≥ 5 adjacent DMPs and a maximal inter-DMP distance ≤ 1 kb were identified as DMRs between the tumor groups. Annotations were retrieved for DMRs including flanking regions of 20% of the length of each DMR.

***Analysis of the cell lines*:** Cell lines were compared to tumor samples in the evaluation of specific regions of interest in the tumor samples and with regard to their global methylation profile. Moreover, they were analyzed using the DMRforPairs package to identify specific DMR in these unique samples ([160], using the default settings except min_dM = 0.9, see above). For the NCCIT and TCam-2 cell lines this analysis matches the one performed in [69].

Software. Analyses were performed in R 3.1.0/Bioconductor 2.14 (Windows 7 x64) and 2.15.2/Bioconductor 2.11 (Redhat Linux x64). Additional R packages: parallel & gplots. The HMM was trained using Matlab R2012a (64 bits) using the MOGHMM toolbox.

Supporting Information

S1 Fig. Supplementary methods. A detailed flowchart and description of the analysis protocol is presented, followed by a visual illustration of the selection of differentially methylated features. The motivation for the threshold when filtering low variability probes is presented next. Finally, the properties of the Hidden Markov Model (HMM) are presented together with a detailed description of is construction. HMM state 15 is presented as a proof of concept, discriminating between male and female samples based on X-chomosomal localization of the large majority of the probes in this class.
(PDF)

S2 Fig. A, Methylation patterns in GCT subtypes and cell lines—All categories and validation. In addition to the selected categories presented in Fig 2 this figure contains all 18 functional categories presented in Fig 1C and includes the primary PCA as well as an its validation (i.e. robustness of the result). PCA was performed on the total dataset (left) and validated using stratified bootstrapping (middle: training, right: validation). (Distribution plot of methylation percentage) Violin plots: grey areas indicate a kernel density plot of the methylation percentage (β) of all probes in all samples in a certain category. The boxplot indicates the interquartile range (black bars) and median (white squares). X-axis labels indicate histological subgroup according to Fig 1A and 1B. TE indicates type I TE only. (Principal Component Analysis) The first two principal components (PC) are plotted to evaluate the discriminative power of the methylation pattern between the subtypes. Abbreviations of histological subtypes are explained in Fig 1A. CL indicates cell lines. Please note that in the legend of the PCA the TE group is subdivided based on gender and localization: I = type I; II = type II/formally part of the mNS group, s = sacrum, t = testis, o = ovary, m = male, f = female. S2B Fig, Methylation patterns in GCT subtypes and cell lines—Global methylation patterns in individual samples. X-axis indicates arbitrary sample ID. The sex of the patient from which the sample originates is indicated in blue (male) or red

(female). Density plots are explained in the legend of Fig 2. Distributions are shown for all probes individual per sample. The ICR_P and ICR_M categories are presented separately to facilitate the discussion about imprinting. The red dashed line indicates somatic imprinting (50%). Please note that details on the TE group are presented in the main text (Fig 6D) and that this category is therefore omitted here. This also holds for the n = 3 type II pure TE included in the mNS group. (Distribution plot of methylation percentage) Violin plots: grey areas indicate a kernel density plot of the methylation percentage (β) of all probes in all samples in a certain category. The boxplot indicates the interquartile range (black bars) and median (white squares). X-axis labels indicate histological subgroup according to Fig 1A and 1B. TE indicates type I TE only.
(PDF)

S3 Fig. A, Enrichment of differentially methylated probes (DMPs) for chromosomal position and HMM state—Merged GCT subtypes in pairwise comparisons. The SE+DG and EC+mNS categories were merged because of high similarity in biological classification and methylation profile. Despite their similarities, the DC and type I TE because they belong to different histological classes. S3B Fig, Enrichment of differentially methylated probes (DMPs) for chromosomal position and HMM state—Association between DMPs and chromosome / HMM state. Stacked bar charts indicate the fraction of probes in a subset (DMP[A-B], DMP [A-B], non-DMP) that is mapped to a specific chromosome or assigned to a specific state. Grey indicates the non-DMPs and red and green indicated the DMPs hypermethylated in the subtype with the matching color in the figure (alternating green/white = A, alternating red/white = B). * = significant over-/underrepresentation of DMPs relative to the non-DMP subset (tested per chromosome/state, 2-sided Fisher's exact test, see Methods for Bonferroni corrected α threshold). In the right bottom of each figure the coefficients of the LASSO regression model are depicted. These roughly match the strongest over- and underrepresentations identified by the Fisher's Exact tests on the states. The LASSO selected states are marked orange in the table indicating the significant associations between each state and either DMP group.
(PDF)

S4 Fig. A, Methylation profile at GCT subtype specific differentially methylated regions (DMRs)—continued—SE/DG versus type I TE. This figure depicts the DMRs between GCT subtypes discussed in the main text in addition to those already visualized in Fig 4. (Visualizations) From top to bottom the following is depicted: (1) Four-color heat map indicating methylation % for each individual probe in the depicted region. For the sample groups specified on the left the median methylation % is shown. (2) Position of all probes in the region of interest (ROI) is annotated as black rectangles. (3) HMM segments are displayed as grey boxes spanning the segment's width and grouped per state. Numbers indicate the state of each (group of) segment (s). (5) GC% was obtained from the UCSC genome browser database (gc5Base table). (6) Transcripts overlapping with the ROI are plotted at the bottom. Plot generated using the Gviz package. Abbreviations of histological subtypes are explained in Fig 1A. Please note that the TE group is subdivided based on gender and localization: I = type I; II = type II/formally part of the mNS group, s = sacrum, t = testis, o = ovary, m = male, f = female. CL indicates cell lines. S4B Fig, Methylation profile at GCT subtype specific differentially methylated regions (DMRs)—continued—EC/mNS versus type I TE. This figure depicts the DMRs between GCT subtypes discussed in the main text in addition to those already visualized in Fig 4. (Visualizations) From top to bottom the following is depicted: (1) Four-color heat map indicating methylation % for each individual probe in the depicted region. For the sample groups specified on the left the median methylation % is shown. (2) Position of all probes in the region of interest (ROI) is annotated as black rectangles. (3) HMM segments are displayed as grey boxes spanning the segment's width and grouped per state. Numbers indicate the state of each (group of) segment(s).

(5) GC% was obtained from the UCSC genome browser database (gc5Base table). (6) Transcripts overlapping with the ROI are plotted at the bottom. Plot generated using the Gviz package. Abbreviations of histological subtypes are explained in Fig 1A. Please note that the TE group is subdivided based on gender and localization: I = type I; II = type II/formally part of the mNS group, s = sacrum, t = testis, o = ovary, m = male, f = female. CL indicates cell lines. S4C Fig. Methylation profile at GCT subtype specific differentially methylated regions (DMRs)—continued—SE/DG versus SS. This figure depicts the DMRs between GCT subtypes discussed in the main text in addition to those already visualized in Fig 4. (Visualizations) From top to bottom the following is depicted: (1) Four-color heat map indicating methylation % for each individual probe in the depicted region. For the sample groups specified on the left the median methylation % is shown. (2) Position of all probes in the region of interest (ROI) is annotated as black rectangles. (3) HMM segments are displayed as grey boxes spanning the segment's width and grouped per state. Numbers indicate the state of each (group of) segment(s). (5) GC% was obtained from the UCSC genome browser database (gc5Base table). (6) Transcripts overlapping with the ROI are plotted at the bottom. Plot generated using the Gviz package. Abbreviations of histological subtypes are explained in Fig 1A. Please note that the TE group is subdivided based on gender and localization: I = type I; II = type II/formally part of the mNS group, s = sacrum, t = testis, o = ovary, m = male, f = female. CL indicates cell lines.
(PDF)

S5 Fig. A, Methylation status of GCT specific genes. This figure (together with S5B Fig) depicts the genes discussed in the main text and Table 2 in addition to those already visualized in Fig 5. Genes are annotated 1.5kb upstream of their TSS and 1.5kb downstream of their transcription termination site. (Visualizations) From top to bottom the following is depicted: (1) Four-color heat map indicating methylation % for each individual probe in the depicted region. For the sample groups specified on the left the median methylation % is shown. (2) Position of all probes in the region of interest (ROI) is annotated as black rectangles. (3) HMM segments are displayed as grey boxes spanning the segment's width and grouped per state. Numbers indicate the state of each (group of) segment(s). (5) GC% was obtained from the UCSC genome browser database (gc5Base table). (6) Transcripts overlapping with the ROI are plotted at the bottom. Plot generated using the Gviz package. Abbreviations of histological subtypes are explained in Fig 1A. Please note that the TE group is subdivided based on gender and localization: I = type I; II = type II/formally part of the mNS group, s = sacrum, t = testis, o = ovary, m = male, f = female. CL indicates cell lines. S5B Fig. Methylation status of genes with SNPs significantly associated with GCTs. This figure (together with S5A Fig) depicts the genes discussed in the main text and Table 2 in addition to those already visualized in Fig 5. Genes are annotated 1.5kb upstream of their TSS and 1.5kb downstream of their transcription termination site. (Visualizations) From top to bottom the following is depicted: (1) Four-color heat map indicating methylation % for each individual probe in the depicted region. For the sample groups specified on the left the median methylation % is shown. (2) Position of all probes in the region of interest (ROI) is annotated as black rectangles. (3) HMM segments are displayed as grey boxes spanning the segment's width and grouped per state. Numbers indicate the state of each (group of) segment(s). (5) GC% was obtained from the UCSC genome browser database (gc5Base table). (6) Transcripts overlapping with the ROI are plotted at the bottom. Plot generated using the Gviz package. Abbreviations of histological subtypes are explained in Fig 1A. Please note that the TE group is subdivided based on gender and localization: I = type I; II = type II/formally part of the mNS group, s = sacrum, t = testis, o = ovary, m = male, f = female. CL indicates cell lines.
(PDF)

S6 Fig. Methylation status of known imprinting control regions (ICRs). ICRs identified as described in the materials and methods sections were checked for coverage on the 450K array. 21/28 unique ICRs were covered by one or more probes. These were visualized here (overview: S4 Table). *H19_IGF2* regions: the overlapping transcript is an aberrant, long alternative transcript (*H19-012*, ENST00000428066). These ICRs regulates *H19* and *IGF2* expression and lie upstream all other transcripts of H19. (Visualizations) From top to bottom the following is depicted: (1) Four-color heat map indicating methylation % for each individual probe in the depicted region. For the sample groups specified on the left the median methylation % is shown. (2) Position of all probes in the region of interest (ROI) is annotated as black rectangles. (3) HMM segments are displayed as grey boxes spanning the segment's width and grouped per state. Numbers indicate the state of each (group of) segment(s). (5) GC% was obtained from the UCSC genome browser database (gc5Base table). (6) Transcripts overlapping with the ROI are plotted at the bottom. Plot generated using the Gviz package. Abbreviations of histological subtypes are explained in Fig 1A. Please note that the TE group is subdivided based on gender and localization: I = type I; II = type II/formally part of the mNS group, s = sacrum, t = testis, o = ovary, m = male, f = female. CL indicates cell lines.
(PDF)

S1 Table. List of DMPs resulting from pairwise comparison of GCT subtypes.
(XLSX)

S2 Table. Counts, percentages, log scores and statistical test results for enrichment in functional genomic categories. (A) SE/DG vs EC/mNS; (B) SE/DG vs type I TE; (C) EC/mNS vs type I TE; (D) SE/DG vs SS. Rows indicate the functional categories. Columns indicate the number of probes in the non-DMP and both subtype specific DMP sets. Next, the fraction (%) of this count relative to all non-DMPs or either set of DMPs is presented. The log-scores are calculated as $\log_2$(%DMP/%non-DMPs) and visually presented in Fig 3 for those categories showing significant over-/underrepresentation. Significance of the enrichment was evaluated using a two-sided Fisher Exact test with a Bonferroni corrected α threshold as specified in the Materials & Methods section.
(XLSX)

S3 Table. (DMRs between tumor groups). List of DMRs for each pair of GCT subtypes. (Recurrent tumor DMRs) Gene symbols that occurred in more than one DMR; either irrespective of DMR subset (n.total.occurences) or in multiple independent DMR subsets (n.dmr.lists) (Overlap tumor and CL DMRs) Gene symbols involved in DMRs identified between both the tumor groups and the cell lines. The second column indicates in which tumor comparisons the gene symbol was involved in a DMR.
(XLSX)

S4 Table. Merged known ICRs from literature with sources. Also see S6 Fig for a visual representation of the methylation status at the ICRs if covered by the 450K array (21/28).
(CSV)

Acknowledgments

The authors thank the Department of Bioinformatics, Erasmus MC, Rotterdam, for their support. They especially thank Ms Sylvia de Does and Mr Ivo Palli. Prof. dr. J.W. Oosterhuis, Department of Pathology, Erasmus MC, Rotterdam is kindly acknowledged for his critical reading of the manuscript.

Author Contributions

Conceived and designed the experiments: MR AG LD LL. Performed the experiments: AG. Analyzed the data: MR LD DT JR. Contributed reagents/materials/analysis tools: DT LD DK JR. Wrote the paper: MR DT AG LD DK JR LL.

References

1. Messerschmidt DM (2012) Should I stay or should I go: protection and maintenance of DNA methylation at imprinted genes. Epigenetics 7: 969–975. PMID: 22869105
2. Popp C, Dean W, Feng S, Cokus SJ, Andrews S, Pellegrini M, et al. (2010) Genome-wide erasure of DNA methylation in mouse primordial germ cells is affected by AID deficiency. Nature 463: 1101-U1126. doi: 10.1038/nature08829 PMID: 20098412
3. Reik W, Dean W, Walter J (2001) Epigenetic reprogramming in mammalian development. Science 293: 1089–1093. PMID: 11498579
4. Seisenberger S, Andrews S, Krueger F, Arand J, Walter J, Santos F, et al. (2012) The dynamics of genome-wide DNA methylation reprogramming in mouse primordial germ cells. Mol Cell 48: 849–862. doi: 10.1016/j.molcel.2012.11.001 PMID: 23219530
5. Seisenberger S, Peat JR, Reik W (2013) Conceptual links between DNA methylation reprogramming in the early embryo and primordial germ cells. Curr Opin Cell Biol 25: 281–288. doi: 10.1016/j.ceb.2013.02.013 PMID: 23510682
6. Wang L, Zhang J, Duan J, Gao X, Zhu W, Lu X, et al. (2014) Programming and inheritance of parental DNA methylomes in mammals. Cell 157: 979–991. doi: 10.1016/j.cell.2014.04.017 PMID: 24813617
7. Matsui Y, Mochizuki K (2014) A current view of the epigenome in mouse primordial germ cells. Mol Reprod Dev 81: 160–170. doi: 10.1002/mrd.22214 PMID: 23868517
8. Saitou M, Kagiwada S, Kurimoto K (2012) Epigenetic reprogramming in mouse pre-implantation development and primordial germ cells. Development 139: 15–31. doi: 10.1242/dev.050849 PMID: 22147951
9. Payer B, Lee JT, Namekawa SH (2011) X-inactivation and X-reactivation: epigenetic hallmarks of mammalian reproduction and pluripotent stem cells. Hum Genet 130: 265–280. doi: 10.1007/s00439-011-1024-7 PMID: 21667284
10. Ohhata T, Wutz A (2013) Reactivation of the inactive X chromosome in development and reprogramming. Cell Mol Life Sci 70: 2443–2461. doi: 10.1007/s00018-012-1174-3 PMID: 23052214
11. Deuve JL, Avner P (2011) The coupling of X-chromosome inactivation to pluripotency. Annu Rev Cell Dev Biol 27: 611–629. doi: 10.1146/annurev-cellbio-092910-154020 PMID: 21801017
12. de Napoles M, Nesterova T, Brockdorff N (2007) Early loss of Xist RNA expression and inactive X chromosome associated chromatin modification in developing primordial germ cells. PLoS One 2: e860. PMID: 17848991
13. Kristensen DG, Skakkebaek NE, Rajpert-De Meyts E, Almstrup K (2013) Epigenetic features of testicular germ cell tumours in relation to epigenetic characteristics of foetal germ cells. Int J Dev Biol 57: 309–317. doi: 10.1387/ijdb.130142ka PMID: 23784842
14. Wermann H, Stoop H, Gillis AJ, Honecker F, van Gurp RJ, Ammerpohl O, et al. (2010) Global DNA methylation in fetal human germ cells and germ cell tumours: association with differentiation and cisplatin resistance. J Pathol 221: 433–442. doi: 10.1002/path.2725 PMID: 20593487
15. Almstrup K, Nielsen JE, Mlynarska O, Jansen MT, Jorgensen A, Skakkebaek NE, et al. (2010) Carcinoma in situ testis displays permissive chromatin modifications similar to immature foetal germ cells. Br J Cancer 103: 1269–1276. doi: 10.1038/sj.bjc.6605880 PMID: 20823885
16. Oosterhuis JW, Looijenga LH (2005) Testicular germ-cell tumours in a broader perspective. Nat Rev Cancer 5: 210–222. PMID: 15738984
17. Looijenga LH (2009) Human testicular (non)seminomatous germ cell tumours: the clinical implications of recent pathobiological insights. J Pathol 218: 146–162. doi: 10.1002/path.2522 PMID: 19253916
18. Woodward PJ, Heidenreich A, Looijenga LHJ, Oosterhuis JW, McLeod DG, Moller H, et al. (2004) Testicular germ cell tumors. In: Eble JN, Sauter G, Epstein JI, Sesterhann IA, eds, editors. World Health Organization Classification of Tumours Pathology and Genetics of the Urinary System and Male Genital Organs. Lyon: IARC Press. pp. 17–278.
19. Rijlaarsdam MA, Looijenga LH (2014) An oncofetal and developmental perspective on testicular germ cell cancer. Semin Cancer Biol.

20. Amatruda JF, Ross JA, Christensen B, Fustino NJ, Chen KS, Hooten AJ, et al. (2013) DNA methylation analysis reveals distinct methylation signatures in pediatric germ cell tumors. BMC Cancer 13: 313. doi: 10.1186/1471-2407-13-313 PMID: 23806198

21. Van Der Zwan YG, Stoop H, Rossello F, White SJ, Looijenga LH (2013) Role of epigenetics in the etiology of germ cell cancer. Int J Dev Biol 57: 299–308. doi: 10.1387/ijdb.130017ll PMID: 23784841

22. Boublikova L, Buchler T, Stary J, Abrahamova J, Trka J (2014) Molecular biology of testicular germ cell tumors: unique features awaiting clinical application. Crit Rev Oncol Hematol 89: 366–385. doi: 10.1016/j.critrevonc.2013.10.001 PMID: 24182421

23. Gobel U, Schneider DT, Calaminus G, Haas RJ, Schmidt P, Harms D (2000) Germ-cell tumors in childhood and adolescence. GPOH MAKEI and the MAHO study groups. Ann Oncol 11: 263–271. PMID: 10811491

24. Rescorla FJ (1999) Pediatric germ cell tumors. Semin Surg Oncol 16: 144–158. PMID: 9988869

25. Sievers S, Alemazkour K, Zahn S, Perlman EJ, Gillis AJ, Looijenga LH, et al. (2005) IGF2/H19 imprinting analysis of human germ cell tumors (GCTs) using the methylation-sensitive single-nucleotide primer extension method reflects the origin of GCTs in different stages of primordial germ cell development. Genes Chromosomes Cancer 44: 256–264. PMID: 16001432

26. Horwich A, Shipley J, Huddart R (2006) Testicular germ-cell cancer. Lancet 367: 754–765. PMID: 16517276

27. Looijenga LH, Van Agthoven T, Biermann K (2013) Development of malignant germ cells—the genvironmental hypothesis. Int J Dev Biol 57: 241–253. doi: 10.1387/ijdb.130026ll PMID: 23784835

28. Kratz CP, Mai PL, Greene MH (2010) Familial testicular germ cell tumours. Best Pract Res Clin Endocrinol Metab 24: 503–513. doi: 10.1016/j.beem.2010.01.005 PMID: 20833340

29. Chung CC, Kanetsky PA, Wang Z, Hildebrandt MA, Koster R, Skotheim RI, et al. (2013) Meta-analysis identifies four new loci associated with testicular germ cell tumor. Nat Genet.

30. Dieckmann KP, Pichlmeier U (2004) Clinical epidemiology of testicular germ cell tumors. World J Urol 22: 2–14. PMID: 15034740

31. Cools M, Drop SL, Wolffenbuttel KP, Oosterhuis JW, Looijenga LH (2006) Germ cell tumors in the intersex gonad: old paths, new directions, moving frontiers. Endocr Rev 27: 468–484. PMID: 16735607

32. Czene K, Lichtenstein P, Hemminki K (2002) Environmental and heritable causes of cancer among 9.6 million individuals in the Swedish Family-Cancer Database. Int J Cancer 99: 260–266. PMID: 11979442

33. Oosterhuis JW, Stoop H, Honecker F, Looijenga LH (2007) Why human extragonadal germ cell tumours occur in the midline of the body: old concepts, new perspectives. Int J Androl 30: 256–263; discussion 263–254. PMID: 17705807

34. Fan R, Ulbright TM (2012) Does intratubular germ cell neoplasia, unclassified type exist in prepubertal, cryptorchid testes? Fetal Pediatr Pathol 31: 21–24. doi: 10.3109/15513815.2011.618874 PMID: 22026641

35. Leman ES, Gonzalgo ML (2010) Prognostic features and markers for testicular cancer management. Indian J Urol 26: 76–81. doi: 10.4103/0970-1591.60450 PMID: 20535291

36. Looijenga LH (2011) Spermatocytic seminoma: toward further understanding of pathogenesis. J Pathol 224: 431–433. doi: 10.1002/path.2939 PMID: 21725972

37. Lim J, Goriely A, Turner GD, Ewen KA, Jacobsen GK, Graem N, et al. (2011) OCT2, SSX and SAGE1 reveal the phenotypic heterogeneity of spermatocytic seminoma reflecting distinct subpopulations of spermatogonia. J Pathol 224: 473–483. doi: 10.1002/path.2919 PMID: 21706474

38. Verdorfer I, Rogatsch H, Tzankov A, Steiner H, Mikuz G (2004) Molecular cytogenetic analysis of human spermatocytic seminomas. J Pathol 204: 277–281. PMID: 15476269

39. Rajpert-De Meyts E, Jacobsen GK, Bartkova J, Aubry F, Samson M, Bartek J, et al. (2003) The immunohistochemical expression pattern of Chk2, p53, p19INK4d, MAGE-A4 and other selected antigens provides new evidence for the premeiotic origin of spermatocytic seminoma. Histopathology 42: 217–226. PMID: 12605640

40. Chen YT, Chiu R, Lee P, Beneck D, Jin B, Old LJ (2011) Chromosome X-encoded cancer/testis antigens show distinctive expression patterns in developing gonads and in testicular seminoma. Hum Reprod 26: 3232–3243. doi: 10.1093/humrep/der330 PMID: 22016418

41. Du P, Zhang X, Huang CC, Jafari N, Kibbe WA, Hou L, et al. (2010) Comparison of Beta-value and M-value methods for quantifying methylation levels by microarray analysis. BMC Bioinformatics 11: 587. doi: 10.1186/1471-2105-11-587 PMID: 21118553

42. Nettersheim D, Heukamp LC, Fronhoffs F, Grewe MJ, Haas N, Waha A, et al. (2013) Analysis of TET Expression/Activity and 5mC Oxidation during Normal and Malignant Germ Cell Development. PLoS One 8: e82881. doi: 10.1371/journal.pone.0082881 PMID: 24386123

43. Netto GJ, Nakai Y, Nakayama M, Jadallah S, Toubaji A, Nonomura N, et al. (2008) Global DNA hypomethylation in intratubular germ cell neoplasia and seminoma, but not in nonseminomatous male germ cell tumors. Mod Pathol 21: 1337–1344. doi: 10.1038/modpathol.2008.127 PMID: 18622385

44. Jeyapalan JN, Noor DA, Lee SH, Tan CL, Appleby VA, Kilday JP, et al. (2011) Methylator phenotype of malignant germ cell tumours in children identifies strong candidates for chemotherapy resistance. Br J Cancer 105: 575–585. doi: 10.1038/bjc.2011.218 PMID: 21712824

45. Ushida H, Kawakami T, Minami K, Chano T, Okabe H, Okada Y, et al. (2012) Methylation profile of DNA repetitive elements in human testicular germ cell tumor. Mol Carcinog 51: 711–722. doi: 10.1002/mc.20831 PMID: 21809391

46. Looijenga LH, Hersmus R, Gillis AJ, Pfundt R, Stoop HJ, van Gurp RJ, et al. (2006) Genomic and expression profiling of human spermatocytic seminomas: primary spermatocyte as tumorigenic precursor and DMRT1 as candidate chromosome 9 gene. Cancer Res 66: 290–302. PMID: 16397242

47. Wu J, Bao J, Wang L, Hu Y, Xu C (2011) MicroRNA-184 downregulates nuclear receptor corepressor 2 in mouse spermatogenesis. BMC Dev Biol 11: 64. doi: 10.1186/1471-213X-11-64 PMID: 22017809

48. Bremmer F, Thelen P, Pottek T, Behnes CL, Radzun HJ, Schweyer S (2012) Expression and function of the vitamin D receptor in malignant germ cell tumour of the testis. Anticancer Res 32: 341–349. PMID: 22213325

49. Funk CD, Funk LB, FitzGerald GA, Samuelsson B (1992) Characterization of human 12-lipoxygenase genes. Proc Natl Acad Sci U S A 89: 3962–3966. PMID: 1570320

50. Salpea P, Russanova VR, Hirai TH, Sourlingas TG, Sekeri-Pataryas KE, Romero R, et al. (2012) Postnatal development- and age-related changes in DNA-methylation patterns in the human genome. Nucleic Acids Res 40: 6477–6494. doi: 10.1093/nar/gks312 PMID: 22495928

51. Matson CK, Murphy MW, Sarver AL, Griswold MD, Bardwell VJ, Zarkower D (2011) DMRT1 prevents female reprogramming in the postnatal mammalian testis. Nature 476: 101–104. doi: 10.1038/nature10239 PMID: 21775990

52. Sarkardeh H, Totonchi M, Asadpour O, Sadighi Gilani MA, Zamani Esteki M, Almadani N, et al. (2014) Association of MOV10L1 gene polymorphisms and male infertility in azoospermic men with complete maturation arrest. J Assist Reprod Genet.

53. Frost RJ, Hamra FK, Richardson JA, Qi X, Bassel-Duby R, Olson EN (2010) MOV10L1 is necessary for protection of spermatocytes against retrotransposons by Piwi-interacting RNAs. Proc Natl Acad Sci U S A 107: 11847–11852. doi: 10.1073/pnas.1007158107 PMID: 20547853

54. Kano K, Kitamura A, Matsuwaki T, Morimatsu M, Naito K (2010) Discoidin domain receptor 2 (DDR2) is required for maintenance of spermatogenesis in male mice. Mol Reprod Dev 77: 29–37. doi: 10.1002/mrd.21093 PMID: 19681157

55. Bonnard C, Strobl AC, Shboul M, Lee H, Merriman B, Nelson SF, et al. (2012) Mutations in IRX5 impair craniofacial development and germ cell migration via SDF1. Nat Genet 44: 709–713. doi: 10.1038/ng.2259 PMID: 22581230

56. Le Bouffant R, Souquet B, Duval N, Duquenne C, Herve R, Frydman N, et al. (2011) Msx1 and Msx2 promote meiosis initiation. Development 138: 5393–5402. doi: 10.1242/dev.068452 PMID: 22071108

57. Lind GE, Skotheim RI, Fraga MF, Abeler VM, Esteller M, Lothe RA (2006) Novel epigenetically deregulated genes in testicular cancer include homeobox genes and SCGB3A1 (HIN-1). J Pathol 210: 441–449. PMID: 17029216

58. Griffeth RJ, Carretero J, Burks DJ (2013) Insulin receptor substrate 2 is required for testicular development. PLoS One 8: e62103. doi: 10.1371/journal.pone.0062103 PMID: 23741292

59. de Haas EC, Zwart N, Meijer C, Suurmeijer AJ, Meijer K, Guchelaar HJ, et al. (2010) Association of PAI-1 gene polymorphism with survival and chemotherapy-related vascular toxicity in testicular cancer. Cancer 116: 5628–5636. doi: 10.1002/cncr.25300 PMID: 20737565

60. Ebisch IM, Steegers-Theunissen RP, Sweep FC, Zielhuis GA, Geurts-Moespot A, Thomas CM (2007) Possible role of the plasminogen activation system in human subfertility. Fertil Steril 87: 619–626. PMID: 17123524

61. Graham PL, Kimble J (1993) The mog-1 gene is required for the switch from spermatogenesis to oogenesis in Caenorhabditis elegans. Genetics 133: 919–931. PMID: 8462850

62. Graham PL, Schedl T, Kimble J (1993) More mog genes that influence the switch from spermatogenesis to oogenesis in the hermaphrodite germ line of Caenorhabditis elegans. Dev Genet 14: 471–484. PMID: 8111975

63. Eini R, Dorssers LC, Looijenga LH (2013) Role of stem cell proteins and microRNAs in embryogenesis and germ cell cancer. Int J Dev Biol 57: 319–332. doi: 10.1387/ijdb.130020re PMID: 23784843

64. de Jong J, Stoop H, Gillis AJ, van Gurp RJ, van de Geijn GJ, Boer M, et al. (2008) Differential expression of SOX17 and SOX2 in germ cells and stem cells has biological and clinical implications. J Pathol 215: 21–30. doi: 10.1002/path.2332 PMID: 18348160

65. Rijlaarsdam MA, van Herk HA, Gillis AJ, Stoop H, Jenster G, Martens J, et al. (2011) Specific detection of OCT3/4 isoform A/B/B1 expression in solid (germ cell) tumours and cell lines: confirmation of OCT3/4 specificity for germ cell tumours. Br J Cancer 105: 854–863. doi: 10.1038/bjc.2011.270 PMID: 21847120

66. Kato N, Shibuya H, Fukase M, Tamura G, Motoyama T (2006) Involvement of adenomatous polyposis coli (APC) gene in testicular yolk sac tumor of infants. Hum Pathol 37: 48–53. PMID: 16360415

67. Looijenga LH, Gillis AJ, van Gurp RJ, Verkerk AJ, Oosterhuis JW (1997) X inactivation in human testicular tumors. XIST expression and androgen receptor methylation status. Am J Pathol 151: 581–590. PMID: 9250171

68. Zhang C, Kawakami T, Okada Y, Okamoto K (2005) Distinctive epigenetic phenotype of cancer testis antigen genes among seminomatous and nonseminomatous testicular germ-cell tumors. Genes Chromosomes Cancer 43: 104–112. PMID: 15672408

69. van der Zwan YG, Rijlaarsdam MA, Rossello FJ, Notini AJ, de Boer S, Watkins DN, et al. (2014) Seminoma and embryonal carcinoma footprints identified by analysis of integrated genome-wide epigenetic and expression profiles of germ cell cancer cell lines. PLoS One 9: e98330. doi: 10.1371/journal.pone.0098330 PMID: 24887064

70. Rathi A, Virmani AK, Harada K, Timmons CF, Miyajima K, Hay RJ, et al. (2003) Aberrant methylation of the HIC1 promoter is a frequent event in specific pediatric neoplasms. Clin Cancer Res 9: 3674–3678. PMID: 14506157

71. Koul S, McKiernan JM, Narayan G, Houldsworth J, Bacik J, Dobrzynski DL, et al. (2004) Role of promoter hypermethylation in Cisplatin treatment response of male germ cell tumors. Mol Cancer 3: 16. PMID: 15149548

72. Yabuta Y, Kurimoto K, Ohinata Y, Seki Y, Saitou M (2006) Gene expression dynamics during germline specification in mice identified by quantitative single-cell gene expression profiling. Biol Reprod 75: 705–716. PMID: 16870942

73. Lennartsson J, Ronnstrand L (2012) Stem cell factor receptor/c-Kit: from basic science to clinical implications. Physiol Rev 92: 1619–1649. doi: 10.1152/physrev.00046.2011 PMID: 23073628

74. McLaren A (2003) Primordial germ cells in the mouse. Dev Biol 262: 1–15. PMID: 14512014

75. Mithraprabhu S, Loveland KL (2009) Control of KIT signalling in male germ cells: what can we learn from other systems? Reproduction 138: 743–757. doi: 10.1530/REP-08-0537 PMID: 19567460

76. Gkountela S, Li Z, Vincent JJ, Zhang KX, Chen A, Pellegrini M, et al. (2013) The ontogeny of cKIT+ human primordial germ cells proves to be a resource for human germ line reprogramming, imprint erasure and in vitro differentiation. Nat Cell Biol 15: 113–122. doi: 10.1038/ncb2638 PMID: 23242216

77. Mamsen LS, Brochner CB, Byskov AG, Mollgard K (2012) The migration and loss of human primordial germ stem cells from the hind gut epithelium towards the gonadal ridge. Int J Dev Biol 56: 771–778. doi: 10.1387/ijdb.120202lm PMID: 23417399

78. Farini D, La Sala G, Tedesco M, De Felici M (2007) Chemoattractant action and molecular signaling pathways of Kit ligand on mouse primordial germ cells. Dev Biol 306: 572–583. PMID: 17467686

79. Runyan C, Schaible K, Molyneaux K, Wang Z, Levin L, Wylie C (2006) Steel factor controls midline cell death of primordial germ cells and is essential for their normal proliferation and migration. Development 133: 4861–4869. PMID: 17107997

80. Devouassoux-Shisheboran M, Mauduit C, Tabone E, Droz JP, Benahmed M (2003) Growth regulatory factors and signalling proteins in testicular germ cell tumours. APMIS 111: 212–224; discussion 224. PMID: 12752264

81. Oatley JM, Brinster RL (2012) The germline stem cell niche unit in mammalian testes. Physiol Rev 92: 577–595. doi: 10.1152/physrev.00025.2011 PMID: 22535892

82. Rossi P, Dolci S, Sette C, Geremia R (2003) Molecular mechanisms utilized by alternative c-kit gene products in the control of spermatogonial proliferation and sperm-mediated egg activation. Andrologia 35: 71–78. PMID: 12558531

83. Honecker F, Stoop H, de Krijger RR, Chris Lau YF, Bokemeyer C, Looijenga LH (2004) Pathobiological implications of the expression of markers of testicular carcinoma in situ by fetal germ cells. J Pathol 203: 849–857. PMID: 15221945

84. Rajpert-de Meyts E, Hoei-Hansen CE (2007) From gonocytes to testicular cancer: the role of impaired gonadal development. Ann N Y Acad Sci 1120: 168–180. doi: 10.1196/annals.1411.013 PMID: 18184914

85. Rorth M, Rajpert-De Meyts E, Andersson L, Dieckmann KP, Fossa SD, Grigor KM, et al. (2000) Carcinoma in situ in the testis. Scand J Urol Nephrol Suppl: 166–186. PMID: 11144894

86. Goddard NC, McIntyre A, Summersgill B, Gilbert D, Kitazawa S, Shipley J (2007) KIT and RAS signalling pathways in testicular germ cell tumours: new data and a review of the literature. Int J Androl 30: 337–348; discussion 349. PMID: 17573850

87. Izquierdo MA, Van der Valk P, Van Ark-Otte J, Rubio G, Germa-Lluch JR, Ueda R, et al. (1995) Differential expression of the c-kit proto-oncogene in germ cell tumours. J Pathol 177: 253–258. PMID: 8551387

88. Leroy X, Augusto D, Leteurtre E, Gosselin B (2002) CD30 and CD117 (c-kit) used in combination are useful for distinguishing embryonal carcinoma from seminoma. J Histochem Cytochem 50: 283–285. PMID: 11799147

89. Strohmeyer T, Reese D, Press M, Ackermann R, Hartmann M, Slamon D (1995) Expression of the c-kit proto-oncogene and its ligand stem cell factor (SCF) in normal and malignant human testicular tissue. J Urol 153: 511–515. PMID: 7529338

90. McIntyre A, Summersgill B, Grygalewicz B, Gillis AJ, Stoop J, van Gurp RJ, et al. (2005) Amplification and overexpression of the KIT gene is associated with progression in the seminoma subtype of testicular germ cell tumors of adolescents and adults. Cancer Res 65: 8085–8089. PMID: 16166280

91. Mol CD, Dougan DR, Schneider TR, Skene RJ, Kraus ML, Scheibe DN, et al. (2004) Structural basis for the autoinhibition and STI-571 inhibition of c-Kit tyrosine kinase. J Biol Chem 279: 31655–31663. PMID: 15123710

92. Agarwal S, Kazi JU, Ronnstrand L (2013) Phosphorylation of the activation loop tyrosine 823 in c-Kit is crucial for cell survival and proliferation. J Biol Chem 288: 22460–22468. doi: 10.1074/jbc.M113.474072 PMID: 23803604

93. Fukushima S, Otsuka A, Suzuki T, Yanagisawa T, Mishima K, Mukasa A, et al. (2014) Mutually exclusive mutations of KIT and RAS are associated with KIT mRNA expression and chromosomal instability in primary intracranial pure germinomas. Acta Neuropathol.

94. Kanetsky PA, Mitra N, Vardhanabhuti S, Li M, Vaughn DJ, Letrero R, et al. (2009) Common variation in KITLG and at 5q31.3 predisposes to testicular germ cell cancer. Nat Genet 41: 811–815. doi: 10.1038/ng.393 PMID: 19483682

95. Kanetsky PA, Mitra N, Vardhanabhuti S, Vaughn DJ, Li M, Ciosek SL, et al. (2011) A second independent locus within DMRT1 is associated with testicular germ cell tumor susceptibility. Hum Mol Genet 20: 3109–3117. doi: 10.1093/hmg/ddr207 PMID: 21551455

96. Kratz CP, Han SS, Rosenberg PS, Berndt SI, Burdett L, Yeager M, et al. (2011) Variants in or near KITLG, BAK1, DMRT1, and TERT-CLPTM1L predispose to familial testicular germ cell tumour. J Med Genet 48: 473–476. doi: 10.1136/jmedgenet-2011-100001 PMID: 21617256

97. Kratz CP, Greene MH, Bratslavsky G, Shi J (2011) A stratified genetic risk assessment for testicular cancer. Int J Androl 34: e98-102. doi: 10.1111/j.1365-2605.2011.01156.x PMID: 21564132

98. Rapley EA, Hockley S, Warren W, Johnson L, Huddart R, Crockford G, et al. (2004) Somatic mutations of KIT in familial testicular germ cell tumours. Br J Cancer 90: 2397–2401. PMID: 15150569

99. Rapley EA, Turnbull C, Al Olama AA, Dermitzakis ET, Linger R, Huddart RA, et al. (2009) A genome-wide association study of testicular germ cell tumor. Nat Genet 41: 807–810. doi: 10.1038/ng.394 PMID: 19483681

100. Ruark E, Seal S, McDonald H, Zhang F, Elliot A, Lau K, et al. (2013) Identification of nine new susceptibility loci for testicular cancer, including variants near DAZL and PRDM14. Nat Genet.

101. Schumacher FR, Wang Z, Skotheim RI, Koster R, Chung CC, Hildebrandt MA, et al. (2013) Testicular germ cell tumor susceptibility associated with the UCK2 locus on chromosome 1q23. Hum Mol Genet 22: 2748–2753. doi: 10.1093/hmg/ddt109 PMID: 23462292

102. Turnbull C, Rahman N (2011) Genome-wide association studies provide new insights into the genetic basis of testicular germ-cell tumour. Int J Androl 34: e86-96; discussion e96-87. doi: 10.1111/j.1365-2605.2011.01162.x PMID: 21623831

103. Turnbull C, Rapley EA, Seal S, Pernet D, Renwick A, Hughes D, et al. (2010) Variants near DMRT1, TERT and ATF7IP are associated with testicular germ cell cancer. Nat Genet 42: 604–607. doi: 10.1038/ng.607 PMID: 20543847

104. Zeron-Medina J, Wang X, Repapi E, Campbell MR, Su D, Castro-Giner F, et al. (2013) A polymorphic p53 response element in KIT ligand influences cancer risk and has undergone natural selection. Cell 155: 410–422. doi: 10.1016/j.cell.2013.09.017 PMID: 24120139

105. Azevedo MF, Horvath A, Bornstein ER, Almeida MQ, Xekouki P, Faucz FR, et al. (2013) Cyclic AMP and c-KIT signaling in familial testicular germ cell tumor predisposition. J Clin Endocrinol Metab.

106. Mirabello L, Kratz CP, Savage SA, Greene MH (2012) Promoter methylation of candidate genes associated with familial testicular cancer. Int J Mol Epidemiol Genet 3: 213–227. PMID: 23050052

107. Voorhoeve PM, le Sage C, Schrier M, Gillis AJ, Stoop H, Nagel R, et al. (2006) A genetic screen implicates miRNA-372 and miRNA-373 as oncogenes in testicular germ cell tumors. Cell 124: 1169–1181. PMID: 16564011

108. Gillis AJ, Rijlaarsdam MA, Eini R, Dorssers LC, Biermann K, Murray MJ, et al. (2013) Targeted serum miRNA (TSmiR) test for diagnosis and follow-up of (testicular) germ cell cancer patients: a proof of principle. Mol Oncol 7: 1083–1092. doi: 10.1016/j.molonc.2013.08.002 PMID: 24012110

109. Deb-Rinker P, Ly D, Jezierski A, Sikorska M, Walker PR (2005) Sequential DNA methylation of the Nanog and Oct-4 upstream regions in human NT2 cells during neuronal differentiation. J Biol Chem 280: 6257–6260. PMID: 15615706

110. Nettersheim D, Biermann K, Gillis AJ, Steger K, Looijenga LH, Schorle H (2011) NANOG promoter methylation and expression correlation during normal and malignant human germ cell development. Epigenetics 6: 114–122. doi: 10.4161/epi.6.1.13433 PMID: 20930529

111. Looijenga LH, Stoop H, de Leeuw HP, de Gouveia Brazao CA, Gillis AJ, van Roozendaal KE, et al. (2003) POU5F1 (OCT3/4) identifies cells with pluripotent potential in human germ cell tumors. Cancer Res 63: 2244–2250. PMID: 12727846

112. De Jong J, Weeda S, Gillis AJ, Oosterhuis JW, Looijenga LH (2007) Differential methylation of the OCT3/4 upstream region in primary human testicular germ cell tumors. Oncol Rep 18: 127–132. PMID: 17549357

113. Nordhoff V, Hubner K, Bauer A, Orlova I, Malapetsa A, Scholer HR (2001) Comparative analysis of human, bovine, and murine Oct-4 upstream promoter sequences. Mamm Genome 12: 309–317. PMID: 11309664

114. Manton KJ, Douglas ML, Netzel-Arnett S, Fitzpatrick DR, Nicol DL, Boyd AW, et al. (2005) Hypermethylation of the 5' CpG island of the gene encoding the serine protease Testisin promotes its loss in testicular tumorigenesis. Br J Cancer 92: 760–769. PMID: 15685234

115. Kempkensteffen C, Christoph F, Weikert S, Krause H, Kollermann J, Schostak M, et al. (2006) Epigenetic silencing of the putative tumor suppressor gene testisin in testicular germ cell tumors. J Cancer Res Clin Oncol 132: 765–770. PMID: 16810501

116. Kato N, Tamura G, Fukase M, Shibuya H, Motoyama T (2003) Hypermethylation of the RUNX3 gene promoter in testicular yolk sac tumor of infants. Am J Pathol 163: 387–391. PMID: 12875960

117. Lind GE, Skotheim RI, Lothe RA (2007) The epigenome of testicular germ cell tumors. APMIS 115: 1147–1160. PMID: 18042148

118. Nettersheim D, Gillis A, Biermann K, Looijenga LH, Schorle H (2011) The seminoma cell line TCam-2 is sensitive to HDAC inhibitor depsipeptide but tolerates various other chemotherapeutic drugs and loss of NANOG expression. Genes Chromosomes Cancer 50: 1033–1042. doi: 10.1002/gcc.20918 PMID: 21987446

119. Nettersheim D, Westernstroer B, Haas N, Leinhaas A, Brustle O, Schlatt S, et al. (2012) Establishment of a versatile seminoma model indicates cellular plasticity of germ cell tumor cells. Genes Chromosomes Cancer 51: 717–726. doi: 10.1002/gcc.21958 PMID: 22489004

120. Weber S, Eckert D, Nettersheim D, Gillis AJ, Schafer S, Kuckenberg P, et al. (2010) Critical function of AP-2 gamma/TCFAP2C in mouse embryonic germ cell maintenance. Biol Reprod 82: 214–223. doi: 10.1095/biolreprod.109.078717 PMID: 19776388

121. Hoei-Hansen CE, Nielsen JE, Almstrup K, Sonne SB, Graem N, Skakkebaek NE, et al. (2004) Transcription factor AP-2gamma is a developmentally regulated marker of testicular carcinoma in situ and germ cell tumors. Clin Cancer Res 10: 8521–8530. PMID: 15623634

122. Orso F, Cottone E, Hasleton MD, Ibbitt JC, Sismondi P, Hurst HC, et al. (2004) Activator protein-2gamma (AP-2gamma) expression is specifically induced by oestrogens through binding of the oestrogen receptor to a canonical element within the 5'-untranslated region. Biochem J 377: 429–438. PMID: 14565844

123. Kawakami T, Okamoto K, Ogawa O, Okada Y (2004) XIST unmethylated DNA fragments in male-derived plasma as a tumour marker for testicular cancer. Lancet 363: 40–42. PMID: 14723995

124. Furukawa S, Haruta M, Arai Y, Honda S, Ohshima J, Sugawara W, et al. (2009) Yolk sac tumor but not seminoma or teratoma is associated with abnormal epigenetic reprogramming pathway and shows frequent hypermethylation of various tumor suppressor genes. Cancer Sci 100: 698–708. doi: 10.1111/j.1349-7006.2009.01102.x PMID: 19245437

125. Ueda T, Abe K, Miura A, Yuzuriha M, Zubair M, Noguchi M, et al. (2000) The paternal methylation imprint of the mouse H19 locus is acquired in the gonocyte stage during foetal testis development. Genes Cells 5: 649–659. PMID: 10947850

126. Kerjean A, Dupont JM, Vasseur C, Le Tessier D, Cuisset L, Paldi A, et al. (2000) Establishment of the paternal methylation imprint of the human H19 and MEST/PEG1 genes during spermatogenesis. Hum Mol Genet 9: 2183–2187. PMID: 10958657

127. Kawasaki Y, Lee J, Matsuzawa A, Kohda T, Kaneko-Ishino T, Ishino F (2014) Active DNA demethylation is required for complete imprint erasure in primordial germ cells. Sci Rep 4: 3658. doi: 10.1038/srep03658 PMID: 24413819

128. Zimmerman DL, Boddy CS, Schoenherr CS (2013) Oct4/Sox2 binding sites contribute to maintaining hypomethylation of the maternal igf2/h19 imprinting control region. PLoS One 8: e81962. doi: 10.1371/journal.pone.0081962 PMID: 24324735

129. van Gurp RJ, Oosterhuis JW, Kalscheuer V, Mariman EC, Looijenga LH (1994) Biallelic expression of the H19 and IGF2 genes in human testicular germ cell tumors. J Natl Cancer Inst 86: 1070–1075. PMID: 8021956

130. Verkerk AJ, Ariel I, Dekker MC, Schneider T, van Gurp RJ, de Groot N, et al. (1997) Unique expression patterns of H19 in human testicular cancers of different etiology. Oncogene 14: 95–107. PMID: 9010236

131. Schneider DT, Schuster AE, Fritsch MK, Hu J, Olson T, Lauer S, et al. (2001) Multipoint imprinting analysis indicates a common precursor cell for gonadal and nongonadal pediatric germ cell tumors. Cancer Res 61: 7268–7276. PMID: 11585765

132. Ross JA, Schmidt PT, Perentesis JP, Davies SM (1999) Genomic imprinting of H19 and insulin-like growth factor-2 in pediatric germ cell tumors. Cancer 85: 1389–1394. PMID: 10189147

133. Kawakami T, Zhang C, Okada Y, Okamoto K (2006) Erasure of methylation imprint at the promoter and CTCF-binding site upstream of H19 in human testicular germ cell tumors of adolescents indicate their fetal germ cell origin. Oncogene 25: 3225–3236. PMID: 16434968

134. Bussey KJ, Lawce HJ, Himoe E, Shu XO, Heerema NA, Perlman EJ, et al. (2001) SNRPN methylation patterns in germ cell tumors as a reflection of primordial germ cell development. Genes Chromosomes Cancer 32: 342–352. PMID: 11746975

135. Smiraglia DJ, Szymanska J, Kraggerud SM, Lothe RA, Peltomaki P, Plass C (2002) Distinct epigenetic phenotypes in seminomatous and nonseminomatous testicular germ cell tumors. Oncogene 21: 3909–3916. PMID: 12032829

136. Kristensen DG, Nielsen JE, Jorgensen A, Skakkebaek NE, Rajpert-De Meyts E, Almstrup K (2014) Evidence that active demethylation mechanisms maintain the genome of carcinoma in situ cells hypomethylated in the adult testis. Br J Cancer 110: 668–678. doi: 10.1038/bjc.2013.727 PMID: 24292451

137. Sonne SB, Perrett RM, Nielsen JE, Baxter MA, Kristensen DM, Leffers H, et al. (2010) Analysis of SOX2 expression in developing human testis and germ cell neoplasia. Int J Dev Biol 54: 755–760. doi: 10.1387/ijdb.082668ss PMID: 19876845

138. Kristensen DM, Sonne SB, Ottesen AM, Perrett RM, Nielsen JE, Almstrup K, et al. (2008) Origin of pluripotent germ cell tumours: the role of microenvironment during embryonic development. Mol Cell Endocrinol 288: 111–118. doi: 10.1016/j.mce.2008.02.018 PMID: 18420341

139. Honecker F, Stoop H, Mayer F, Bokemeyer C, Castrillon DH, Lau YF, et al. (2006) Germ cell lineage differentiation in non-seminomatous germ cell tumours. J Pathol 208: 395–400. PMID: 16273510

140. Yang S, Lin G, Deng L, Lu GX (2012) Tumourigenic characteristics of embryonal carcinoma cells as a model for studying tumour progression of human embryonic stem cells. Cell Prolif 45: 299–310. doi: 10.1111/j.1365-2184.2012.00827.x PMID: 22731741

141. Josephson R, Ording CJ, Liu Y, Shin S, Lakshmipathy U, Toumadje A, et al. (2007) Qualification of embryonal carcinoma 2102Ep as a reference for human embryonic stem cell research. Stem Cells 25: 437–446. PMID: 17284651

142. Andrews PW, Matin MM, Bahrami AR, Damjanov I, Gokhale P, Draper JS (2005) Embryonic stem (ES) cells and embryonal carcinoma (EC) cells: opposite sides of the same coin. Biochem Soc Trans 33: 1526–1530. PMID: 16246161

143. Kristensen DG, Mlynarska O, Nielsen JE, Jacobsen GK, Rajpert-De Meyts E, Almstrup K (2012) Heterogeneity of chromatin modifications in testicular spermatocytic seminoma point toward an epigenetically unstable phenotype. Cancer Genet 205: 425–431. doi: 10.1016/j.cancergen.2012.05.003 PMID: 22819380

144. Andrews PW, Bronson DL, Benham F, Strickland S, Knowles BB (1980) A comparative study of eight cell lines derived from human testicular teratocarcinoma. Int J Cancer 26: 269–280. PMID: 6169654

145. Andrews PW, Casper J, Damjanov I, Duggan-Keen M, Giwercman A, Hata J, et al. (1996) Comparative analysis of cell surface antigens expressed by cell lines derived from human germ cell tumours. Int J Cancer 66: 806–816. PMID: 8647654

146. Wang N, Trend B, Bronson DL, Fraley EE (1980) Nonrandom abnormalities in chromosome 1 in human testicular cancers. Cancer Res 40: 796–802. PMID: 7471097

147. Fogh J, Trempe G (1975) New Human Tumor Cell Lines. In: Fogh J, editor. Human Tumor Cells in Vitro: Springer US. pp. 115–159.

148. Fogh J (1978) Cultivation, characterization, and identification of human tumor cells with emphasis on kidney, testis, and bladder tumors. Natl Cancer Inst Monogr: 5–9. PMID: 571047

149. Teshima S, Shimosato Y, Hirohashi S, Tome Y, Hayashi I, Kanazawa H, et al. (1988) Four new human germ cell tumor cell lines. Lab Invest 59: 328–336. PMID: 2842544

150. Eckert D, Nettersheim D, Heukamp LC, Kitazawa S, Biermann K, Schorle H (2008) TCam-2 but not JKT-1 cells resemble seminoma in cell culture. Cell Tissue Res 331: 529–538. PMID: 18008088

151. de Jong J, Stoop H, Gillis AJ, Hersmus R, van Gurp RJ, van de Geijn GJ, et al. (2008) Further characterization of the first seminoma cell line TCam-2. Genes Chromosomes Cancer 47: 185–196. PMID: 18050305

152. Mizuno Y, Gotoh A, Kamidono S, Kitazawa S (1993) [Establishment and characterization of a new human testicular germ cell tumor cell line (TCam-2)]. Nihon Hinyokika Gakkai Zasshi 84: 1211–1218. PMID: 8394948

153. Ito S, Shen L, Dai Q, Wu SC, Collins LB, Swenberg JA, et al. (2011) Tet proteins can convert 5-methylcytosine to 5-formylcytosine and 5-carboxylcytosine. Science 333: 1300–1303. doi: 10.1126/science.1210597 PMID: 21778364

154. Du P, Kibbe WA, Lin SM (2008) lumi: a pipeline for processing Illumina microarray. Bioinformatics 24: 1547–1548. doi: 10.1093/bioinformatics/btn224 PMID: 18467348

155. Marabita F, Almgren M, Lindholm ME, Ruhrmann S, Fagerstrom-Billai F, Jagodic M, et al. (2013) An evaluation of analysis pipelines for DNA methylation profiling using the Illumina HumanMethylation450 BeadChip platform. Epigenetics 8: 333–346. doi: 10.4161/epi.24008 PMID: 23422812

156. Chen YA, Lemire M, Choufani S, Butcher DT, Grafodatskaya D, Zanke BW, et al. (2013) Discovery of cross-reactive probes and polymorphic CpGs in the Illumina Infinium HumanMethylation450 microarray. Epigenetics 8: 203–209. doi: 10.4161/epi.23470 PMID: 23314698

157. Teschendorff AE, Marabita F, Lechner M, Bartlett T, Tegner J, Gomez-Cabrero D, et al. (2013) A beta-mixture quantile normalization method for correcting probe design bias in Illumina Infinium 450 k DNA methylation data. Bioinformatics 29: 189–196. doi: 10.1093/bioinformatics/bts680 PMID: 23175756

158. Benjamini Y, Hochberg Y (1995) Controlling the False Discovery Rate: A Practical and Powerful Approach to Multiple Testing. Journal of the Royal Statistical Society Series B (Methodological) 57: 289–300.

159. Tibshirani (1996) Regression Shrinkage and Selection via the Lasso. J R Statist Soc 58: 267–288.

160. Rijlaarsdam MA, van der Zwan YG, Dorssers LC, Looijenga LH (2014) DMRforPairs: identifying differentially methylated regions between unique samples using array based methylation profiles. BMC Bioinformatics 15: 141. doi: 10.1186/1471-2105-15-141 PMID: 24884391
